# Supplementary material for: Feasibility trial of a digital self-management intervention ‘My Breathing Matters’ to improve asthma-related quality of life for UK primary care patients with asthma
Source: BMJ Open. 2019 Nov 12;9(11):e032465. doi: 10.1136/bmjopen-2019-032465 (PMC6858238; doi:10.1136/bmjopen-2019-032465)
Supplement: Supplementary data [file bmjopen-2019-032465supp001.pdf]

National Institute for Health Research

University of Southampton

Patient Costs and Opportunities Questionnaire

Please complete the below questionnaire by ticking ONE box to answer each question.

1. In the last 12 months, have you signed up to any new programmes (e.g. gym membership, taken up a new activity like yoga/meditation) that might help your health?
   - ☐ No new programmes at all
   - ☐ Some new programmes
   - ☐ Lots of new programmes

Please note any programmes you took up:

2. Have any new programmes that you have signed up to (e.g. gym membership, taken up a new activity) cost you any money that you might have spent elsewhere?
   - ☐ Yes — Total Amount: ______ Weekly amount: ______ (if you don't know, please estimate)
   - ☐ No

Please only answer the below questions if you registered with the My Breathing Matters website in the last 12 months.

3. Did you think there were any benefits of using My Breathing Matters?
   - ☐ No benefit at all
   - ☐ Very little benefit
   - ☐ Some benefit
   - ☐ Quite a bit of benefit
   - ☐ A large amount of benefit

If any benefits, please note them down below:

4. Did you think there were any disadvantages of using My Breathing Matters?
   - ☐ No disadvantages at all
   - ☐ Very little disadvantages
   - ☐ Some disadvantages
   - ☐ Quite a bit of disadvantages
   - ☐ A large amount of disadvantages

If any disadvantages, please note them down below:

5. How likely are you to recommend My Breathing Matters to friends and family if they needed similar care and treatment?
   - ☐ Extremely likely
   - ☐ Likely
   - ☐ Neither likely or unlikely
   - ☐ Unlikely
   - ☐ Extremely unlikely
   - ☐ Don't know

MBM Feasibility Consent Form Version 1, 01/11/16

Study team contact details: << insert local co-ordinator details here>> IRAS: 214457
