# Supplementary material for: Feasibility trial of a digital self-management intervention ‘My Breathing Matters’ to improve asthma-related quality of life for UK primary care patients with asthma
Source: BMJ Open. 2019 Nov 12;9(11):e032465. doi: 10.1136/bmjopen-2019-032465 (PMC6858238; doi:10.1136/bmjopen-2019-032465)
Supplement: Supplementary data [file bmjopen-2019-032465supp003.pdf]

My Breathing Matters – Trial Protocol

'My Breathing Matters' - feasibility study of a digital self-management programme designed to improve the quality of life people with asthma.

Version 2, dated 07/12/2016

SPONSOR: University of Southampton

COORDINATING CENTRE: Centre for Applications of Health Psychology (supported by Southampton Clinical Trials Unit)

EudraCT reference no:	N/A
ISRCTN reference no:	TBC
**NHS Ethics reference no:**	**214457**

Protocol authorised by:

**Name:**	Prof. Lucy Yardley	**Role:**	Chief Investigator
**Signature:**	L Yardley	**Date:**	**07/12/2016**

MAIN TRIAL CONTACT

Chief Investigator: Professor Lucy Yardley

Centre for Applications of Health Psychology
Academic unit of psychology
University of Southampton
Southampton Tel: +44 (0)2380 594581
SO17 1BJ Email: L.Yardley@soton.ac.uk

TRIAL COORDINATION CENTRE

For general trial and clinical queries e.g. participant queries, trial supplies, data collection, please contact in the first instance:

Ben Ainsworth

Address: Centre for Applications of Health Psychology
Academic unit of psychology Tel:
University of Southampton Fax: 02380 591950
Southampton Email: ben.ainsworth@soton.ac.uk
SO17 1BJ Web:

SPONSOR

The University of Southampton is the research sponsor for this trial. For further information regarding sponsorship conditions, please contact:

Address: Diana Galpin
Head of IP, Contracts & Policy
University of Southampton
Southampton
SO17 1BJ

Tel: 02380 595058
Email: rgoinfo@soton.ac.uk

FUNDER

This trial is funded by NIHR PGfAR

Protocol Information

This protocol describes the My Breathing Matters trial and provides information about procedures for entering participants. The protocol should not be used as a guide for the treatment of other participants; every care was taken in its drafting, but corrections or amendments may be necessary. These will be circulated to investigators in the trial, but sites entering participants for the first time are advised to contact the Centre for Applications of Health Psychology to confirm they have the most recent version.

Compliance

This trial will adhere to the principles outlined in the International Conference on Harmonisation Good Clinical Practice (ICH GCP) guidelines. It will be conducted in compliance with the protocol, the Data Protection Act and all other regulatory requirements, as appropriate.

TABLE OF CONTENTS

LIST OF ABBREVIATIONS 4

KEYWORDS 4

TRIAL SYNOPSIS 5

SCHEDULE OF OBSERVATIONS AND PROCEDURES 8

1. INTRODUCTION 9

1.1 BACKGROUND 9

1.2 RATIONALE AND RISK BENEFITS FOR CURRENT TRIAL 9

2. TRIAL OBJECTIVES 10

3. TRIAL DESIGN 10

3.1 TRIAL OUTCOME MEASURES 11

4. CENTRE/PARTICIPANT SELECTION AND ENROLLMENT OF PARTICIPANTS 12

4.1 CENTRE SELECTION 12

4.2 SCREENING AND PRE-REGISTRATION 13

4.3 INCLUSION CRITERIA 13

4.4 EXCLUSION CRITERIA 14

4.5 REGISTRATION / RANDOMISATION PROCEDURES 14

4.6 CONSENT 14

4.7 WITHDRAWAL CRITERIA 15

6. INTERVENTION 15

6.1 INTERVENTION ARM 15

6.2 USUAL CARE 16

7. ADVERSE EVENTS AND REPORTING 16

7.1 DEFINITIONS 16

7.2 CAUSALITY 17

7.3 REPORTING PROCEDURES 17

7.3.2. Non serious AEs 17

7.3.3 Serious AEs 18

8. ASSESSMENT AND FOLLOW-UP OF PARTICIPANTS 19

8.1 DEFINITION OF END OF TRIAL 19

9. QUANTITATIVE EVALUATION 19

9.1 SAMPLE SIZE 19

9.2 RANDOMISATION 19

9.3 STATISTICAL PLAN INCLUDING INTERIM ANALYSIS 20

10. QUALITATIVE EVALUATION 20

11. REGULATORY ISSUES 21

11.1 CLINICAL TRIAL AUTHORISATION 21

11.2 ETHICS APPROVAL 21

11. 3 CONFIDENTIALITY 21

Ainsworth B, *et al. BMJ Open* 2019; 9:e032465. doi: 10.1136/bmjopen-2019-032465

11.4 INDEMNITY 22

11.5 SPONSOR 22

11.6 FUNDING 22

11.7 DEVIATIONS AND SERIOUS BREACHES 22

11.8 AUDITS AND INSPECTIONS 23

12. TRIAL MANAGEMENT 23

13. PUBLICATION POLICY 23

14. REFERENCES 24

LIST OF ABBREVIATIONS

AE	Adverse Event
BMQ	Beliefs about Medication Questionnaire
CRF	Case Report Form
CTCAE	Common Terminology Criteria for Adverse Events
DI	Digital Intervention
DMEC	Data Monitoring and Ethics Committee
EQ-5D	EuroQol (health-related quality of life)
HCP	Healthcare Practitioner
ICH GCP	International Conference on Harmonisation of Good Clinical Practice
IMP	Investigational Medicinal Product
MARS	Medication Adherence Report
MBM	My Breathing Matters
NIHR	National Institute for Health Research
PCRN	Primary Care Research Network
PGfAR	Programme Grants for Applied Research
QIPP	Quality, Innovation, Productivity and Prevention
R&D	Research & Development
REC	Research Ethics Committee
SAE	Serious Adverse Event
SSI	Site Specific Information
TMG	Trial Management Group
TSC	Trial Steering Committee

KEYWORDS

Asthma; Self-management; Digital intervention

TRIAL SYNOPSIS

Title:	My Breathing Matters trial protocol
Sponsor:	Southampton University
Sponsor Ref Number:	TBC
Funder:	NIHR PGfAR
Trial Phase:	Complex intervention phase 1
Indication:	Asthma
Primary Objective:	1. To assess the feasibility and acceptability of a trial of My Breathing Matters (MBM) (a digital intervention designed to improve quality-of-life outcomes for people with asthma).
Secondary Objectives:	1. To assess feasibility of trial procedures including: clinical research governance, recruitment strategy, trial documents (e.g. PIS), eligibility criteria, consent/withdrawal, randomisation & blinding.<br>2. To assess feasibility and acceptability of MBM intervention including: usage & engagement, adherence and completion, fidelity of providers, participant retention.<br>3. To assess feasibility of data collection and analysis procedures, and look at data quality, management of trial data and estimates of effect size across trial outcome measures (see primary/secondary endpoints) to inform sample size calculations for a larger phase 3 RCT.
Tertiary Objectives	1. To examine intervention users' usage, engagement and possible mediators of behaviour change to inform future intervention design for well-powered usage and process analyses.
Trial Design:	Individually randomised controlled trial (assessor blinded)
Sample size : (split by treatment group)	80 participants (40 per arm)
Inclusion Criteria:	1. Age 18+ years.<br>2. Physician diagnosed asthma in medical record<br>3. ≥1 anti-asthma medication prescription in the previous year (determined from the physician prescribing records)<br>4. Impaired asthma-related health status (Asthma Quality of Life Questionnaire score of <5.5)<br>5. Informed consent<br>6. Able to access the internet and understand written english.
Exclusion Criteria:	1. Asthma judged at the baseline assessment to be dangerously unstable and in need of urgent medical review (if unstable asthma is found, the patient will be referred back to usual primary care clinician for review)<br>2. Terminal disease or other condition which in the opinion of the family doctor makes them inappropriate to take part<br>3. Diagnosed with 'difficult asthma' as defined by BTS.<br>4. Documented diagnosis of Chronic Obstructive Pulmonary Disease (COPD)

Ainsworth B, *et al. BMJ Open* 2019; 9:e032465. doi: 10.1136/bmjopen-2019-032465

	5. Household member already enrolled on the study
Intervention	My Breathing Matters
Control Group:	Usual care
Primary Endpoints:	Feasibility and acceptability of intervention and trial procedures, including:<br>1. Uptake<br>2. Adherence<br>3. Completion rates
Secondary Endpoints:	Feasibility of measuring (and estimates of effect size to perform sample size calculations) in the following trial measures:<br>1. Asthma-specific Quality of life (AQLQ; short version)<br>2. Asthma control (ACQ)<br>3. Lung function (FEV_1, FEV_1/FVC, PEFR)<br>4. Quality of life (EQ5D, ICECAP-A)<br>5. Anxiety and depression (HADS)<br>6. Patient enablement (PEI)<br>7. Patient burden – time and costs.<br>8. Health resource use (professional contacts, referrals, prescriptions)<br>9. Adherence to recommendations
Tertiary Endpoints	Measurement of patient intervention group only:<br>1. Lifestyle change choice<br>2. Intervention usage and progress<br>3. Engagement with program and reasons for such engagement.
Follow up duration	12 months
Total Number of Sites :	Pilot: 4-6 sites

Ainsworth B, et al. BMJ Open 2019; 9:e032465. doi: 10.1136/bmjopen-2019-032465

Participant flow diagram

Practices identified by the PCRN:

- 6 practices diversely sampled by size, rural-urban location and Index of Multiple Deprivation

Patient identification:

- Practice database searches and letter of invitation and PIS sent by practice
- Opportunistic recruitment via consultations with GP or practice nurse
- Study advertisement posters within practices
- Screen approximately 10-20 patients per practice (estimated mail-out response rate)

Baseline consultation with Research nurse:

- Consent, height, weight (BMI)
- Confirm asthma diagnosis via practice if recruited via poster.
- Questionnaire measures (paper copy)
- Patients given instructions for Lifeguide registration
- Enrol approx. 5-10 patients per practice (total N = 60)
- Randomisation (1:1).

Online registration and randomisation:

- Standardised advice given to all patients (Asthma UK information leaflet)
- Management decisions made by usual clinicians in GP practice as per usual care.

Intervention group (DI ; N = 40)

Patient has access to My Breathing Matters

- Online training in appropriate asthma medication use and access to self-management support
- Online breathing retraining
- Access to online lifestyle modification programs within intervention (physical activity, weight reduction, smoking cessation)
- Resource use monitored.
- Qualitative interviews to discuss experience of intervention

Usual Care (N=40)

- Resource use monitored.

Questionnaire assessment (3 months)

- To be completed via post; second pack will be sent out after 2 weeks if no response; AQLQ completed over the phone with blinded researcher if still outstanding after a further 2 weeks.

Outcome assessment with research nurse (12 months):

- Quality of life and clinical measures (measured by blinded research nurse)
- AQLQ to be completed over phone or via postal pack if not willing to attend)

Ainsworth B, *et al. BMJ Open* 2019; 9:e032465. doi: 10.1136/bmjopen-2019-032465

SCHEDULE OF OBSERVATIONS AND PROCEDURES

	Time point			
Measure	Baseline/ Screening	3-month follow up	Followup	Within the MBM programme
**Month**	0	3	12	(0-12)
Patient socio-demographic measures	X			
Clinical measures				
Weight (kg)	X	X	X	
Height (cm)	X			
Medication changes (prescriptions issued)			X (NR)	
Consultations			X (NR)	
Patient self-report measures				
Patient Enablement Instrument	X	X	X	
Asthma-specific Quality of life	X	X	X	
Asthma control	X	X	X	
Anxiety & depression	X	X	X	
Quality of life	x	X	X	
Medication adherence	X	X	X	
Lung function	X		X	
Medication use	X		X	
Patient objectively recorded measures				
Website usage				X*
Usage of asthma action plan (PAAP)				X*
Usage of asthma review pages				X*
Booked asthma review				X*
Usage of '4-week medication challenge'				X*
Usage of medication information				X*
Usage of breathing retraining challenge				X*
Usage of friends & family section				X*
Usage of stress reduction				X*
Choice of lifestyle changes				X*
Reported progress on lifestyle change (e.g. weight change)				X*
Economic measures				
Patient quality of life	X	X	X	
Patient costs	X	X	X	
Patient time and resource use			X (NR)	
Qualitative analysis				
Patient experience and views of the DI				X

Key:

NR = Notes review

*My Breathing Matters arm only – measured via Lifeguide website

1 This will be measured directly before and after the My Breathing Matters training completion

Ainsworth B, *et al. BMJ Open* 2019; 9:e032465. doi: 10.1136/bmjopen-2019-032465

1. INTRODUCTION

1.1 BACKGROUND

The UK has one of the highest prevalence's of asthma in the world; nearly 6% of the UK population have asthma, comprising 5.4 million people, most of whom are managed in primary care[1]. Hospital admission and mortality rates for asthma showed improvements in the last decades of the last century, but these improvements have stalled since the millennium. Premature mortality from asthma was 1.5 times as high in the UK as in the rest of the EU in 2008, with around 1000 to 1200 deaths a year recorded since 2000. It is estimated that 90% of deaths are associated with preventable factors. Asthma is associated with high numbers of admissions and Emergency Department attendances, and it is estimated that 70% of these could have been prevented by appropriate early intervention and self-management[1]. Surveys of asthma symptoms and health status impairment continue to show that sub-optimal control is common and that at any given time the majority of asthmatics in the UK suffer potentially avoidable symptoms and quality of life impairment[2].

Although the UK leads the world in providing guidelines for asthma management, these have been poorly implemented and people with asthma do not receive evidence-based interventions, particularly individual action plans, which are known to impact positively on outcomes[3]. Patient education and proactive self-management have been convincingly shown to improve clinical outcomes in asthma[3] and have been advocated in guidelines for 20 years[4]. People with asthma without a management plan are four times more likely to have an asthma attack needing emergency care in hospital[5]. However, a representative (Ipsos-MORI) survey carried out by Asthma UK in 2010[6] suggested that only a quarter of people with asthma in the UK have a self-management plan. Self-management in asthma can also encompass non-pharmacological interventions to improve control and empower the patient, such as breathing exercises or lifestyle changes such as smoking cessation and weight reduction (since smoking and obesity are associated with worse prognosis in asthma[7]).

1.2 RATIONALE AND RISK BENEFITS FOR CURRENT TRIAL

Increasingly widespread access to the internet and mobile phones [8,9] means that healthcare Digital Interventions (DIs) are accessible to the majority of patients, and can be used to provide information and support at any time the patient needs it [10]. DIs can empower patients by providing better access to personalised information, and support for active involvement in treatment and self-management [10]. A large meta-analysis found a small but significant positive effect of DIs on health-related behaviours [11], whilst a Cochrane review found evidence that computer-based health interventions for those with chronic health conditions significantly improved knowledge, health behaviours and clinical outcomes [12]. DIs have the potential to make significant savings by automating routine aspects of patient education, monitoring and support, freeing up health professional resources for when patients most need them[13]. These savings can play an essential part in meeting the NHS QIPP agenda to achieve increased efficiency gains despite the growing demand created by an expanding and ageing population.

Ainsworth B, *et al. BMJ Open* 2019; 9:e032465. doi: 10.1136/bmjopen-2019-032465

There is accumulating evidence that DIs can deliver better and more efficient healthcare in the context of asthma. A recent systematic review by our group found that self-management DIs could improve asthma control and reduce asthma-related quality of life impairment but called for larger, more robust trials[14]. We identified several existing self-management DIs for asthma, but half of these were for children, and many were not in English. While there are numerous commercial DIs for asthma, only one has been evaluated[15]. The RAISIN pilot trial demonstrated that self-management interventions could be effective at improving quality of life and asthma control, with improvements to 'reach' and response rate by catering to patients with mild asthma but impaired quality of life through non-pharmacological means [16,17]. A Danish trial compared GP and specialist care with web-based self-monitoring with automated feedback and a stepped care medication plan (with GP advice when required)[15]. After six months those allocated to the web-based self-monitoring had greater improvement in symptoms, quality of life and lung function. The SMASHING trial in the Netherlands[18] compared usual care with web-based educational resources, self-monitoring and automated feedback on medication titration, plus some group and email nurse support. At one year the intervention group had better quality of life and lung function and more symptom free days, at no extra cost[19].

2. TRIAL OBJECTIVES

The primary aim of the My Breathing Matters trial is to assess the feasibility, acceptability, effectiveness and cost-effectiveness of a DI in primary care for the self-management of asthma, in comparison to usual care (with provision of standard patient information materials produced by the charity Asthma UK).

Main research question:

1. To assess the feasibility and acceptability of a trial of My Breathing Matters, an intervention designed to assess improvements in clinical outcomes (e.g. quality of life, health resource use, lung function) of people with asthma.

Secondary research questions

1. To assess feasibility of trial procedures including: clinical research governance, recruitment strategy, trial documents (e.g. PIS), eligibility criteria, consent/withdrawal, randomisation and blinding.
2. To assess feasibility and acceptability of MBM intervention including: usage and engagement, adherence and completion, fidelity of providers.
3. To assess feasibility of data analysis, including data collection, data quality, management of trial data and estimates of effect size across trial outcome measures (see primary/secondary endpoints) to inform sample size calculations for a larger phase 3 RCT.

Tertiary research questions

1. To examine intervention usage, progress and engagement to inform well-powered usage analysis in larger trial

3. TRIAL DESIGN

My Breathing Matters is a digital intervention for the self-management of asthma, consisting of pharmacological support, (advice about asthma reviews and personal asthma action plans, information about medication and side effects) and non-pharmacological components (stress reduction, online versions of breathing retraining courses shown to be acceptable and feasible[20]) and optional user-selected lifestyle modifications. 80 participants will be randomised either to the My Breathing Matters programme or to a control group receiving usual care with provision of an Asthma UK information leaflet. This trial will be coordinated from UK facilitated by the PCRN's in these areas with researchers and research nurses employed at each centre.

The MBM study will comprise:
1) **Feasibility trial**: 80 participants (40 per arm) will be recruited from practices to confirm the acceptability and feasibility of the intervention, full trial protocol and study procedures.
2) **Qualitative analysis** will also be embedded in to the My Breathing Matters study.

3.1 TRIAL OUTCOME MEASURES

The primary outcome of the trial will be the feasibility and acceptability of a trial of My Breathing Matters, an intervention designed to improve clinical outcomes (e.g. symptom control, quality of life) of people with asthma.

Secondary outcomes will be to assess the feasibility of measuring (and estimates of effect size to generate hypotheses and perform sample size calculations with which to test them) in the following trial measures:
1. Asthma-specific Quality of life (AQLQ; short version)
2. Asthma control (ACQ)
3. Lung function (FEV_1, FEV_1/FVC, PEFR)
4. Quality of life (EQ5D)
5. Anxiety and depression (HADS)
6. Patient enablement (PEI)
7. Perceived support
8. Costs of equipment and drugs
9. Health resource use (professional contacts, referrals, prescriptions)
10. Adherence to recommendations
11. Engagement with program and reasons for such engagement

3-month questionnaire packs will be mailed out with a freepost return envelope. Baseline and 12-month follow up questionnaires will be taken using paper questionnaire packs and clinical measures will be taken by a research nurse.

For 3 month and 12-month measures, a second pack will be sent out after 2 weeks if no response; followed by questionnaire measures (AQLQ) completed over the phone if still outstanding after a further 2 weeks.

<u>Further detail regarding clinical measures:</u>
- Lung function will be measured using spirometry by an appropriately trained research nurse. Measures of lung function will be: FEV_1 (forced expiratory volume in 1 second), FEV_1/FVC (ratio of FEV_1 to forced vital capacity), PEFR (peak expiratory flow rate).

Ainsworth B, et al. BMJ Open 2019; 9:e032465. doi: 10.1136/bmjopen-2019-032465

- Medication prescriptions, any changes and support provision (consultations) will be identified from patient notes reviews after completion of the 12-month follow-up. Medication use will be converted into defined daily doses of medication.

Further detail regarding patient reported measures:

- Asthma-specific quality of life (AQLQ) at 12-month follow-up
- Asthma symptoms will be measured using the Asthma Control Questionnaire (ACQ)
- Anxiety and depression will be measured using the Hospital Anxiety and Depression inventory (HADS)
- Patient enablement (PEI): will be measured using the Patient Enablement Instrument [31].
- Patient medication adherence: all participants will complete the MARS as a measure of medication adherence [2].

In the event that the results from any of the questionnaires are of concern (e.g. demonstrate significant anxiety or depression), the research team will inform the participant of their scores and the normal range of scores, and suggest that they may wish to discuss this result with their GP.

Economic measures:
- Health resource use (professional contacts, referrals, prescriptions)
- Patient time/burden needed to take part in intervention
- Health-related quality of life: the EuroQol (EQ-5D 5L) provides a measure of quality of life for economic analysis[5] and the ICEACAP-A measures non-health-specific quality of life in adults[21].
- Patient costs (e.g. additional costs incurred if patient engages in extra activities beyond intervention, such as gym membership)

Tertiary outcomes (intervention group only) will include:

- Lifestyle change choice, usage and progress
- Adherence to recommendations
- Engagement with program and reasons for such engagement

All measured over the 12 months of the study.
Assessed at baseline, after 3 months and after 12 months of online training.

Socio-demographic data
- Participants' age, gender, internet experience, disease status (time diagnosed), smoking habits, education level, and social deprivation indices based on postcode will be recorded at baseline

4. CENTRE/PARTICIPANT SELECTION AND ENROLLMENT OF PARTICIPANTS

4.1 CENTRE SELECTION

Ainsworth B, *et al. BMJ Open* 2019; 9:e032465. doi: 10.1136/bmjopen-2019-032465

Eligible participants will be identified from around 6 general practices located by primary care research networks (PCRN); Practices will be selected in order to confirm feasibility for a wider trial. Principal Investigators will be identified at the regional level, with lead GPs nominated at each site.

The following documents must be in place and copies sent to the MBM Clinical Trial Coordinator (CTC) (see contact details on page 2):

- The approval letter from the relevant R&D Department, following submission of the Site Specific Information (SSI) form (where required)
- A signed Study Agreement (PI and sponsor signature)
- Completed Signature List and Roles and Responsibilities document
- Completed contacts list of all site personnel working on the Study

Upon receipt of all the above documents, the MBM CTC will send a confirmation letter to the lead GP. This letter must be filed in each centre's Site File. Along with this confirmation letter, the practice should receive their trial supplies and a study pack holding all the documents required to recruit a patient into the MBM Trial.

4.2 SCREENING AND PRE-REGISTRATION

Screening

- Electronic database searches will be conducted, and records screened by general practitioners to remove participants who meet exclusion criteria. The latest version of the participant information sheets along with the consent and screening questionnaire (including a freepost response envelope) will be posted with a letter inviting the patient to take part in the study;
- Eligible patients may also be identified and referred opportunistically during routine consultations with the practice nurse or GP.
- Study advertisements will be displayed in participating practices;
    - Patients responding to the study advertisements will phone the research team who will conduct a minimal screening of participants by telephone using a standard set of questions (included as a supporting document) to establish whether they meet the basic inclusion criteria, e.g. diagnosis of asthma, access to internet. The research team will then send copies of patient study materials (consent form and questionnaire) to confirm eligibility.

Patients who do not respond within one month of the practice invite will be followed up by telephone. This procedure will be explicitly outlined in the invite letter sent from the practice. In addition, all invited participants will be provided with a response form to return should they wish to decline to take part in the trial. This will ask for basic demographic information and their reasons for declining, including an option not to give a reason if they prefer not to (see patient opt-out).

4.3 INCLUSION CRITERIA

1. Physician diagnosed asthma in medical record (confirmed via practice)
2. ≥1 anti-asthma medication prescription in the previous year (determined from the physician prescribing records)

Ainsworth B, *et al. BMJ Open* 2019; 9:e032465. doi: 10.1136/bmjopen-2019-032465

3. Impaired asthma-related health status (Asthma Quality of Life Questionnaire score of <5.5)
4. Informed consent
5. Able to understand English and access internet.

4.4 EXCLUSION CRITERIA

6. Asthma judged at the baseline assessment to be dangerously unstable and in need of urgent medical review (if unstable asthma is found, the patient will be referred back to usual primary care clinician for review)
7. Terminal disease or other condition which in the opinion of the family doctor makes them inappropriate to take part
8. Diagnosed with 'difficult asthma' as defined by BTS.
9. Documented diagnosis of Chronic Obstructive Pulmonary Disease (COPD)
10. Household member already enrolled on the study

4.5 REGISTRATION / RANDOMISATION PROCEDURES

Informed consent
All patients will receive a copy of the consent form in their invitation letter pack, and will be asked to complete it when contacting the research team. All patients will receive a face-to-face baseline consultation with the practice nurse where additional informed consent (see consent form) will be recorded before clinical measures, such as confirmation of asthma diagnosis, are undertaken (see primary outcomes data document).

Randomisation
Patients will be randomised at the practice where if in the intervention group they will be given access to the My Breathing Matters programme, or if in the control group will be given Asthma UK information leaflet. (see section 9.2 for further details of randomisation). Participants in the intervention group will be instructed to sign up to the My Breathing Matters programme upon which they will be notified by email of their involvement in the study. Only one patient per household will be randomised.

4.6 CONSENT

Consent to enter the trial must be sought from each participant only after a full explanation has been given, an information leaflet offered and time allowed for consideration. Signed participant consent will be obtained. The right of the participant to refuse to participate without giving reasons will be respected. After the participant has entered the trial the clinician remains free to give alternative treatment to that specified in the protocol at any stage if he/she feels it is in the participant's best interest, but the reasons for doing so should be recorded. In these cases the participants remain within the trial for the purposes of follow-up and data analysis. All participants are free to withdraw at any time from the protocol intervention without giving reasons and without prejudicing further treatment.
Participants will give informed consent (including explicit consent to access relevant data from the DI and their medical records) before they first log onto MBM. They will be free to withdraw at any time simply by contacting the research team or practice staff

Ainsworth B, *et al. BMJ Open* 2019; 9:e032465. doi: 10.1136/bmjopen-2019-032465

4.7 WITHDRAWAL CRITERIA

Patients will be withdrawn from the trial if:

- They are no longer eligible (specialist management of Asthma or illness precluding participation)
- They choose not to continue

All patients who withdraw from the intervention will be asked if they are prepared to attend the final follow-up appointment with the research nurse and/or complete self-report follow-up measures. If they agree, they will be invited according to the follow-up procedure and will have the option to answer follow up questionnaires without using the website.

.

6. INTERVENTION

6.1 INTERVENTION ARM

The **online My Breathing Matters programme** will comprise **three** main components:

1. *Intervention components*

The MBM programme will use behavioural techniques to improve functional quality of life of primary care patients with asthma, by supporting illness self-management by pharmacological and non-pharmacological means (thereby reducing risk of asthma exacerbation).

The program has three main design objectives: i) to engage people who do not view themselves as having active asthma, ii) to persuade and educate users to implement appropriate pharmacological management and iii) to encourage users to employ non-pharmacological methods of improving QoL. Key features to address these objectives include: i) maintaining positive illness context throughout (i.e. promote health rather than manage illness) and offering a simple, unobtrusive interface to provide optional (and flexible) support only when needed, ii) focusing on persuading/educating users regarding the necessity, efficacy and safety of preventative asthma medication, and facilitating easy completion of an action plan with primary care support, and iii) educating users on benefits and offer psychological methods to improve quality of life (e.g. cognitive behavioural techniques for symptom management), tailored access and address patient concerns about relevant positive lifestyle changes, such as weight-loss if overweight, smoking cessation if current smoker, physical activity if inactive.

Patients in the intervention group will be offered optional support from Asthma UK helpline – as a source of asthma advice and support for those who would like personal contact outside of that offered by MBM or their healthcare team, to support self-monitoring and lifestyle modification. This support would be the same as that offered to anyone who calls (outside of MBM) so the Asthma UK nurse team would not require additional training.

Motivating and reassuring support messages will be sent by email to all patients in the intervention group every 2 to 4 weeks; these will encourage and reinforce patient adherence to lifestyle changes (where applicable).

6.2 USUAL CARE

All patients will be able to access the Asthma UK information leaflet following randomisation. Patients within the usual care condition will be informed that they should simply follow their usual instructions for asthma management. After the patients have completed the 12-month follow-up they will be given access to the intervention, in line with recommendations from PPI representatives.

7. ADVERSE EVENTS AND REPORTING

7.1 DEFINITIONS

Adverse Event (AE): any untoward medical occurrence in a participant or clinical study participant which does not necessarily have a causal relationship with study treatment or participation.

Serious Adverse Event (SAE) or Serious Adverse Reaction: any untoward medical occurrence or effect that at any dose:

- Results in death
- Is life-threatening – refers to an event in which the participant was at risk of death at the time of the event; it does not refer to an event which hypothetically might have caused death if it were more severe
- Requires hospitalisation, or prolongation of existing hospitalisation
- Results in persistent or significant disability or incapacity
- Is a congenital anomaly or birth defect
- Other important medical events***.

*'life-threatening' in the definition of 'serious' refers to an event in which the patient was at risk of death at the time of the event; it does not refer to an event which hypothetically might have caused death if it were more severe.
**Hospitalisation is defined as an inpatient admission, regardless of length of stay, even if the hospitalisation is a precautionary measure for continued observation. Hospitalisations for a pre-existing condition, including elective procedures that have not worsened, do not constitute an SAE.
***Other important medical events that may not result in death, be life-threatening, or require hospitalisation may be considered a serious adverse event/experience when, based upon appropriate medical judgment, they may jeopardise the participant and may require medical or surgical intervention to prevent one of the outcomes listed in this definition.

Note: It is the responsibility of the PI or designated to grade an event as 'not serious' (AE) or 'serious' (SAE).

Ainsworth B, *et al. BMJ Open* 2019; 9:e032465. doi: 10.1136/bmjopen-2019-032465

7.2 CAUSALITY

The assignment of the causality to trial procedures of any serious event should be made by the investigator responsible for the care of the participant using the definitions in the table below.

If any doubt about the causality exists the local investigator should inform the trial coordinator who will notify the Chief Investigator. Other clinicians may be asked for advice in these cases.

In the case of discrepant views on causality between the investigator and others, all parties will discuss the case. In the event that no agreement is made, the Ethics Committee will be informed of both points of view.

Relationship	Description
**Unrelated**	There is no evidence of any causal relationship
**Unlikely**	There is little evidence to suggest there is a causal relationship (e.g. the event did not occur within a reasonable time after administration of the trial medication). There is another reasonable explanation for the event (e.g. the participant's clinical condition, other concomitant treatment).
**Possible**	There is some evidence to suggest a causal relationship (e.g. because the event occurs within a reasonable time after administration of the trial medication). However, the influence of other factors may have contributed to the event (e.g. the participant's clinical condition, other concomitant treatments).
**Probable**	There is evidence to suggest a causal relationship and the influence of other factors is unlikely.
**Definitely**	There is clear evidence to suggest a causal relationship and other possible contributing factors can be ruled out.

7.3 REPORTING PROCEDURES

All adverse events should be reported. Depending on the nature of the event the reporting procedures below should be followed. Any questions concerning adverse event reporting should be directed to the SCTU in the first instance.

7.3.1. Pre-existing Conditions

A pre-existing condition should not be reported as an AE unless the condition worsens by at least one CTCAE grade during the trial. The condition, however, must be reported in the pre-treatment section of the CRF, if symptomatic at the time of entry, or under concurrent medical conditions if asymptomatic.

7.3.2. Non serious AEs

All adverse events that may be related to the study will be recorded in the relevant case report form and Adverse Event form and sent to the SCTU within one month of the form being due. As adults on average see their GP approximately 5 times per year for a variety of routine and unscheduled appointments (e.g. for medication review, self-limiting minor illnesses and long-

Ainsworth B, *et al. BMJ Open* 2019; 9:e032465. doi: 10.1136/bmjopen-2019-032465

term conditions unrelated to asthma), many medical encounters are of no relevance to the study. Events that will be recorded include any judged by the study nurse to be possibly related to the study. In particular, all medical encounters related to the following medical areas or symptoms will be recorded in the Adverse Events form:

Psychological morbidity: any events relating to anxiety, depression or mood disorders
Respiratory morbidity: any events relating to breathing or chest symptoms
Musculoskeletal, Abdominal and chest pain: any events relating to pain in these systems unless known to be associated with an unrelated pre-existing condition.

The study nurses are advised to record any event for which there is uncertainty as to whether it is study related or not, and to discuss with the local PI or CI.

7.3.3 Serious AEs

All SAEs (including those that are expected and related) will be reported within 24 hours of the local site becoming aware of the event. The SAE form asks for nature of event, date of onset, severity, corrective therapies given or action taken, outcome and relatedness (i.e. unrelated, unlikely, possible, probably, definitely). The responsible centre Principal Investigator will assign the relatedness and expectedness of the event. Additional information will be provided as soon as possible if the event has not resolved at the time of reporting.

A flowchart is given below to illustrate reporting procedures:

- GPs or nurses will be asked to notify us via an SAE form if a participant experiences any SAEs.
- The Sponsor and main Research Ethics Committee (REC) will be informed of all related SAEs occurring during the trial according to the following timelines, where day zero is defined as the date the SAE form is initially received:
- Events which are fatal or life-threatening will be reported no later than 7 calendar days after the sponsor is first aware of the reaction. Any additional relevant information must be reported within a further 8 calendar days.
- Events that are non-fatal or non life-threatening will be reported within 15 calendar days of the sponsor first becoming aware of the reaction.
- All Investigators will be informed of all related SAEs occurring throughout the trial. Local Investigators should report any SAEs as required by their Local Research Committee and/or Research and Development Office.

7.3.4 Follow Up and Post-study Serious Adverse Events

The reporting requirement for SAEs affecting participants applies for all events occurring up to the end of the last treatment. All unresolved adverse events should be followed by the local investigator until resolved, the participant is lost to follow-up, or the adverse event is otherwise explained. At the last scheduled visit, the investigator should instruct each participant to report any subsequent event(s) that the participant, or the participant's general practitioner, believes might reasonably be related to participation in this study. The investigator should notify the Centre for Applications of Health Psychology of any death or adverse event occurring at any time after a participant has discontinued or terminated study participation that may reasonably be related to this study.

Ainsworth B, *et al. BMJ Open* 2019; 9:e032465. doi: 10.1136/bmjopen-2019-032465

8. ASSESSMENT AND FOLLOW-UP OF PARTICIPANTS

Measures will be administered for all participants at baseline, 3 and 12 months, unless otherwise stated (see Schedule of Observations and Procedures). Patient-reported outcome measures will be completed online. Non-respondents will receive two email reminders followed by a paper-based copy accompanied by a final telephone follow-up. All website usage (MBM measures) will be recorded automatically in Lifeguide.

An independent research nurse, blind to study allocation will complete the follow-up assessments. All patients (including withdrawn participants who have consented to follow-up appointments) will follow the procedure outlined below:

- Approximately three weeks before the follow-up appointment is due, the patient will be sent a letter by the research team encouraging them to arrange an appointment with the research nurse. Appointments may be scheduled to take place in the patients' home or usual GP practice.
- If the patient does not contact the research team to arrange an appointment within two weeks of the letter being sent the patient will be followed up by telephone.
- The research team or research nurse will send confirmation of the follow-up appointment to the patient by telephone, text, email or letter (dependent on patient preference).
- If the patient indicates that they would not be willing to complete a follow-up, no further contact will be made with the patient regarding the follow-up appointment.

8.1 DEFINITION OF END OF TRIAL

The end of the trial will be defined as date of entry of final data into database.

9. QUANTITATIVE EVALUATION

9.1 SAMPLE SIZE

A minimum of 40 participants from two centres will be recruited to each condition during the study.

9.2 RANDOMISATION

Participants will be randomised in a 1:1 ratio to receive either usual care (control) or MBM programme with optional support (intervention). The randomisation will be stratified block randomisation by the average value of AQLQ scores in the BREATHE Trial (Thomas et al).

Remote allocation will maintain allocation concealment from both the participant and the research nurse prior to allocation, however the outcome assessor will be blinded to participant allocation.

Ainsworth B, *et al. BMJ Open* 2019; 9:e032465. doi: 10.1136/bmjopen-2019-032465

9.3 STATISTICAL PLAN INCLUDING INTERIM ANALYSIS

A detailed statistical plan will be developed prior to data lock. No interim analysis is planned. Data and all appropriate documentation will be stored for a minimum of 10 years after the completion of the trial, including the follow-up period, in a secure location at Southampton University.

Descriptive statistics of outcome variables will be calculated in order to inform a future randomized controlled trial, including examining levels of missing data, attrition rate, retention and completion. Statistical comparisons between and within intervention and control groups will be performed to estimate variability in primary and secondary outcomes within a linear regression framework. Further exploratory analysis of patient engagement, intervention usage and possible mediators of behaviour change will be performed.

10. QUALITATIVE EVALUATION

10.1 Qualitative Evaluation

The qualitative study will be undertaken both alongside participation and after participants finish using MBM. Qualitative interviews will seek to provide an in-depth understanding of the perspective of patients, to inform intervention and trial acceptability and to generate hypotheses about intervention mechanisms of action that can be tested in a larger trial.

Participants and sampling

Patients will be asked whether they give permission to take part in an interview at a later point in the study. Purposive sampling will be used to select patients from the intervention group to allow for a wide range of views and experiences of the MBM programme. It is anticipated that 20 patients will be interviewed during and following the study, and patients will be selected for interviews until saturation is reached.

Interviews and qualitative analysis

Factors that may facilitate or diminish the acceptability of the MBM programme, and adherence to implementation will be explored across patient and health care professional interviews. Interviews will be conducted from after the 3-month assessment. Open-ended questions will be used to elicit user perspectives and experiences of the intervention, allowing participants to freely describe their experiences and views in their own way and to focus on whatever is most salient to them.

Interviews will be audio-recorded and fully transcribed. The findings will be used to inform any modifications needed to the MBM programme or the trial procedures for a potential future full RCT.

The transcriptions will be anonymised (identifiable data removed) and participants' transcripts will be given participant numbers so that they can be easily discussed between team members whilst protecting participants' identities. To ensure that we remain open to and grounded in users' perspectives we will carry out inductive thematic analysis[22] of all textual data,

triangulated where appropriate with quantitative self-report measures and web usage data, and constant comparison and discussion among team members to reach inter-rater agreement on themes and interpretations.

11. REGULATORY ISSUES

11.1 CLINICAL TRIAL AUTHORISATION

This trial does NOT involve the testing of any Investigational Medicinal Products (IMPs) therefore approval from the Medicines and Healthcare products Regulatory Agency is not required.

11.2 ETHICS APPROVAL

The trial protocol will be submitted to a Research Ethics Committee (REC) recognised by the United Kingdom Ethics Committee Authority (UKECA) for review and approval. A favourable opinion must be obtained before commencement of any trial procedures (including recruitment of patients) occurs

All substantial amendments must be approved by the REC responsible for the trial, in additional to approval by NHS R&D. Minor amendments will not require prior approval by the REC.

If the trial is stopped prematurely, it will not be recommended without reference to the REC responsible for the trial.

The outcome of the trial will be reported to the responsible REC within 90 days of completion of the last patient's final trial procedures. In the event of the trial being prematurely terminated a report will be submitted to the REC within 15 days. A summary of the Trial Report will be submitted to the responsible REC within one year of completion of the last participant's final study procedures.

The investigator must ensure that participant's anonymity will be maintained and that their identities are protected from unauthorised parties. On CRFs participants will not be identified by their names, but by an identification code.

11. 3 CONFIDENTIALITY

Initial practice database searchers will be conducted on practice computer systems and subsequent study invitations will be the responsibility of the practice, to maintain the confidentiality of potential participants. Participants' identification data will be required for the registration process, which will be completed with the support provider at the baseline screening appointment.

Ainsworth B, *et al. BMJ Open* 2019; 9:e032465. doi: 10.1136/bmjopen-2019-032465

Participants will be informed that the research team will have access to their study data, and that personal information such as their name, telephone address and email address will be stored and used by the research team to stay in touch with them throughout the study.

The primary data outcomes measured by the support provider (such as asthma quality of life and current medication) will be stored on an independent, secure server. All other data (secondary self-report measures and intervention data) will be stored on dedicated secure spaces behind a firewall on password protected computer located in secure university buildings at the University of Southampton. The data is backed up daily. Confidentiality of all data entered into the DI will be maintained by following best practice in NHS IT protection and data security systems (e.g. regarding use of https, storing data behind the university firewall etc). Access to the website will be via username and password. The use of strong passwords will be enforced. The communication between the person entering data through a web browser and the server will be through a secure internet connection (HTTPS). The research team will adhere to the Data Protection Act 1998.

Digital records will be stored on a university computer in a password protected file. All data will be linked with the participant's study ID which does not include any personal or identifiable information, such as name or D.O.B. Study IDs will be linked with patient's names, email addresses telephone numbers in a separate, securely stored file.

At the end of the study, data will be anonymised and stored on a password protected computer located in secure university buildings and appropriately backed up. Confidentiality of all data entered into the DI will be maintained by following best practice in NHS IT protection and data security systems.

11.4 INDEMNITY

The sponsor of the trial is University of Southampton. University of Southampton insurance will apply. The lead authors of the protocol are the Chief Investigator (Prof Lucy Yardley), and Prof Mike Thomas, who are both university employees and as such will be covered by the University insurance.

11.5 SPONSOR

University of Southampton is acting as the sponsor for this trial. The My Breathing Matters coordinating team has been delegated duties by the Sponsor relating to: submissions to regulatory authorities and GCP.

11.6 FUNDING

NIHR PGfAR are funding this trial (RP-PG-1211-20001).

11.7 DEVIATIONS AND SERIOUS BREACHES

Any trial protocol deviations/violations and breaches of Good Clinical Practice occurring at sites should be reported to the MBM trial coordinator and the local R&D Office immediately.

The MBM trial coordinator will then advise of and/or undertake any corrective and preventative actions as required.

All serious protocol deviations/violations and serious breaches of Good Clinical Practice and /or the trial protocol will immediately be reported to the regulatory authorities and other organisations, as required in the Medicines for Human Use (Clinical Trials) Regulations 2004, as amended.

11.8 AUDITS AND INSPECTIONS

The trial may be participant to inspection and audit by University of Southampton, under their remit as sponsor, the trial coordinating centre as the Sponsor's delegate and other regulatory bodies to ensure adherence to ICH GCP, Research Governance Framework for Health and Social Care, applicable contracts/agreements and national regulations.

12. TRIAL MANAGEMENT

The Trial Management Group (TMG) is responsible for overseeing progress of the trial. The day-to-day management of the trial will be co-ordinated through the trial coordinating centre and oversight will be maintained by the Trial Steering Committee, with an experienced independent chair and representation of patient representatives, Asthma UK and Blood Pressure UK.

The Steering Committee will meet twice a year throughout the programme to provide strategic guidance and independent monitoring of progress and professional conduct. We will encourage in person attendance at all these meetings where possible, but will also provide for attendance by teleconference when necessary, and will circulate papers and minutes before and after meetings for communication with those who cannot attend for any reason.

13. PUBLICATION POLICY

All publications and presentations relating to the trial will be authorised by the Trial Management Group and will follow an agreed publication policy. Dissemination of our work will be via multiple pathways:
a) to the scientific community through presentation at national & international conferences and regular publication in highly cited and open access peer reviewed journals.
b) to clinical and academic colleagues via professional societies: links with the following societies will be exploited to raise the profile of this work: Royal College of GPs and Physicians, Society of Behavioural Medicine, British Sociological Society Medical Sub-group.
c) to patients via patient groups: We will work with Asthma UK to disseminate our results.
d) to participants: All participants will be sent an accessible summary of the findings from the study that they took part in within six months of study completion.
e) to relevant NHS organisations and healthcare providers (e.g. Clinical Commissioning Groups, NHS Choices, UCL partners).

Ainsworth B, *et al. BMJ Open* 2019; 9:e032465. doi: 10.1136/bmjopen-2019-032465

f) to the public via local and national media: we will use regular press releases linked to dissemination events to ensure a high level awareness of our work in the media.
g) to all stakeholders via a dedicated website and through interactive workshops (with health professionals, patient groups, IT providers, commissioners, policy-makers, researchers).

14. REFERENCES

1. Department of Health. An outcomes strategy for people with chronic obstructive pulmonary disease (COPD) and asthma in England. 2011.
2. Demoly P, Gueron B, Annunziata K, Adamek L, Walters RD. Update on asthma control in five European countries: results of a 2008 survey. *European Respiratory Review* 2010;19:150-157.
3. Gibson PG, Powell H, Coughlan J et al. Self-management education and regular practitioner review for adults with asthma. *Cochrane Database of Systematic Reviews* 2002;3.
4. British Thoracic Society / Scottish Intercollegiate Guideline Network. British Guideline on the Management of Asthma. 2012. 22-8-0012.
5. Adams R, Smith B, Ruffin R. Factors associated with hospital admissions and repeat emergency dept visits for adults with asthma . *Thorax* 2000;55:566-573.
6. Asthma UK. National Asthma Panel 2010. 2010. London, Asthma UK.
7. Thomas M, Price D. Impact of comorbidities on asthma. *Expert Reviews in Clinical Immunology* 2008;4:731-742.
8. Ogedegbe G. Barriers to optimal hypertension control. *Journal of Clinical Hypertension* 2008;10:644-666.
9. Guthrie B, Inkster M, Fahey T. Tackling therapeutic inertia: role of treatment data in quality indicators. *Br Med J* 2007;335:542-544.
10. Ogedegbe G, Schoenthaler A. A systematic review of the effects of home blood pressure monitoring on medication adherence. *Journal of Clinical Hypertension* 2006;8:174-180.
11. Bosworth HB, Olsen MK, Grubber JM et al. Two Self-management Interventions to Improve Hypertension Control: A Randomized Trial. *Annals of Internal Medicine* 2009;151:687-695.
12. Agarwal R, Bills JE, Hecht TJW, Light RP. Role of home blood pressure monitoring in overcoming therapeutic inertia and improving hypertension control: a systematic review and meta-analysis. *Hypertension* 2011;57:29-38.
13. Abudagga A, Resnick HE, Alwan M. Impact of blood pressure monitoring on hypertension outcomes: a literature review. *Telemedicine and e-Health* 2010;16:830-837.
14. McLean G, Murray E, Band R et al. Interactive digital interventions to promote self-management in adults with asthma: systematic review and meta-analysis. *BMC Pulmonary Medicine* 2016:16:83.
15. Rasmussen LMPK, Nolte H, Backer V. Internet-based monitoring of asthma: A long-term, randomized clinical study of 300 asthmatic subjects. *Journal of Allergy and Clinical Immunology* 2005;115:1137-1142.
16. Morrison D, Wyke S, Saunderson K et al. Findings from a pilot randomised trial of an Asthma Internet Self-management Intervention *BMJ Open* 2016:6:e009254.
17. Morrison D, Mair F, Chaudhuri R et al. Details of development of the resource for adults with asthma in the RAISIN (randomized trial of an asthma internet self-management intervention) study. *BMC Medical Informatics & Decision Making* 2015; 15:57.
18. van der Meer V, Bakker MJ, van den Hout WB et al. Internet-based self-management plus education compared with usual care in asthma: A randomized trial. *Annals of Internal Medicine* 2009;151:110-120.
19. van der Meer V, van den Hout WB, Bakker MJ et al. Cost-Effectiveness of Internet-Based Self-Management Compared with Usual Care in Asthma. *PLoS ONE* 2011;6:e27108.

Ainsworth B, *et al*. *BMJ Open* 2019; 9:e032465. doi: 10.1136/bmjopen-2019-032465

20. Yardley L, Ainsworth B, Arden Close E et al. The person-based approach to enhanceing the acceptability and feasibility of interventions, *Pilot & Feasibility Studies, 2015 : 1.*
21. Al-Janabi H, Flynn T, Coast J. (2012) Development of a self-report measure of capability wellbeing for adults: the ICECAP-A. *Quality of Life Research* 21: 167-176
22. Joffe H, Yardley L. Content and thematic analysis. In: Marks D, Yardley L, eds. *Research Methods for Clinical and Health Psychology*. London: Sage; 2004;56-68.

15. Trial Timeline

	2016		2017												2018		
	Nov	Dec	Jan	Feb	Mar	Apr	May	Jun	Jul	Aug	Sep	Oct	Nov	Dec	Jan	Feb	Mar
Practice recruitment																	
Study Material Preparation																	
Website live date																	
Patient recruitment to trial																	
Feasibility & Acceptability evaluations																	
3 month follow-ups																	
12 month follow ups																	
Qualitative process interviews																	

Ainsworth B, *et al. BMJ Open* 2019; 9:e032465. doi: 10.1136/bmjopen-2019-032465
