# Supplementary material for: Feasibility trial of a digital self-management intervention ‘My Breathing Matters’ to improve asthma-related quality of life for UK primary care patients with asthma
Source: BMJ Open. 2019 Nov 12;9(11):e032465. doi: 10.1136/bmjopen-2019-032465 (PMC6858238; doi:10.1136/bmjopen-2019-032465)
Supplement: Supplementary data [file bmjopen-2019-032465supp002.pdf]

Appendix 2. Time and costs Questionnaire summary

	Demographic characteristic	
	Intervention	Usual Care
In the last 12 months, have you signed up to any new programmes (e.g. gym membership, taken up a new activity like yoga/meditation) that might help your health?	Lots of new programmes (2%)<br>Some new programmes (27%)<br>No new programmes (50%)<br>Missing (21%)	Lots of new programmes (0%)<br>Some new programmes (36%)<br>No new programmes (69%)<br>Missing (4%)
Have any new programmes that you have signed up to (e.g. gym membership, taken up a new activity) cost you any money that you might have spent elsewhere?	Yes (14%)<br>No (64%)<br>Missing (23%)	Yes (21%)<br>No (68%)<br>Missing (11%)
Total amount spent on other programmes (M, SD)	363.75 (320.30)	336.25 (379.81)
