# Supplementary material for: Feasibility trial of a digital self-management intervention ‘My Breathing Matters’ to improve asthma-related quality of life for UK primary care patients with asthma
Source: BMJ Open. 2019 Nov 12;9(11):e032465. doi: 10.1136/bmjopen-2019-032465 (PMC6858238; doi:10.1136/bmjopen-2019-032465)
Supplement: Author's manuscript [file bmjopen-2019-032465.draft_revisions.pdf]

BMJ Open

BMJ Open is committed to open peer review. As part of this commitment we make the peer review history of every article we publish publicly available.

When an article is published we post the peer reviewers' comments and the authors' responses online. We also post the versions of the paper that were used during peer review. These are the versions that the peer review comments apply to.

The versions of the paper that follow are the versions that were submitted during the peer review process. They are not the versions of record or the final published versions. They should not be cited or distributed as the published version of this manuscript.

BMJ Open is an open access journal and the full, final, typeset and author-corrected version of record of the manuscript is available on our site with no access controls, subscription charges or pay-per-view fees (http://bmjopen.bmj.com).

If you have any questions on BMJ Open's open peer review process please email
info.bmjopen@bmj.com

BMJ Open

A feasibility trial of a digital self-management intervention 'My Breathing Matters' to improve asthma-related quality of life for primary care patients with asthma.

Journal:	*BMJ Open*
Manuscript ID	bmjopen-2019-032465
Article Type:	Research
Date Submitted by the Author:	27-Jun-2019
Complete List of Authors:	Ainsworth, Ben; University of Bath, Psychology; University Hospital Southampton NHS Foundation Trust, NIHR Respiratory Biomedical Research Unit<br>Greenwell, Kate; University of Southampton, Psychology<br>Stuart, Beth; University of Southampton, Primary Care and Population Sciences, Faculty of Medicine<br>Raftery, James; University of Southampton, Primary Care and Population Sciences, Faculty of Medicine<br>Mair, Frances; University of Glasgow, Institute of Health and Wellbeing<br>Bruton, Anne; University of Southampton, School of Health Sciences, Faculty of Environmental and Life Sciences<br>Yardley, Lucy; University of Southampton, Psychology; University of Bristol, School of Psychological Sciences<br>Thomas, Mike; University of Southampton, Primary Care and Population Sciences, Faculty of Medicine
Keywords:	Asthma < THORACIC MEDICINE, PRIMARY CARE, Digital Medicine, Self-management, Breathing Retraining, Quality of Life

SCHOLARONE™
Manuscripts

BMJ Open

A feasibility trial of a digital self-management intervention 'My Breathing Matters' to improve asthma-related quality of life for primary care patients with asthma.

Ben Ainsworth[1,2,*]

Kate Greenwell[3]

Beth Stuart[4]

James Raftery[4]

Frances S Mair[5]

Anne Bruton[6]

Lucy Yardley[3,7]

Mike Thomas[4]

*: corresponding author: b.ainsworth@bath.ac.uk

1: Department of Psychology, Faculty of Humanities and Social Sciences, University of Bath, UK.

2: Respiratory Biomedical Research Unit, University Hospitals Southampton NHS Foundation Trust, Southampton, Hampshire, UK.

3: Centre for Clinical and Community Applications of Health Psychology, School of Psychology, Faculty of Environmental and Life Sciences, University of Southampton, UK.

4. Primary Care and Population Sciences, Faculty of Medicine, University of Southampton, UK.

5. Institute of Health and Wellbeing, University of Glasgow, UK

6. School of Health Sciences, Faculty of Environmental and Life Sciences, University of Southampton, UK

7. School of Psychological Science, University of Bristol, UK.

Keywords: asthma, self-management, quality of life, primary care, breathing retraining, digital

For peer review only - http://bmjopen.bmj.com/site/about/guidelines.xhtml

Abstract

Objective: To assess the feasibility of a randomised controlled trial (RCT) and acceptability of an asthma self-management digital intervention to improve asthma-specific quality of life in comparison with usual care.

Design and setting: A two arm feasibility RCT conducted across 7 general practices in Wessex, UK.

Participants: Primary care patients with asthma aged 18 years and over, with impaired asthma-specific quality of life and access to the internet.

Interventions: 'My Breathing Matters' (MBM) is a digital asthma self-management intervention designed using theory, evidence and person-based approaches to provide tailored support for both pharmacological and non-pharmacological management of asthma symptoms.

Outcomes: The primary outcome was the feasibility of the trial design, including recruitment, adherence and retention at follow-up (3 and 12 month). Secondary outcomes were the feasibility and effect sizes of specific trial measures including asthma-specific quality of life and asthma control.

Results: Primary outcomes: 88 patients were recruited (target 80). At 3 month follow-up, 2 patients withdrew and 6 did not complete outcome measures. At 12 month, 2 withdrew and 4 did not complete outcome measures. 36/44 patients in the intervention group engaged with MBM (median of 4 logins, range 0-25). Consistent trends were observed to improvements in asthma-related patient reported outcome measures.

Conclusions: This study demonstrated the feasibility and acceptability of a definitive randomised controlled trial to determine the clinical and cost-effectiveness of a digital asthma self-management intervention.

Trial registration number: ISRCTN15698435.

For peer review only - http://bmjopen.bmj.com/site/about/guidelines.xhtml

BMJ Open

Strengths and Limitations of the Study (Summary: up to 5 bullet points)

1. This pragmatic randomised controlled feasibility trial examined ‘My Breathing Matters’ (MBM), a digital asthma self-management intervention that supported both pharmacological and non-pharmacological management of asthma symptoms.
2. MBM was developed using theory, evidence and person-based approaches, and compared to standardised usual care (a booklet) with successful blinding and randomisation.
3. Not all patients engaged with the intervention, and although numeric improvements in patient reported asthma outcomes were larger in the active arm, improvements were observed in both arms and the study was not powered to show statistical significance.

For peer review only

For peer review only - http://bmjopen.bmj.com/site/about/guidelines.xhtml

BMJ Open

Introduction

Asthma prevalence in the UK is amongst the highest in the world at nearly 6% of the UK adult population, comprising 5.4 million people, with most managed in primary care. Although hospital admission and mortality rates for asthma improved from 1970 to 2000, these improvements have since stalled[1]. Surveys of asthma symptoms and health status impairment show that sub-optimal control is common and that the majority of people with asthma in the UK frequently experience potentially avoidable symptoms and quality of life impairment[2].

Proactive self-management of asthma has been convincingly shown to improve clinical outcomes and have been advocated in guidelines for 25 year[3]. Guidelines are not always well implemented[4] and consequently some people with asthma do not receive evidence-based interventions which are known to impact positively on outcomes. Recent large-scale systematic reviews demonstrated that supported asthma self-management can reduce healthcare utilisation and increase asthma control, without increasing healthcare costs[5,6]. For example, people with asthma without a management plan are four times more likely to have an asthma attack needing emergency care in hospital, yet only 44% of people with asthma in the UK report having a self-management plan[7]. Self-management recommendations for asthma have also encompassed non-pharmacological strategies to improve control. These include lifestyle interventions, such as smoking cessation, allergen avoidance, weight reduction in those with obesity, and breathing retraining interventions[8].

Digital interventions (DIs) are increasingly recognised as a possible approach to achieve the aims of supporting chronic diseases such as asthma. DIs can be convenient, easily accessed and may provide cost-effective tools by automating routine aspects of patient education, monitoring and support[9]. There is accumulating evidence that DIs are feasible and may be effective in the context of asthma. The SMASHING trial compared usual care with web-based educational resources, self-monitoring and automated feedback on medication titration, plus some group and email nurse support for patients with asthma. After 12 months the intervention group had

For peer review only - http://bmjopen.bmj.com/site/about/guidelines.xhtml

BMJ Open

better quality of life and lung function and more symptom-free days, at no extra cost[10]. The RAISIN pilot trial indicated that self-management interventions that included non-pharmacological (behavioural and psychological) components could be effective at improving quality of life and asthma control, with improvements to 'reach' and response rate, by catering to patients with mild asthma but impaired quality of life[11]. A recent systematic review and meta-analysis indicated that while self-management DIs may be able to improve asthma control and reduce asthma-related quality of life impairment, larger confirmatory trials are required[12].

In the current randomised controlled feasibility trial, we developed and evaluated a digital self-management intervention, that incorporated pharmacological and non-pharmacological self-management support for adults in primary care with impaired asthma-specific quality of life ('My Breathing Matters'; MBM), using evidence, theory and person-based approaches[13] and in line with Medical Research Council (MRC) guidance for developing and evaluating complex interventions[14].

Aim

The aim of the MBM study was to assess the feasibility of a trial to evaluate a digital intervention in primary care to improve clinical outcomes (such as quality of life, health resource use, lung function) of people with asthma, in comparison to usual care (with provision of standard patient information materials produced by the charity Asthma UK).

Research Objectives

1. To assess feasibility of trial procedures including recruitment strategy, eligibility criteria, consent, withdrawal, randomisation and blinding.
2. To assess feasibility of the MBM digital intervention including usage and engagement.
3. To assess feasibility of data analysis, including data collection, data quality and management of trial data across trial endpoint measures to inform sample size calculations for a larger phase 3 randomised controlled trial.

For peer review only - http://bmjopen.bmj.com/site/about/guidelines.xhtml

For peer review only

BMJ Open

Method

Design

We conducted a pragmatic feasibility RCT of the MBM digital intervention in primary care.

Setting

Eligible participants were identified from seven general practices from the Wessex, UK primary care research network to facilitate recruitment of people with varied socio-economic status. Practices were purposively selected to be both rural (N = 4) and urban (N = 3), with a spread across socio-economic deprivation (mean practice deprivation index 20.60% (SD 10.5); practice socio-economic deprivation deciles = 2, 4, 4, 5, 8, 10, 10, in which lower deciles indicate more deprivation[15]).

Participants

Patients were included in the trial if they were aged 18 years or more, had physician-diagnosed asthma in their medical record, had received one or more anti-asthma medication prescription in the previous 12 months, had impaired asthma-related health status (Asthma Quality of Life Questionnaire score of less than 5.5 as assessing using a self-completed postal questionnaire), provided informed consent, were able to understand English and had access to the internet.

They were excluded from the trial if 1) their GP considered it inappropriate for them to take part (such as having an additional terminal condition), 2) they were attending a secondary care asthma clinic, or they were receiving either maintenance oral corticosteroids or injected biological treatments to control their asthma, 3) they were diagnosed with COPD, 4) they had a household member already enrolled on the study, or 5) they were judged by the research nurse to have 'unstable asthma' according to clinical assessment and spirometry data at the baseline assessment (in which case they were referred back to their GP), or were diagnosed with 'difficult asthma' defined by British Thoracic Society (BTS).

Recruitment

Electronic searches of the computerised primary care medical record were conducted, and

For peer review only - http://bmjopen.bmj.com/site/about/guidelines.xhtml

records screened by GP to remove ineligible participants. Invitation letters, study information sheets, consent forms screening questionnaires (Mini Asthma Quality of Life Scale; AQLQ[16]) and freepost return envelopes were posted to participants who returned them if they were interested in taking part. Patients who met screening criteria (AQLQ score of less than 5.5) were contacted by research team staff and attended a baseline appointment at their practice with a trained research nurse. Recruitment began in March 2017 and was completed in August 2017.

Sample size

The target for this trial was to recruit 80 patients overall (40 per arm), in order to assess primary feasibility outcomes and to assess intervention engagement and acceptability.

Randomisation and Blinding

After completing outcome measures at their baseline appointment participants were randomised (block randomisation stratified by an average primary care AQLQ score [4.3] taken from a previous trial using the same inclusion criteria[8]). Information packs were given to participants after randomisation with instructions on signing up to MBM (if randomised to intervention group) or just usual care materials (if randomised to control). Research nurses conducting baseline appointments were blinded throughout the study until the final questionnaire which was only delivered to the intervention group.

Interventions described according to Tidier checklist[17]

Intervention Group: Usual Care with MBM and Asthma UK booklet.

Patients in the intervention group continued to receive usual care but were also given a code that allowed free unlimited access to MBM. MBM is a digital asthma self-management intervention that supports asthma self-management using both pharmacological and non-pharmacological approaches, developed using the LifeGuide Software[18]. A demonstration version of the intervention is available here: http://www.mybreathingmatters.co.uk. After signing up and completing quality-of-life related self-monitoring questions, patients were

For peer review only - http://bmjopen.bmj.com/site/about/guidelines.xhtml

For peer review only

offered tailored advice that directed them towards specific pharmacological or non-pharmacological sections of the online intervention. The pharmacological section provided information on different medication classes and inhalers, the use of personalised asthma action plans (PAAPs), encouraged medication adherence, and gave information to facilitate and inform an effective asthma review with their GP. Pharmacological content was initially based on 'Living Well with Asthma', an asthma self-management intervention that previously demonstrated feasibility for self-management[11,19] and was developed in collaboration with people with asthma and with input from Asthma UK (a national asthma charity). This section was designed to answer common concerns about medication, incorporating a strategy described as 'the 4-week medication challenge' that encouraged participants to realise the benefits of adherence to regular medication, by self-monitoring their symptoms during 4 weeks of continuous inhaler use. The non-pharmacological support included sections on a number of strategies to improve asthma control, such as Breathing Retraining, stress reduction, and additional healthy lifestyle resources (physical activity, weight reduction, hand hygiene and smoking cessation). Optional nurse support was available by Asthma UK who provide a dedicated nurse helpline that was advertised through the intervention.

The intervention was developed using the person-based approach[20] which places patients at the heart of the development process. Evidence from primary mixed methods research (such as [19]) and qualitative and quantitative reviews was used to develop guiding principles. A prototype intervention was piloted using 'think aloud' interview studies in which patients with asthma used prototype versions of the MBM website and provided feedback on intervention acceptability and feasibility as they used it. In 46 interviews with 30 patients, the intervention was iteratively modified and updated to address patient feedback until participants indicated no further modification was required, confirming the intervention was as acceptable and engaging as possible (for more details on this process see [21]).

Due to the digital nature of the intervention, participants could engage with components of the intervention as much or as little as they wished. Tailored advice was offered according to

For peer review only - http://bmjopen.bmj.com/site/about/guidelines.xhtml

BMJ Open

participants' preference to find out more about pharmacological or non-pharmacological self-management techniques (patients selected a check box option of "I'd like to find out more about how my asthma could be helped by i) making the most of my asthma medicine, or ii) 'non-medicine' ways to help my breathing."), with automated reminders whenever patients had not accessed the intervention for several weeks, or when content was made available that they had not previously seen. The intervention was not modified during the study.

Intervention usage was monitored through digital usage metrics (reported below).Non-engagement with the website was not addressed, in line with the pragmatic nature of the feasibility study. Participants were sent one email and received one phone call in which they were offered technical support if they had not logged onto the intervention at all for one month following their baseline appointment.

Control Group: Usual Care with Asthma UK Booklet

To provide 'good quality' usual care to participants allocated to this arm, as well as usual care from their practice, participants were given an Asthma UK booklet 'Live Well with Asthma' at their baseline appointment. The booklet was created by a multidisciplinary team and expert patients, and aimed to provide essential information and advice to enable effective self-management to occur. It is available to anyone via the Asthma UK website[22]. The booklet was provided in hard copy and provided information about asthma symptoms and triggers, medication adherence and usage techniques, PAAPs and support from families. The booklet also advertised the Asthma UK support line. Booklet usage was not monitored.

Outcome Measures

In line with objectives, trial outcomes are reported below as 1) feasibility outcome measures, 2) intervention usage outcome measures, and 3) trial endpoint measures to inform a larger trial.

For peer review only - http://bmjopen.bmj.com/site/about/guidelines.xhtml

BMJ Open

Feasibility outcome measures: Primary outcomes for the trial were descriptive, examining trial design and intervention feasibility and acceptability. These outcomes included patient recruitment, patient withdrawals and follow-up retention.

Internet usage and engagement measures: Usage of the intervention included access to specific intervention components and frequency of engagement with individual components. These data were collected using the LifeGuide software.

Endpoint Measures: It is envisaged that the likely primary outcome measure or measures in a full trial would include validated asthma-specific patient reported outcome measures evaluating symptom control and quality of life, with additional secondary outcomes measuring health resource use, psychological measures and a health economic economic analysis. Data to generate hypotheses (and perform sample size calculations with which to test them) were collected in the following trial endpoints at baseline, 3 month and 12 month:

- Asthma-specific quality of life. Measured using the Mini Asthma Quality of Life Questionnaire (AQLQ[16]), a 32-item 7-point scale in which higher scores represent higher quality of life.
- Asthma control. Measured using the Asthma Control Questionnaire (ACQ[23]) , a 7-item 7-point scale in which higher scores indicate worse asthma control.
- Health-related quality of life. Measured using the EQ-5D-5L[24] in which participants select their functioning level across five dimensions (immobility, self-care, usual activities, pain and discomfort, and anxiety and depression) on a five-point scale, with higher scores indicating greater problems with functioning.
- Health-related capability. Measured using the Icepop Capability measure for adults (ICECAP-A[25]), in which participants select their capability across five dimensions (stability, enjoyment, achievement, attachment and autonomy) on a four-point scale, with higher scores indicating better capability.

For peer review only - http://bmjopen.bmj.com/site/about/guidelines.xhtml

BMJ Open

- Anxiety and depression. Measured using the Hospital Anxiety and Depression Scale (HADS[26]), a 12-item questionnaire in which higher scores on depression and anxiety subscales indicate higher anxiety across two subscales (anxiety and depression).
- Enablement, measured using a modified version of the patient enablement instrument (PEI[27]) that has been validated in previous RCTs[28]. The modified PEI is a 7-point scale consisting of 6 items, in which higher scores indicate more enablement.
- Patient satisfaction was measured by asking patients whether they saw any benefits or disadvantages to using MBM, and whether they would recommend it to friends and family based on the NHS Friends and Family Test (FFT[29]).
- Patient burden was measured using a specifically developed questionnaire exploring time and costs via self-report based on Burden of Treatment Theory[30]. The questionnaire consisted of 4 questions with descriptive responses that explored whether new programmes were signed up to (such as gym membership, yoga/meditation), and the financial burden of doing so (see Appendix 1).

Physiological measures of lung function were taken at baseline and 12 month appointments: forced expiratory volume (FEV_1), ratio of forced expiratory volume to forced vital capacity (FEV_1/FVC) and peak expiratory flow rate (PEFR).

At 12-month follow-up, we also monitored health resource use GP consultations, A&E visits, hospital admissions, asthma medication use and use of antibiotics for chest infections using GP practice patient notes.

Healthcare utilisation data were collected via retrospective notes review conducted by practice staff. Staff were provided with a template for reviewing data, and an instruction manual to ensure correct data were provided. Initial notes reviews were completed within two months from completion of primary data collection. Nine patients (10% of the total patients in each practice) were also reviewed by research nurses to assess data quality.

Patient and Public Involvement

For peer review only

For peer review only - http://bmjopen.bmj.com/site/about/guidelines.xhtml

BMJ Open

Asthma UK was involved in the initial project proposal and supported the project throughout. Patient and public representatives (recruited with help from Asthma UK) participated in intervention development (providing feedback on prototype versions of the intervention, attending study management meetings, helping to develop trial materials and procedures, and discussing responses to participant feedback). Asthma UK are involved in dissemination of this research and ongoing projects related to the research.

Data Analysis

Primary analysis of the study was a description of key feasibility outcomes including patient eligibility, recruitment rates, withdrawals, 3 and 12-month follow-up response rates and digital intervention usage.

Descriptive statistics were used to identify any floor or ceiling effects. For continuous measures, means, standard deviations (SD) and 95% CIs were reported at baseline, 3-month and 12-month for each group, as well as for the sample as whole.

Exploratory analysis explored group differences in continuous outcome measures (AQLQ, ACQ, HADS, PEI, ICECAP-A, EQ-5D-5L) using linear regression models. Participants were analysed in the group to which they had been randomised and comprised complete cases only.

Proportions of patients achieving a minimal clinically important difference (MCID) was described for asthma quality of life (AQLQ).

Sensitivity analysis explored missing data at 3 and 12 months.

Health economic analysis was descriptive, reporting estimates of cost and outcomes measures and baseline and follow-up. The completeness and suitability of EQ-5D-5L and ICECAP were compared as was the appropriateness of the resource use, and time and cost tools developed for the study.

Intervention engagement was descriptive.

For peer review only - http://bmjopen.bmj.com/site/about/guidelines.xhtml

BMJ Open

Results

Recruitment and retention

Six practices were initially recruited and after monitoring recruitment rates a seventh practice added. In this additional practice only half the list (randomly selected) were offered participation in the study to avoid over recruiting. Across the 7 practices, 68478 patients were assessed for eligibility with 3199 meeting initial eligibility criteria (asthma diagnosis, >1 asthma medicine prescription in last 12 months, screened by practice). 266 patients completed postal screening measures before the recruitment period finished, of whom 125 were eligible to take part (impaired asthma-related quality of life, AQLQ score less than 5.5). Ninety patients responded to further contact. Two patients did not attend their baseline appointment leaving a final sample of 88 patients (intervention N = 44, usual care N = 44) who were recruited into the study (13.5 per practice) and were randomised over a 5 month period. Figure 1 presents the study CONSORT diagram.

During the study, 2 patients withdrew before 3-month follow-up and 2 before 12-month follow-up. All were in the intervention group. Patients withdrew for several reasons including lack of time (N = 1), illness (N = 1), death of family member (N = 1), and lack of perceived benefit (N = 1). 3 of 4 participants who withdrew had used the intervention; one had not. One participant was withdrawn from the study prior to 12-month follow-up they were no longer eligible (i.e. they were referred to secondary care).

Follow-up rates at 3 months were 91% (80/88; intervention: 36/44, control 44/44). Six (7%) patients did not complete 3 month follow-up measures but did not withdraw (all in intervention group).

At 12 months, 91% of participants provided primary outcome data by attending a follow-up appointment or returning a postal questionnaire (80/88; intervention: 37/45; control 43/43). 76% attended a baseline appointment and provided secondary clinical data (67/88). Four (5%) patients did not complete 12 month follow-up measures but did not withdraw (3 in intervention

For peer review only - http://bmjopen.bmj.com/site/about/guidelines.xhtml

BMJ Open

group; 1 in usual care). None of these intervention participants had used the intervention. None of these patients responded to efforts to contact them by the study team.

For peer review only

For peer review only - http://bmjopen.bmj.com/site/about/guidelines.xhtml

BMJ Open

Patient characteristics

Demographics and baseline characteristics of participants are presented in Table 1 and were reasonably well-balanced between arms across all measures.

Table 1: Baseline demographic characteristics of study population per group.

M (SD)		Overall sample (N = 88)	Intervention group (N = 44)	Control Group (N = 44)	Lost to follow-up (N = 8)
**Age M**		56.6 (15.2)	57.0 (14.2)	56.3 (16.2)	53.5 (12.11)
**Female N (%)**		53.0 (60.2)	27.0 (61.4)	26.0 (59.1)	6 (75)
**BMI**		29.5 (6.1)	28.9 (5.9)	30.1 (6.3)	32.7 (4.3)
**Length of diagnosis**		24.0 (17.5)	25.2 (17.2)	22.8 (17.8)	30 (18.9)
**FEV_1**		2.5 (0.8)	2.6 (0.8)	2.5 (0.8)	2.40 (0.47)
**% Predicted FEV_1**		92.3 (16.0)	94.8 (16.0)	89.8 (15.8)	92.0 (12.9)
**FEV_1 / FVC**		76.6 (8.5)	77.1 (8.0)	76.1 (9.0)	74.9 (4.1)
**Peak Flow**		421.2 (104.7)	421.3 (108.3)	421.1 (102.3)	420.6 (83.8)
**Ethnicity**	**White N (%)**	84 (95.5)	42 (95.5)	42 (95.5)	7 (87.5)
	**Other N (%)**	4 (4.5)	2 (4.5)	2 (4.5)	1 (12.5)
**Smoking Status**	**Current N (%)**	9 (10.2)	7 (15.9)	2 (4.5)	2 (25.0)
	**Former N (%)**	29 (33.0)	13 (29.5)	16 (36.3)	3 (37.5)
	**Never N (%)**	50 (56.8)	24 (54.5)	26 (59.1)	3 (37.5)
**Age left education**		18.5 (5.3)	19.4 (7.0)*	17.7 (2.7)	20.4 (8.2)
	**16 or under N (%)**	40 (46.5)	18 (42.9)	22 (50.0)	4 (50.0)
	**17-18 N (%)**	22 (25.6)	9 (21.4)	13 (29.5)	1 (12.5)
	**Above 18 (%)**	24 (27.9)	15 (35.7)	9 (20.5)	3 (37.5)
**Index of Multiple Deprivation Mean Rank (Median Decile)**		17192 (5.5)	17231 (6.5)	17212 (5)	4505.5 (1.5)
**AQLQ M**		4.81 (1.01)	4.85 (0.94)	4.78 (1.09)	4.26 (0.55)
**ACQ**		1.45 (0.80)	1.35 (0.66)	1.56 (0.91)	1.52 (0.73)
**HADS-A**		6.60 (4.47)	6.57 (3.87)	6.64 (5.04)	8.63 (3.9)
**HADS-D**		3.89 (3.57)	3.39 (3.07)	4.39 (3.99)	4.75 (4.4)
**EQ5D-5L**		0.83 (0.19)	0.86 (0.15)	0.81 (0.22)	4 (50.0)
**EQ5D-VAS**		71.5 (18.2)	70.0 (19.3)	73.0 (17.2)	1 (12.5)
**ICECAP-A**		0.87 (0.18)	0.89 (0.12)	0.88 (0.16)	3 (37.5)
**PEI**		2.52 (1.23)	2.44 (1.09)	2.60 (1.37)	2.73 (1.0)
**MARS-A**		4.70 (1.05)	4.80 (0.90)	4.60 (1.20)	4.3 (0.8)

Note: (*) Percentages are reported from 42 participants as two participants in the intervention group did not complete this data.

For peer review only

BMJ Open

Table 1 compares those lost to follow-up to those who remained in the study at 3 months. Those lost to follow up were slightly more likely to be female, have a higher BMI, a longer time since diagnosis, a lower AQLQ score, a higher HADS-A and HADS-D score and to be from a more deprived postcode.

Intervention usage and engagement

At 12 month follow-up, 36 (82%) patients in the intervention arm had engaged with the intervention (at least 1 log in). Patients logged in between 0 and 25 times to the intervention (Median=4; IQR=8.25). Several patients also engaged with additional lifestyle modification interventions including improving hand hygiene (N = 2), weight loss (N = 3), improving physical activity (N = 3) and getting support from friends and family (N = 5).

After the study, participants in the intervention group were asked ‘Do you think there were any benefits to using My Breathing Matters?’. 12 of 36 (33%) reported ‘quite a bit/a large amount of benefit’, 19/36 (53%) reported ‘some benefit’, and 5/36 (14%) reported ‘very little benefit’. 22 participants completed a free text box describing the advantages - benefits varied but included information provision (such as ‘weight loss’, ‘dietary/exercise regimes’), medication adherence (such as asthma action plans, improved medication adherence), provision of non-pharmacological treatments (such as breathing exercises and relaxation) and accessibility (such as ‘access to information quickly’). This is reported in more detail in a separately published process analysis.

Participants were also asked ‘Do you think there were any disadvantages to using My Breathing Matters?’. Twenty five of 36 (69%) reported no disadvantages at all, 3 (8%) reported very few disadvantages, 8 (22%) reported some disadvantages, and 0 reported quite a bit or a large amount of disadvantages. 13 participants completed a free text box describing disadvantages, which included technical difficulties (such as not always accessible across different devices, difficulty logging in) and information specificity (such as not enough information, too many

For peer review only - http://bmjopen.bmj.com/site/about/guidelines.xhtml

BMJ Open

reminders, too few reminders). A final question asked how likely participants were to recommend MBM to friends or family. Sixteen participants (44%) were extremely likely to recommend it, 12 participants (33%) were likely, 7 (19%) were neither likely nor unlikely, and 1 was extremely unlikely (3%).

Trial Endpoint Measures

The full data of the trial endpoints is set out in Table 2.

Both the intervention group and control group improved from baseline to 3-month and 12-month follow-up, with numerically (but not statistically significantly) larger improvements in the asthma-related patient reported outcomes measuring quality of life and symptom control (AQLQ and AQC) at both time points; one or both these measure are anticipated to to be the primary outcome of a subsequent fully powered study.

At the 3-month evaluation, patients in the intervention group who completed 3 month follow-up measures (N = 36) had mean improvement in asthma-related quality of life (AQLQ score) of 0.53 (95% CI: 0.31, 0.75), and in the control group of 0.52 (95%CI: 0.30, 0.74), with the between-group difference (controlling for baseline differences) the AQLQ being 0.06 higher (95% CI 0.35, -0.22) in the intervention group, indicating better control. By 12 months, these figures were 0.35 (0.10, 0.60) and 0.21 (-0.09, 0.51) respectively, and the between-group difference had risen to 0.18 (95% CI 0.56, -0.21) higher in the intervention group. In the ACQ analysis, at the 3-month analysis, the between-groups ACQ score was 0.14 lower (95% CI 0.13, -0.41) in the intervention group, indicating better control, and at 12 months was 0.14 lower (95% CI 0.11, -0.40). These findings indicate consistent trends to improvement in both asthma quality of life and asthma control in the intervention group compared to the control. Full follow-up data are presented in Table 2.

For peer review only - http://bmjopen.bmj.com/site/about/guidelines.xhtml

BMJ Open

Table 2: 3 and 12-month follow up data (corrected for baseline differences)

Measure	Intervention group (N = 36)			Control Group (N = 44)			Difference between the intervention and control group controlling for baseline (95% CI)
	M (SD)	% >MCID* Improvement	% items complete	M (SD)	% >MCID* Improvement	% items complete	
**3-month**							
AQLQ	5.51 (0.85)	47.2	82	5.30 (1.07)	47.7	100	-0.06 (-0.35, 0.22)
ACQ	0.98 (0.65)		82	1.28 (0.87)		100	0.14 (-0.13, 0.41)
HADS-A	6.75 (3.85)		82	7.07 (5.48)		100	0.04 (-0.11, 0.18)
HADS-D	3.75 (2.82)		82	4.66 (4.99)		100	0.02 (-0.13, 0.16)
PEI	2.71 (1.09)		82	2.90 (1.14)		100	0.12 (-0.35, 0.59)
MARS-A	4.23 (0.70)		80	4.05 (0.74)		100	-0.04 (-0.33, 0.25)
EQ-5D-5L	0.82 (0.19)		82	0.83 (0.20)		100	-
ICECAP-A	0.87 (0.12)		82	0.84 (0.19)		100	-
**12-month**							
AQLQ	5.29 (0.98)	38.9	82	5.00 (1.25)	39.5	98	-0.18 (-0.56, 0.21)
ACQ	1.00 (0.59)		82	1.26 (0.69)		98	0.14 (-0.11, 0.40)
HADS-A	7.78 (3.94)		84	6.63 (4.91)		98	-0.99 (-2.15, 0.16)
HADS-D	3.81 (3.54)		84	4.19 (4.17)		98	-0.22 (-1.41, 0.97)
PEI	2.46 (1.03)		84	2.61 (1.28)		98	0.09 (-0.37, 0.54)
MARS-A	4.37 (0.81)		82	4.29 (0.85)		98	0.09 (-0.25, 0.43)
EQ-5D-5L	0.83 (0.21)		82	0.80 (0.23)		98	-
ICECAP-A	0.86 (0.13)		82	0.84 (0.20)		98	-
FEV_1 (litres)	2.75 (0.75)		57	2.43 (0.74)		80	-0.03 (-0.10, 0.05)
FEV_1 / FVC	78.8 (6.58)		57	76.3 (9.29)		80	-2.20 (-4.27, -0.13)
% Predicted FEV_1	100.1 (14.8)		57	92.4 (13.8)		80	-1.77 (-5.25, 1.72)
Peak Flow	450 (105)		57	417 (102)		80	-15.29 (-36.86, 6.27)
BMI	28.7 (6.17)		64	31.1 (6.51)		86	0.11 (-0.68, 0.89)

There was no difference in number of patients who showed MCID improvement at 3 months (AQLQ, >0.5) across groups (47.2% in the intervention group compared to 47.7% in the control group). The same was true at 12 months (38.9% compared to 39.5%).

For peer review only

BMJ Open

Adverse Events

Adverse events were reported by GPs and nurses who contacted the study team to report both adverse and serious adverse events. Nine adverse events were reported (intervention N = 6, usual care N = 3). These were assessed by research team clinicians and all were considered unlikely to be related to the study. Three were related to participant asthma (asthma exacerbation not leading to hospital admission, upper respiratory tract infection, sinusitis).

Three serious adverse events were reported (intervention N = 2, usual care N = 1). These were considered unlikely to be related to the study and the condition (atrial fibrillation, open distal radius fracture, cardioversion).

Healthcare Utilisation Outcomes

Data were collected from retrospective notes reviews (conducted by practice nurses) from 83 participants, reported in Table 3. Data was collected from 84 practices for 7 participants, with 4 participants from 1 practice incomplete. The data quality check, and subsequent examination by research team clinicians (MT) found that reviews completed by the practice nurses varied substantially in quality with varied levels of detail, and the quality of data achieved in this way was insufficient for a health economic analysis.

Table 3: Data on asthma related medication use (during the study period)

Healthcare utilisation (N, IQR) Mean (SD)	Intervention group		Control group	
	12m before study period	12m after study period	12m before study period	12m after study period
SABA Prescriptions	3 (2,6)<br>3.92 (3.48)	3 (1,6)<br>4.00 (3.72)	3 (2,5)<br>4.0 (3.89)	4 (2,6)<br>4.39 (3.81)
ICS Prescriptions	5 (2,11)<br>6.72 (4.92)	4 (3,10)<br>6.15 (4.21)	6 (4,10)<br>7.41 (5.45)	6 (4,10)<br>7.34 (5.37)
Oral steroids prescriptions	0 (0,0)<br>0.31 (0.80)	0 (0,0)<br>0.36 (0.94)	0 (0,0)<br>0.43 (1.07)	0 (0,0)<br>0.23 (0.71)
Antibiotic Prescriptions	0 (0,0)<br>0.33 (0.87)	0 (0,0)<br>0.28 (0.60)	0 (0,0)<br>0.52 (1.45)	0 (0,1)<br>0.16 (0.48)

Note: (*) Incidence rate ratio vs. intervention group, controlling for 12 months before study period.

For peer review only - http://bmjopen.bmj.com/site/about/guidelines.xhtml

BMJ Open

Comparisons between group count data were reported using a negative binomial model but given the issues with the reliability of the data, should be interpreted cautiously. The prescription rate was approximately 8% higher in for both SABA (IRR 1.08, 95% CI 0.82, 1.43) and ICS (IRR 1.08, 95% CI 0.86, 1.35) in the control group compared to the intervention group. Both groups had a low number of prescriptions for oral steroids, oral steroids and antibiotics, with only 15 prescriptions in total for either of these medications, making between group comparisons unreliable.

Due to unreliability of data, frequency of GP consultations, A&E admissions and hospitalisations have not been reported.

Health Economic Outcomes

Both EQ-5D-5L and ICECAP-A had the same completion rates as other secondary measures completed at follow up (see Table 2).

Patients reported several programmes across both groups including gym, walking, yoga, sewing, language courses, physio and signing (see Appendix 2). There were no substantial differences in terms of numbers or costs although the sample size was small.

Discussion

Findings from our randomised controlled feasibility trial demonstrate that a full-size confirmatory trial to confirm effectiveness of MBM, a digital self-management intervention for adults in primary care with asthma is feasible and acceptable. There were also trends to improved asthma control and quality of life in our underpowered sample, so supporting the need for a definitive fully-powered study. Our recruitment procedures recruited a specific patient sample (those impaired asthma-specific quality of life) to target from a range of urban and rural practices.

For peer review only - http://bmjopen.bmj.com/site/about/guidelines.xhtml

BMJ Open

Our sample varied in age with a relatively high mean (56 years) indicating that our digital intervention can provide benefit to older adults. Both male and female adults were well-represented in our trial. A notable proportion of our sample was obese (41%), in line with previous findings[11]. Given that obesity is a risk factor for asthma, a larger trial could further improve effectiveness by providing more specific behavioural content for obese adults with asthma (such as tailored content to increase motivation to use weight-loss related lifestyle components in obese patients). Our sample was also predominantly white. Underrepresentation of minority ethnic groups in medical research in the UK is an ongoing issue[12] and should be addressed in recruitment procedures in the full trial.

Trial procedures were completed effectively, supporting the feasibility of a full trial. All patients who completed baseline measures were randomised. Completion of measures was good at both follow-up points (3-month via post and 12-month at participants' practice). Where participants were not able to attend a follow-up appointment at practices, they were satisfactorily followed up via post or telephone for main trial measures. 8 patients were lost to follow-up (4 withdrew and 4 no longer responded to attempts to contact them). Notably, these patients were all in the intervention group; it may be that the control group were more likely to maintain contact as they were only able to access the intervention upon completion of 12-month follow up measures. Although loss to follow-up is low, it is important to consider whether that loss is differential. Those lost to follow-up were more likely to be more socio-economically deprived, female, have a higher BMI, a longer time since diagnosis and a higher HADS-A and HADS-D score. It is possible that these patients would benefit from using MBM more than most, and therefore we have proposed several ways to further increase trial efficacy. Automatic email intervention registration at baseline (patients cannot attend baseline appointment without enrolling on the intervention) would increase initial engagement and engagement with trial procedures throughout duration of study. Online questionnaire completion during screening process would i) screen patients who are unable to interact with online trial/intervention and

For peer review only - http://bmjopen.bmj.com/site/about/guidelines.xhtml

therefore unable to benefit from the intervention (feasibility trial estimate = 2%), and ii) streamline baseline/follow-up procedures.

Both health economic outcomes had high completion rates but did not suggest substantive change, similar to EQ-5D measures in previous non-pharmacological self-management trials (such as [8]). It is possible that an alternative measure such as the Short Form 12-item Survey (SF-12[31]) in which participants consider the previous two weeks (whereas in the EQ-5D they consider the immediate present) may be better suited to measure small yet valuable changes in well-being over a full trial. Our detailed mixed-methods process analysis explored issues of trial acceptability in more detail, and will be reported in a subsequent paper. A full trial of this non-pharmacological intervention should accurately capture 'non-medical' costs (such as gym membership) that are likely to impact disease-specific quality of life, as well as medical costs that would be affected by changes in healthcare utilisation.

Healthcare utilisation data were collected by practice nurses whose main role was to provide usual clinical care at the practices, using a manual to guide data collection, rather than by trained research nurses, and our quality check demonstrated that the data collection process used was unreliable some centres. We conclude that in a full subsequent study, these data should be collected from the medical record by a trained member of the study team (such as a trained research nurse), as has been successfully used in previous studies[8].

Engagement with the intervention was increased compared to a previous similar digital asthma self-management interventions[18] at initial sign up (82% vs 76%) as well as maintaining a higher number of ongoing engagement throughout the follow up period (median 3 additional log ins vs. 1), although our study used a broad primary care population while the RAISIN protocol primarily recruited from areas of high deprivation. This finding demonstrates that the use of the person-based approach to develop the intervention resulted in an intervention that was acceptable and engaging to patients, even using a pragmatic methodology in which patients

For peer review only - http://bmjopen.bmj.com/site/about/guidelines.xhtml

BMJ Open

self-registered at home instead of being registered by a GP during their baseline appointment. Participants accessed both pharmacological and non-pharmacological self-management content.

Estimates of effect size demonstrated that participants who received the intervention and completed follow-up measures showed improved and clinically relevant quality of life and asthma control. The order of magnitude of the mean between-group improvements in the patient reported measures of control (ACQ) and asthma-related QOL (AQLQ), although not statistically significant with the sample size of this feasibility study, was comparable to that reported in controlled studies of pharmacological[32] and non-pharmacological[8] interventions in asthma, and so justify a fully powered study.

There was no suggestion of an effect on physiological measures of lung function. These results are in line with previous studies of behavioural self-management interventions in primary care adults with asthma (such as BREATHE, RAISIN), and demonstrate the importance of interventions targeting outcomes that incorporate elements of functional wellbeing (disease specific quality of life, subjective symptoms), rather than solely focusing on objective, physiological measures that are not correlated with quality of life.

The effectiveness of our intervention could be further increased according to findings from our process evaluation. This analysis, which will be reported separately, broadly agrees with previous research[15] in finding that many patients consider their asthma to be 'well-controlled' despite having important levels of symptoms and quality of life impairment on validated questionnaire. This implies that many people had become accustomed to their on-going symptoms and had altered their life to try to reduce their impact, using denial as a coping mechanism. As a consequence, the means of appropriately targeting and framing self-management interventions should be carefully considered in future work, focussing on maintaining good health rather than improving poor health. Some of our findings (such as the association between quality of life improvement and ongoing intervention engagement) demonstrate that framing content as positive and not focusing on illness – for example 'How to

For peer review only - http://bmjopen.bmj.com/site/about/guidelines.xhtml

keep your breathing healthy' rather than 'How to reduce asthma symptoms' may lead to an acceptable, engaging intervention that benefits this patient group.

There were some limitations to this small feasibility study. Although our researchers and statisticians were blind to group allocation, patients would have known that they were allocated to the intervention rather than the usual care control. This is common in complex behavioural interventions. Furthermore, although we endeavoured to recruit participants across a broad demographic range, the reach of our intervention could be improved. While the reach of digital interventions improves as digital literacy increases nationally, care must be taken to ensure that 'digital transformation' of NHS services does not entrench healthcare inequality, by facilitating a 'digital divide' that fails to provide adequate health and social care to those who do not have the digital skills to benefit.

Conclusion

Our findings demonstrate the feasibility of a new digital self-management intervention for asthma (MBM). Using the person-based approach to intervention development means that MBM is both acceptable and engaging for adults with asthma in primary care. MBM reflects the varied experiences of people with asthma, by including both non-pharmacological and pharmacological components. Our data support the feasibility of moving towards a fully-powered RCT, with only minor modifications to some trial procedures required.

For peer review only - http://bmjopen.bmj.com/site/about/guidelines.xhtml

Acknowledgements: The authors would like to acknowledge the contributions of all participants and patient and public representatives in the development of the My Breathing Matters intervention development and feasibility evaluation, and Dr Deborah Morrison who lead the development of 'Living Well With Asthma' resource that guided development of My Breathing Matters.

Protocol: The trial protocol has been along with this publication as a supplementary file (Supplementary File 1).

Ethics: The study was approved by NHS South Central – Berkshire Research Ethics Committee, 16/01/2017, ref: 16/SC/0614.

Competing Interests: Neither MT nor any member of his close family has any shares in pharmaceutical companies. In the last 3 years he has received speaker's honoraria for speaking at sponsored meetings or satellite symposia at conferences from the following companies marketing respiratory and allergy products: GSK, Novartis. He has received honoraria for attending advisory panels with; Boehringer Inglehiem, GSK, Novartis. He is a recent a member of the BTS SIGN Asthma guideline steering group and the NICE Asthma Diagnosis and Monitoring guideline development group. BA, KG, JR, BS, LY, FM and AB have no competing interests.

Author Contributions: LY, MT and AB conceived the idea for the study. LY, MT, JR, FM, BS & AB secured funding for the study. BA, LY, AB, BS MT developed the intervention and designed the trial with input from Asthma UK and FM. BA and KG managed the trial on a day-to-day basis with support from LY and MT. BS and JR planned and carried out the statistical analysis. BA drafted the manuscript with assistance and final approval from all authors. BA is the guardian of the data.

Funding Statement: This article presents independent research funded by the National Institute for Health Research (NIHR) Programme Grant for Applied Research (PGfAR RP-PG-1211-20001) awarded to LY. LY is an NIHR Senior Investigator and during the study BA was supported by an NIHR School of Primary Care (SPCR) Fellowship. Prof Anne Bruton was funded

For peer review only - http://bmjopen.bmj.com/site/about/guidelines.xhtml

BMJ Open

by the National Institute for Health Research (NIHR) Senior Research Fellowship (SRF-2012-05-120). The views expressed are those of the author(s) and not necessarily those of the NIHR or the Department of Health and Social Care.

Data Sharing: Anonymised data is available at reasonable request (in line with consent provided by participants) from the corresponding author

Word Count: 5366.

For peer review only

For peer review only - http://bmjopen.bmj.com/site/about/guidelines.xhtml

BMJ Open

References

1. Global Asthma Network. The Global Asthma Report 2014. Vol. 5, Auckland, New Zealand. 2014. 14–16

2. Demoly P, Gueron B, Annunziata K, Adamek L, Walters RD. Update on asthma control in five European countries: Results of a 2008 survey. Eur Respir Rev. 2010;19(116):150–7. doi: 10.1183/09059180.00002110.

3. Guidelines for management of asthma in adults: I--Chronic persistent asthma. Statement by the British Thoracic Society, Research Unit of the Royal College of Physicians of London, King's Fund Centre, National Asthma Campaign. BMJ [Internet]. 1990 Sep 29 [cited 2019 Jun 17];301(6753):651–3. Doi: 10.1136/bmj.301.6753.651

4. Ainley A, Gildeh N, Seeley A, Lewis K, Maisel T, Jayasinghe R, et al. Does having specialist asthma nurses improve adherence to national asthma guidelines? A comparison between two UK district general hospitals. In: 111 Clinical Problems - Asthma [Internet]. European Respiratory Society; 2015 [cited 2019 Jun 17]. p. PA644. Doi: 10.1183/13993003.congress-2015.PA644.

5. Gibson PG, Powell H, Coughlan J Wilson AJ, Abramson M, Haywood P, Bauman A, Hensley MJ, Walters EH. Self-management education and regular practitioner review for adults with asthma. Cochrane Database Syst Rev. 2002;1:CD001117. doi: 10.1002/14651858.CD001117.

6. Pinnock H, Parke HL, Panagioti M, Daines L, Pearce G, Epiphaniou E, et al. Systematic meta-review of supported self-management for asthma: a healthcare perspective [Internet]. Vol. 15, BMC Medicine. BMC Medicine; 2017. doi: 10.1186/s12916-017-0823-7

7. Asthma UK. Annual Asthma Survey 2018 [Internet]. 2018 [cited 2019 Jun 17].

8. Bruton A, Lee A, Yardley L, Raftery J, Arden-Close E, Kirby S, et al. Physiotherapy breathing retraining for asthma: a randomised controlled trial. Lancet Respir Med [Internet]. 2017;2600(17):1–10. doi: 10.1016/S2213-2600(17)30474-5.

9. Department of Health and Social Care. The future of healthcare: our vision for digital, data and technology in health and care [Internet]. UK Government Policy Paper. 2018 Oct [cited 2019 Jun 17].

10. van Gaalen JL, Beerthuizen T, van der Meer V, van Reisen P, Redelijkheid GW, Snoeck-Stroband JB, et al. Long-term outcomes of internet-based self-management support in adults with asthma: randomized controlled trial. J Med Internet Res [Internet]. 2013 Sep 12 [cited 2019 Jun 17];15(9):e188. doi: 10.2196/jmir.2640

11. Morrison D, Wyke S, Saunderson K, McConnachie A, Agur K, Chaudhuri R, et al. Findings from a pilot Randomised trial of an Asthma Internet Self-management Intervention (RAISIN). BMJ Open [Internet]. 2016 May 12 [cited 2019 May 28];6(5):e009254. doi: 10.1136/bmjopen-2015-009254.

12. McLean G, Band R, Saunderson K, Hanlon P, Murray E, Little P, et al. Digital interventions to promote self-management in adults with hypertension systematic review and meta-analysis. BMC Pulm Med [Internet]. 2016;34(4):600–12. doi: 10.1097/HJH.0000000000000859.

13. Band R, Bradbury K, Morton K, May C, Michie S, Mair FS, et al. Intervention planning for a digital intervention for self-management of hypertension: a theory-, evidence- and person-based approach. Implement Sci [Internet]. 2017;12(1):25. doi: 10.1186/s13012-017-0553-4.

For peer review only - http://bmjopen.bmj.com/site/about/guidelines.xhtml

BMJ Open

14. Moore GF, Audrey S, Barker M, Bond L, Bonell C, Hardeman W, et al. Process evaluation of complex interventions: Medical Research Council guidance. BMJ [Internet]. 2015 Mar 19 [cited 2019 Jun 17];350:h1258. Doi: 10.1136/BMJ.H1258.

15. Smith T, Noble M, Noble S, Wright G, McLennan D, Plunkett E. The English indices of deprivation 2015. London: Department for Communities and Local Government. 2015 Sep.

16. Juniper EF, Guyatt GH, Cox FM, Ferrie PJ, King DR. Development and validation of the Mini Asthma Quality of Life Questionnaire. Eur Respir J [Internet]. 1999 Jul 1 [cited 2019 Jun 17];14(1):32–8. Doi: 10.1034/j.1399-3003.1999.14a08.x

17. Hoffmann TC, Glasziou PP, Boutron I, Milne R, Perera R, Moher D, et al. Better reporting of interventions: template for intervention description and replication (TIDieR) checklist and guide. Bmj [Internet]. 2014;348(mar07 3):g1687–g1687. doi: 10.1136/bmj.g1687.

18. Yardley L, Osmond A, Hare J, Wills G, Weal M, De Roure D, et al. Introduction to the LifeGuide: software facilitating the development of interactive behaviour change internet interventions. In: Proceedings of the WebSci'09: Society On-Line. Athens, Greece [Internet]. 2009 [cited 2019 Jun 17].

19. Morrison D, Mair FS, Chaudhuri R, McGee-Lennon M, Thomas M, Thomson NC, et al. Details of development of the resource for adults with asthma in the RAISIN (randomized trial of an asthma internet self-management intervention) study. BMC Med Inform Decis Mak [Internet]. 2015;15(1):57. doi: 10.1186/s12911-015-0177-z.

20. Yardley L, Morrison L, Bradbury K, Muller I. The Person-Based Approach to Intervention Development : Application to Digital Health-Related Behavior Change Interventions Corresponding Author : J Med Internet Res. 2015;17(1):e30. doi: 10.2196/jmir.4055.

21. Yardley L, Ainsworth B, Arden-Close E, Muller I. The person-based approach to enhancing the acceptability and feasibility of interventions. Pilot Feasibility Stud [Internet]. 2015 Dec 26 [cited 2019 Apr 26];1(1):37. doi: 10.1186/s40814-015-0033-z.

22. Asthma UK. Live Well With Asthma Booklet [Internet]. 2016. Available from: https://www.asthma.org.uk/197182a8/globalassets/health-advice/resources/adults/live-well-with-asthma-booklet.pdf

23. Juniper EF, O'Byrne PM, Guyatt GH, Ferrie PJ, King DR. Development and validation of a questionnaire to measure asthma control. Eur Respir J [Internet]. 1999;14:902–7. Doi: 10.1034/j.1399-3003.1999.14d29.x.

24. EuroQol - a new facility for the measurement of health-related quality of life. Health Policy (New York) [Internet]. 1990 Dec 1 [cited 2019 Mar 8];16(3):199–208. doi: 10.1016/0168-8510(90)90421-9.

25. Al-Janabi H, N Flynn T, Coast J. Development of a self-report measure of capability wellbeing for adults: the ICECAP-A. Qual Life Res [Internet]. 2012 Feb 20 [cited 2019 Mar 8];21(1):167–76. doi: 10.1007/s11136-011-9927-2.

26. Zigmond AS, Snaith RP. The Hospital Anxiety And Depression Scale. Acta Psychiatr Scand [Internet]. 1983;67(6):361–70. doi: 10.1186/1477-7525-1-29.

27. Howie JG, Heaney DJ, Maxwell M, Walker JJ. A comparison of a Patient Enablement Instrument (PEI) against two established satisfaction scales as an outcome measure of primary care consultations. Fam Pract [Internet]. 1998 Apr 1 [cited 2019 Jun 17];15(2):165–71. doi: 10.1093/fampra/15.2.165.

28. Little P, Lewith G, Webley F, Evans M, Beattie A, Middleton K, et al. Randomised controlled

For peer review only - http://bmjopen.bmj.com/site/about/guidelines.xhtml

BMJ Open

trial of Alexander technique lessons, exercise, and massage (ATEAM) for chronic and recurrent back pain. BMJ [Internet]. 2008 Aug 19 [cited 2019 Jun 17];337:a884. doi: 10.1136/BMJ.A884.

29. Sizmur S, Graham C, Walsh J. Influence of patients' age and sex and the mode of administration on results from the NHS Friends and Family Test of patient experience. J Health Serv Res Policy [Internet]. 2015 Jan 27 [cited 2019 Jun 17];20(1):5–10. doi: 10.1177/1355819614536887

30. May CR, Eton DT, Boehmer K, Gallacher K, Hunt K, MacDonald S, et al. Rethinking the patient: using Burden of Treatment Theory to understand the changing dynamics of illness. BMC Health Serv Res [Internet]. 2014 Dec 26 [cited 2019 Jun 17];14(1):281. doi: 10.1186/1472-6963-14-281.

31. Ware J, Kosinski M, Keller SD. A 12-Item Short-Form Health Survey: construction of scales and preliminary tests of reliability and validity. Med Care [Internet]. 1996 Mar [cited 2019 Jun 17];34(3):220–33. doi: 10.2307/3766749

32. Bateman ED, Esser D, Chirila C, Fernandez M, Fowler A, Moroni-Zentgraf P, et al. Magnitude of effect of asthma treatments on Asthma Quality of Life Questionnaire and Asthma Control Questionnaire scores: Systematic review and network meta-analysis. J Allergy Clin Immunol [Internet]. 2015 Oct 1 [cited 2019 Jun 17];136(4):914–22. doi: 10.1016/J.JACI.2015.03.023.

For peer review only - http://bmjopen.bmj.com/site/about/guidelines.xhtml

BMJ Open

Figure Captions

Figure 1. Study Consort Diagram

For peer review only

For peer review only - http://bmjopen.bmj.com/site/about/guidelines.xhtml

Figure 1. Study Consort Diagram

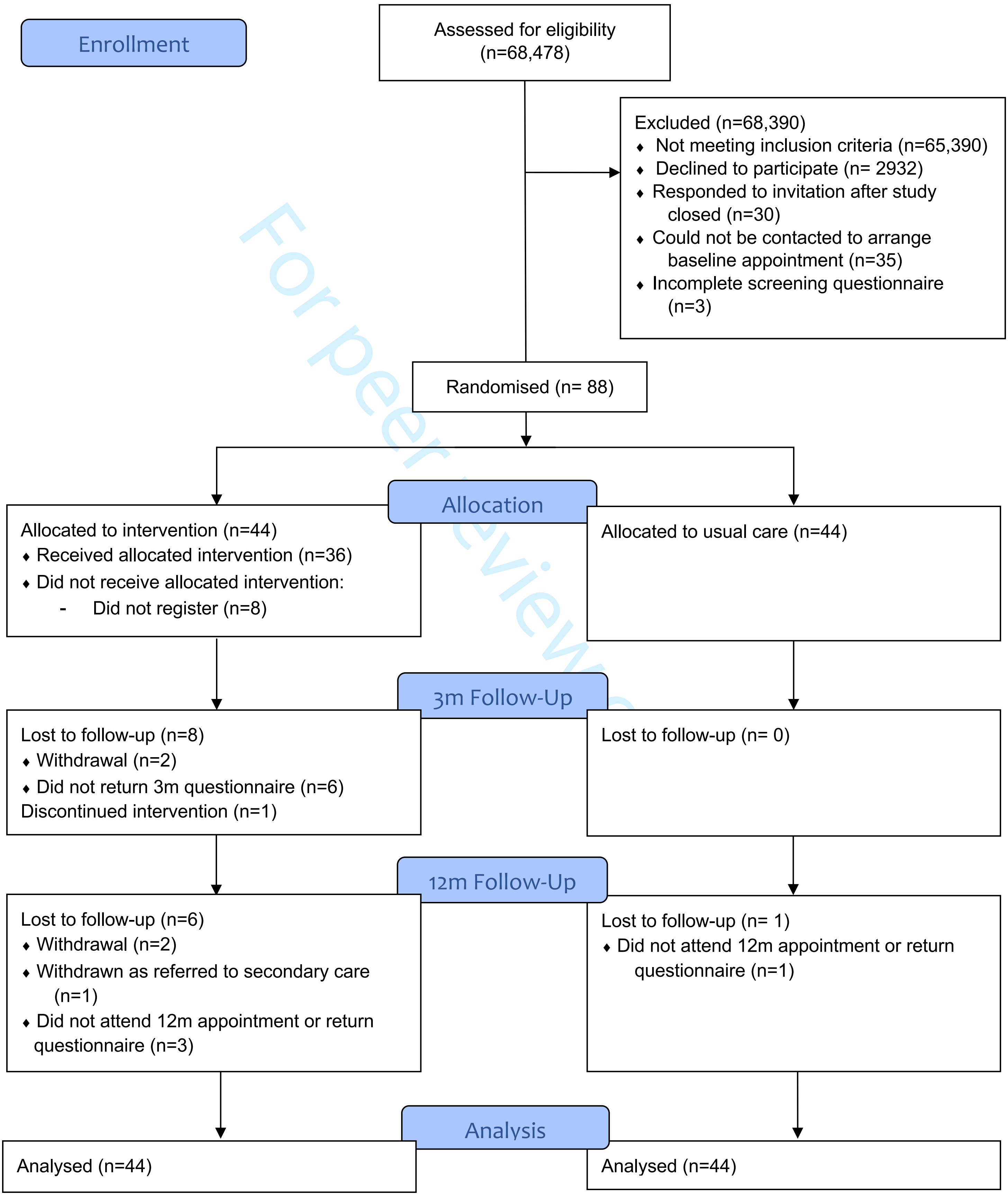

For peer review only

Patient Costs and Opportunities Questionnaire

Please complete the below questionnaire by ticking ONE box to answer each question.

1. In the last 12 months, have you signed up to any new programmes (e.g. gym membership, taken up a new activity like yoga/meditation) that might help your health?
   - ☐ No new programmes at all
   - ☐ Some new programmes
   - ☐ Lots of new programmes

Please note any programmes you took up:

____

____

2. Have any new programmes that you have signed up to (e.g. gym membership, taken up a new activity) cost you any money that you might have spent elsewhere?
   - ☐ Yes — Total Amount: ______ Weekly amount: ______ (if you don't know, please estimate)
   - ☐ No

Please only answer the below questions if you registered with the My Breathing Matters website in the last 12 months.

3. Did you think there were any benefits of using My Breathing Matters?
   - ☐ No benefit at all
   - ☐ Very little benefit
   - ☐ Some benefit
   - ☐ Quite a bit of benefit
   - ☐ A large amount of benefit

If any benefits, please note them down below:

____

____

4. Did you think there were any disadvantages of using My Breathing Matters?
   - ☐ No disadvantages at all
   - ☐ Very little disadvantages
   - ☐ Some disadvantages
   - ☐ Quite a bit of disadvantages
   - ☐ A large amount of disadvantages

If any disadvantages, please note them down below:

____

____

5. How likely are you to recommend My Breathing Matters to friends and family if they needed similar care and treatment?
   - ☐ Extremely likely
   - ☐ Likely
   - ☐ Neither likely or unlikely
   - ☐ Unlikely
   - ☐ Extremely unlikely
   - ☐ Don't know

For peer review only - http://bmjopen.bmj.com/site/about/guidelines.xhtml

Appendix 2. Time and costs Questionnaire summary

	Demographic characteristic	
	Intervention	Usual Care
In the last 12 months, have you signed up to any new programmes (e.g. gym membership, taken up a new activity like yoga/meditation) that might help your health?	Lots of new programmes (2%)<br>Some new programmes (27%)<br>No new programmes (50%)<br>Missing (21%)	Lots of new programmes (0%)<br>Some new programmes (36%)<br>No new programmes (69%)<br>Missing (4%)
Have any new programmes that you have signed up to (e.g. gym membership, taken up a new activity) cost you any money that you might have spent elsewhere?	Yes (14%)<br>No (64%)<br>Missing (23%)	Yes (21%)<br>No (68%)<br>Missing (11%)
Total amount spent on other programmes (M, SD)	363.75 (320.30)	336.25 (379.81)

For peer review only

UNIVERSITY OF Southampton

My Breathing Matters – Trial Protocol

‘My Breathing Matters’ - feasibility study of a digital self-management programme designed to improve the quality of life people with asthma.

For peer review only

Version 2, dated 07/12/2016

SPONSOR: University of Southampton

COORDINATING CENTRE: Centre for Applications of Health Psychology (supported by Southampton Clinical Trials Unit)

EudraCT reference no:	N/A
ISRCTN reference no:	TBC
**NHS Ethics reference no:**	**214457**

Protocol authorised by:

**Name:**	Prof. Lucy Yardley	**Role:**	Chief Investigator
**Signature:**	L Yardley	**Date:**	**07/12/2016**

MAIN TRIAL CONTACT

Chief Investigator: Professor Lucy Yardley

Centre for Applications of Health Psychology
Academic unit of psychology
University of Southampton
Southampton
SO17 1BJ

Tel: +44 (0)2380 594581
Email: L.Yardley@soton.ac.uk

TRIAL COORDINATION CENTRE

For general trial and clinical queries e.g. participant queries, trial supplies, data collection, please contact in the first instance:

Ben Ainsworth

Address: Centre for Applications of Health Psychology
Academic unit of psychology
University of Southampton
Southampton
SO17 1BJ

Tel:
Fax: 02380 591950
Email: ben.ainsworth@soton.ac.uk
Web:

SPONSOR

The University of Southampton is the research sponsor for this trial. For further information regarding sponsorship conditions, please contact:

Address: Diana Galpin
Head of IP, Contracts & Policy
University of Southampton
Southampton
SO17 1BJ

Tel: 02380 595058
Email: rgoinfo@soton.ac.uk

FUNDER

This trial is funded by NIHR PGfAR

Protocol Information

This protocol describes the My Breathing Matters trial and provides information about procedures for entering participants. The protocol should not be used as a guide for the treatment of other participants; every care was taken in its drafting, but corrections or amendments may be necessary. These will be circulated to investigators in the trial, but sites entering participants for the first time are advised to contact the Centre for Applications of Health Psychology to confirm they have the most recent version.

Compliance

This trial will adhere to the principles outlined in the International Conference on Harmonisation Good Clinical Practice (ICH GCP) guidelines. It will be conducted in compliance with the protocol, the Data Protection Act and all other regulatory requirements, as appropriate.

For peer review only

For peer review only - http://bmjopen.bmj.com/site/about/guidelines.xhtml

TABLE OF CONTENTS

LIST OF ABBREVIATIONS 5
KEYWORDS 5
TRIAL SYNOPSIS 6
SCHEDULE OF OBSERVATIONS AND PROCEDURES 9
1. INTRODUCTION 10
1.1 BACKGROUND 10
1.2 RATIONALE AND RISK BENEFITS FOR CURRENT TRIAL 10
2. TRIAL OBJECTIVES 11
3. TRIAL DESIGN 11
3.1 TRIAL OUTCOME MEASURES 12
4. CENTRE/PARTICIPANT SELECTION AND ENROLLMENT OF PARTICIPANTS 13
4.1 CENTRE SELECTION 13
4.2 SCREENING AND PRE-REGISTRATION 14
4.3 INCLUSION CRITERIA 14
4.4 EXCLUSION CRITERIA 15
4.5 REGISTRATION / RANDOMISATION PROCEDURES 15
4.6 CONSENT 15
4.7 WITHDRAWAL CRITERIA 15
6. INTERVENTION 16
6.1 INTERVENTION ARM 16
6.2 USUAL CARE 16
7. ADVERSE EVENTS AND REPORTING 17
7.1 DEFINITIONS 17
7.2 CAUSALITY 17
7.3 REPORTING PROCEDURES 18
7.3.2. Non serious AEs 18
7.3.3 Serious AEs 19
8. ASSESSMENT AND FOLLOW-UP OF PARTICIPANTS 19
8.1 DEFINITION OF END OF TRIAL 20
9. QUANTITATIVE EVALUATION 20
9.1 SAMPLE SIZE 20
9.2 RANDOMISATION 20
9.3 STATISTICAL PLAN INCLUDING INTERIM ANALYSIS 20
10. QUALITATIVE EVALUATION 21
11. REGULATORY ISSUES 22
11.1 CLINICAL TRIAL AUTHORISATION 22
11.2 ETHICS APPROVAL 22
11. 3 CONFIDENTIALITY 22

For peer review only - http://bmjopen.bmj.com/site/about/guidelines.xhtml

11.4 INDEMNITY 23

11.5 SPONSOR 23

11.6 FUNDING 23

11.7 DEVIATIONS AND SERIOUS BREACHES 23

11.8 AUDITS AND INSPECTIONS 24

12. TRIAL MANAGEMENT 24

13. PUBLICATION POLICY 24

14. REFERENCES 25

LIST OF ABBREVIATIONS

AE	Adverse Event
BMQ	Beliefs about Medication Questionnaire
CRF	Case Report Form
CTCAE	Common Terminology Criteria for Adverse Events
DI	Digital Intervention
DMEC	Data Monitoring and Ethics Committee
EQ-5D	EuroQol (health-related quality of life)
HCP	Healthcare Practitioner
ICH GCP	International Conference on Harmonisation of Good Clinical Practice
IMP	Investigational Medicinal Product
MARS	Medication Adherence Report
MBM	My Breathing Matters
NIHR	National Institute for Health Research
PCRN	Primary Care Research Network
PGfAR	Programme Grants for Applied Research
QIPP	Quality, Innovation, Productivity and Prevention
R&D	Research & Development
REC	Research Ethics Committee
SAE	Serious Adverse Event
SSI	Site Specific Information
TMG	Trial Management Group
TSC	Trial Steering Committee

KEYWORDS

Asthma; Self-management; Digital intervention

For peer review only - http://bmjopen.bmj.com/site/about/guidelines.xhtml

TRIAL SYNOPSIS

Title:	My Breathing Matters trial protocol
Sponsor:	Southampton University
Sponsor Ref Number:	TBC
Funder:	NIHR PGfAR
Trial Phase:	Complex intervention phase 1
Indication:	Asthma
Primary Objective:	1. To assess the feasibility and acceptability of a trial of My Breathing Matters (MBM) (a digital intervention designed to improve quality-of-life outcomes for people with asthma).
Secondary Objectives:	1. To assess feasibility of trial procedures including: clinical research governance, recruitment strategy, trial documents (e.g. PIS), eligibility criteria, consent/withdrawal, randomisation & blinding.<br>2. To assess feasibility and acceptability of MBM intervention including: usage & engagement, adherence and completion, fidelity of providers, participant retention.<br>3. To assess feasibility of data collection and analysis procedures, and look at data quality, management of trial data and estimates of effect size across trial outcome measures (see primary/secondary endpoints) to inform sample size calculations for a larger phase 3 RCT.
Tertiary Objectives	1. To examine intervention users' usage, engagement and possible mediators of behaviour change to inform future intervention design for well-powered usage and process analyses.
Trial Design:	Individually randomised controlled trial (assessor blinded)
Sample size : (split by treatment group)	80 participants (40 per arm)
Inclusion Criteria:	1. Age 18+ years.<br>2. Physician diagnosed asthma in medical record<br>3. [illegible]anti-asthma medication prescription in the previous year (determined from the physician prescribing records)<br>4. Impaired asthma-related health status (Asthma Quality of Life Questionnaire score of <5.5)<br>5. Informed consent<br>6. Able to access the internet and understand written english.
Exclusion Criteria:	1. Asthma judged at the baseline assessment to be dangerously unstable and in need of urgent medical review (if unstable asthma is found, the patient will be referred back to usual primary care clinician for review)<br>2. Terminal disease or other condition which in the opinion of the family doctor makes them inappropriate to take part<br>3. Diagnosed with 'difficult asthma' as defined by BTS.<br>4. Documented diagnosis of Chronic Obstructive Pulmonary Disease (COPD)

For peer review only - http://bmjopen.bmj.com/site/about/guidelines.xhtml

	5. Household member already enrolled on the study
Intervention	My Breathing Matters
Control Group:	Usual care
Primary Endpoints:	Feasibility and acceptability of intervention and trial procedures, including:<br>1. Uptake<br>2. Adherence<br>3. Completion rates
Secondary Endpoints:	Feasibility of measuring (and estimates of effect size to perform sample size calculations) in the following trial measures:<br>1. Asthma-specific Quality of life (AQLQ; short version)<br>2. Asthma control (ACQ)<br>3. Lung function (FEV_1, FEV_1/FVC, PEFR)<br>4. Quality of life (EQ5D, ICECAP-A)<br>5. Anxiety and depression (HADS)<br>6. Patient enablement (PEI)<br>7. Patient burden – time and costs.<br>8. Health resource use (professional contacts, referrals, prescriptions)<br>9. Adherence to recommendations
Tertiary Endpoints	Measurement of patient intervention group only:<br>1. Lifestyle change choice<br>2. Intervention usage and progress<br>3. Engagement with program and reasons for such engagement.
Follow up duration	12 months
Total Number of Sites :	Pilot: 4-6 sites

For peer review only

For peer review only - http://bmjopen.bmj.com/site/about/guidelines.xhtml

Participant flow diagram

Practices identified by the PCRN:

- 6 practices diversely sampled by size, rural-urban location and Index of Multiple Deprivation

↓

Patient identification:

- Practice database searches and letter of invitation and PIS sent by practice
- Opportunistic recruitment via consultations with GP or practice nurse
- Study advertisement posters within practices
- Screen approximately 10-20 patients per practice (estimated mail-out response rate)

↓

Baseline consultation with Research nurse:

- Consent, height, weight (BMI)
- Confirm asthma diagnosis via practice if recruited via poster.
- Questionnaire measures (paper copy)
- Patients given instructions for Lifeguide registration
- Enrol approx. 5-10 patients per practice (total N = 60)
- Randomisation (1:1).

Online registration and randomisation:

- Standardised advice given to all patients (Asthma UK information leaflet)
- Management decisions made by usual clinicians in GP practice as per usual care.

↓

Intervention group (DI ; N = 40)

Patient has access to My Breathing Matters

- Online training in appropriate asthma medication use and access to self-management support
- Online breathing retraining
- Access to online lifestyle modification programs within intervention (physical activity, weight reduction, smoking cessation)
- Resource use monitored.
- Qualitative interviews to discuss experience of intervention

Usual Care (N=40)

- Resource use monitored.

↓

Questionnaire assessment (3 months)

- To be completed via post; second pack will be sent out after 2 weeks if no response; AQLQ completed over the phone with blinded researcher if still outstanding after a further 2 weeks.

↓

Outcome assessment with research nurse (12 months):

- Quality of life and clinical measures (measured by blinded research nurse)
- AQLQ to be completed over phone or via postal pack if not willing to attend)

For peer review only - http://bmjopen.bmj.com/site/about/guidelines.xhtml

SCHEDULE OF OBSERVATIONS AND PROCEDURES

	Time point			
Measure	Baseline/ Screening	3-month follow up	Followup	Within the MBM programme
**Month**	0	3	12	(0-12)
Patient socio-demographic measures	X			
Clinical measures				
Weight (kg)	X	X	X	
Height (cm)	X			
Medication changes (prescriptions issued)			X (NR)	
Consultations			X (NR)	
Patient self-report measures				
Patient Enablement Instrument	X	X	X	
Asthma-specific Quality of life	X	X	X	
Asthma control	X	X	X	
Anxiety & depression	X	X	X	
Quality of life	x	X	X	
Medication adherence	X	X	X	
Lung function	X		X	
Medication use	X		X	
Patient objectively recorded measures				
Website usage				X*
Usage of asthma action plan (PAAP)				X*
Usage of asthma review pages				X*
Booked asthma review				X*
Usage of '4-week medication challenge'				X*
Usage of medication information				X*
Usage of breathing retraining challenge				X*
Usage of friends & family section				X*
Usage of stress reduction				X*
Choice of lifestyle changes				X*
Reported progress on lifestyle change (e.g. weight change)				X*
Economic measures				
Patient quality of life	X	X	X	
Patient costs	X	X	X	
Patient time and resource use			X (NR)	
Qualitative analysis				
Patient experience and views of the DI				X

Key:
NR = Notes review
*My Breathing Matters arm only – measured via Lifeguide website
1 This will be measured directly before and after the My Breathing Matters training completion

For peer review only

For peer review only - http://bmjopen.bmj.com/site/about/guidelines.xhtml

1. INTRODUCTION

1.1 BACKGROUND

The UK has one of the highest prevalence's of asthma in the world; nearly 6% of the UK population have asthma, comprising 5.4 million people, most of whom are managed in primary care[1]. Hospital admission and mortality rates for asthma showed improvements in the last decades of the last century, but these improvements have stalled since the millennium. Premature mortality from asthma was 1.5 times as high in the UK as in the rest of the EU in 2008, with around 1000 to 1200 deaths a year recorded since 2000. It is estimated that 90% of deaths are associated with preventable factors. Asthma is associated with high numbers of admissions and Emergency Department attendances, and it is estimated that 70% of these could have been prevented by appropriate early intervention and self-management[1]. Surveys of asthma symptoms and health status impairment continue to show that sub-optimal control is common and that at any given time the majority of asthmatics in the UK suffer potentially avoidable symptoms and quality of life impairment[2].

Although the UK leads the world in providing guidelines for asthma management, these have been poorly implemented and people with asthma do not receive evidence-based interventions, particularly individual action plans, which are known to impact positively on outcomes[3]. Patient education and proactive self-management have been convincingly shown to improve clinical outcomes in asthma[3] and have been advocated in guidelines for 20 years[4]. People with asthma without a management plan are four times more likely to have an asthma attack needing emergency care in hospital[5]. However, a representative (Ipsos-MORI) survey carried out by Asthma UK in 2010[6] suggested that only a quarter of people with asthma in the UK have a self-management plan. Self-management in asthma can also encompass non-pharmacological interventions to improve control and empower the patient, such as breathing exercises or lifestyle changes such as smoking cessation and weight reduction (since smoking and obesity are associated with worse prognosis in asthma[7]).

1.2 RATIONALE AND RISK BENEFITS FOR CURRENT TRIAL

Increasingly widespread access to the internet and mobile phones [8,9] means that healthcare Digital Interventions (DIs) are accessible to the majority of patients, and can be used to provide information and support at any time the patient needs it [10]. DIs can empower patients by providing better access to personalised information, and support for active involvement in treatment and self-management [10]. A large meta-analysis found a small but significant positive effect of DIs on health-related behaviours [11], whilst a Cochrane review found evidence that computer-based health interventions for those with chronic health conditions significantly improved knowledge, health behaviours and clinical outcomes [12]. DIs have the potential to make significant savings by automating routine aspects of patient education, monitoring and support, freeing up health professional resources for when patients most need them[13]. These savings can play an essential part in meeting the NHS QIPP agenda to achieve increased efficiency gains despite the growing demand created by an expanding and ageing population.

For peer review only - http://bmjopen.bmj.com/site/about/guidelines.xhtml

There is accumulating evidence that DIs can deliver better and more efficient healthcare in the context of asthma. A recent systematic review by our group found that self-management DIs could improve asthma control and reduce asthma-related quality of life impairment but called for larger, more robust trials[14]. We identified several existing self-management DIs for asthma, but half of these were for children, and many were not in English. While there are numerous commercial DIs for asthma, only one has been evaluated[15]. The RAISIN pilot trial demonstrated that self-management interventions could be effective at improving quality of life and asthma control, with improvements to 'reach' and response rate by catering to patients with mild asthma but impaired quality of life through non-pharmacological means [16,17]. A Danish trial compared GP and specialist care with web-based self-monitoring with automated feedback and a stepped care medication plan (with GP advice when required)[15]. After six months those allocated to the web-based self-monitoring had greater improvement in symptoms, quality of life and lung function. The SMASHING trial in the Netherlands[18] compared usual care with web-based educational resources, self-monitoring and automated feedback on medication titration, plus some group and email nurse support. At one year the intervention group had better quality of life and lung function and more symptom free days, at no extra cost[19].

2. TRIAL OBJECTIVES

The primary aim of the My Breathing Matters trial is to assess the feasibility, acceptability, effectiveness and cost-effectiveness of a DI in primary care for the self-management of asthma, in comparison to usual care (with provision of standard patient information materials produced by the charity Asthma UK).

Main research question:

1. To assess the feasibility and acceptability of a trial of My Breathing Matters, an intervention designed to assess improvements in clinical outcomes (e.g. quality of life, health resource use, lung function) of people with asthma.

Secondary research questions

1. To assess feasibility of trial procedures including: clinical research governance, recruitment strategy, trial documents (e.g. PIS), eligibility criteria, consent/withdrawal, randomisation and blinding.
2. To assess feasibility and acceptability of MBM intervention including: usage and engagement, adherence and completion, fidelity of providers.
3. To assess feasibility of data analysis, including data collection, data quality, management of trial data and estimates of effect size across trial outcome measures (see primary/secondary endpoints) to inform sample size calculations for a larger phase 3 RCT.

Tertiary research questions

1. To examine intervention usage, progress and engagement to inform well-powered usage analysis in larger trial

3. TRIAL DESIGN

For peer review only - http://bmjopen.bmj.com/site/about/guidelines.xhtml

My Breathing Matters is a digital intervention for the self-management of asthma, consisting of pharmacological support, (advice about asthma reviews and personal asthma action plans, information about medication and side effects) and non-pharmacological components (stress reduction, online versions of breathing retraining courses shown to be acceptable and feasible[20]) and optional user-selected lifestyle modifications. 80 participants will be randomised either to the My Breathing Matters programme or to a control group receiving usual care with provision of an Asthma UK information leaflet. This trial will be coordinated from UK facilitated by the PCRN's in these areas with researchers and research nurses employed at each centre.

The MBM study will comprise:

1) **Feasibility trial**: 80 participants (40 per arm) will be recruited from practices to confirm the acceptability and feasibility of the intervention, full trial protocol and study procedures.
2) **Qualitative analysis** will also be embedded in to the My Breathing Matters study.

3.1 TRIAL OUTCOME MEASURES

The primary outcome of the trial will be the feasibility and acceptability of a trial of My Breathing Matters, an intervention designed to improve clinical outcomes (e.g. symptom control, quality of life) of people with asthma.

Secondary outcomes will be to assess the feasibility of measuring (and estimates of effect size to generate hypotheses and perform sample size calculations with which to test them) in the following trial measures:

1. Asthma-specific Quality of life (AQLQ; short version)
2. Asthma control (ACQ)
3. Lung function (FEV_1, FEV_1/FVC, PEFR)
4. Quality of life (EQ5D)
5. Anxiety and depression (HADS)
6. Patient enablement (PEI)
7. Perceived support
8. Costs of equipment and drugs
9. Health resource use (professional contacts, referrals, prescriptions)
10. Adherence to recommendations
11. Engagement with program and reasons for such engagement

3-month questionnaire packs will be mailed out with a freepost return envelope. Baseline and 12-month follow up questionnaires will be taken using paper questionnaire packs and clinical measures will be taken by a research nurse.

For 3 month and 12-month measures, a second pack will be sent out after 2 weeks if no response; followed by questionnaire measures (AQLQ) completed over the phone if still outstanding after a further 2 weeks.

<u>Further detail regarding clinical measures:</u>

- Lung function will be measured using spirometry by an appropriately trained research nurse. Measures of lung function will be: FEV_1 (forced expiratory volume in 1 second), FEV_1/FVC (ratio of FEV_1 to forced vital capacity), PEFR (peak expiratory flow rate).

For peer review only - http://bmjopen.bmj.com/site/about/guidelines.xhtml

- Medication prescriptions, any changes and support provision (consultations) will be identified from patient notes reviews after completion of the 12-month follow-up. Medication use will be converted into defined daily doses of medication.

Further detail regarding patient reported measures:

- Asthma-specific quality of life (AQLQ) at 12-month follow-up
- Asthma symptoms will be measured using the Asthma Control Questionnaire (ACQ)
- Anxiety and depression will be measured using the Hospital Anxiety and Depression inventory (HADS)
- Patient enablement (PEI): will be measured using the Patient Enablement Instrument [31].
- Patient medication adherence: all participants will complete the MARS as a measure of medication adherence [2].

In the event that the results from any of the questionnaires are of concern (e.g. demonstrate significant anxiety or depression), the research team will inform the participant of their scores and the normal range of scores, and suggest that they may wish to discuss this result with their GP.

Economic measures:
- Health resource use (professional contacts, referrals, prescriptions)
- Patient time/burden needed to take part in intervention
- Health-related quality of life: the EuroQol (EQ-5D 5L) provides a measure of quality of life for economic analysis[5] and the ICEACAP-A measures non-health-specific quality of life in adults[21].
- Patient costs (e.g. additional costs incurred if patient engages in extra activities beyond intervention, such as gym membership)

Tertiary outcomes (intervention group only) will include:

- Lifestyle change choice, usage and progress
- Adherence to recommendations
- Engagement with program and reasons for such engagement

All measured over the 12 months of the study.
Assessed at baseline, after 3 months and after 12 months of online training.

Socio-demographic data
- Participants' age, gender, internet experience, disease status (time diagnosed), smoking habits, education level, and social deprivation indices based on postcode will be recorded at baseline

4. CENTRE/PARTICIPANT SELECTION AND ENROLLMENT OF PARTICIPANTS

4.1 CENTRE SELECTION

For peer review only - http://bmjopen.bmj.com/site/about/guidelines.xhtml

Eligible participants will be identified from around 6 general practices located by primary care research networks (PCRN); Practices will be selected in order to confirm feasibility for a wider trial. Principal Investigators will be identified at the regional level, with lead GPs nominated at each site.

The following documents must be in place and copies sent to the MBM Clinical Trial Coordinator (CTC) (see contact details on page 2):

- The approval letter from the relevant R&D Department, following submission of the Site Specific Information (SSI) form (where required)
- A signed Study Agreement (PI and sponsor signature)
- Completed Signature List and Roles and Responsibilities document
- Completed contacts list of all site personnel working on the Study

Upon receipt of all the above documents, the MBM CTC will send a confirmation letter to the lead GP. This letter must be filed in each centre's Site File. Along with this confirmation letter, the practice should receive their trial supplies and a study pack holding all the documents required to recruit a patient into the MBM Trial.

4.2 SCREENING AND PRE-REGISTRATION

Screening

- Electronic database searches will be conducted, and records screened by general practitioners to remove participants who meet exclusion criteria. The latest version of the participant information sheets along with the consent and screening questionnaire (including a freepost response envelope) will be posted with a letter inviting the patient to take part in the study;
- Eligible patients may also be identified and referred opportunistically during routine consultations with the practice nurse or GP.
- Study advertisements will be displayed in participating practices;
    - Patients responding to the study advertisements will phone the research team who will conduct a minimal screening of participants by telephone using a standard set of questions (included as a supporting document) to establish whether they meet the basic inclusion criteria, e.g. diagnosis of asthma, access to internet. The research team will then send copies of patient study materials (consent form and questionnaire) to confirm eligibility.

Patients who do not respond within one month of the practice invite will be followed up by telephone. This procedure will be explicitly outlined in the invite letter sent from the practice. In addition, all invited participants will be provided with a response form to return should they wish to decline to take part in the trial. This will ask for basic demographic information and their reasons for declining, including an option not to give a reason if they prefer not to (see patient opt-out).

4.3 INCLUSION CRITERIA

1. Physician diagnosed asthma in medical record (confirmed via practice)
2. ≥ anti-asthma medication prescription in the previous year (determined from the physician prescribing records)

For peer review only - http://bmjopen.bmj.com/site/about/guidelines.xhtml

3. Impaired asthma-related health status (Asthma Quality of Life Questionnaire score of <5.5)
4. Informed consent
5. Able to understand English and access internet.

4.4 EXCLUSION CRITERIA

6. Asthma judged at the baseline assessment to be dangerously unstable and in need of urgent medical review (if unstable asthma is found, the patient will be referred back to usual primary care clinician for review)
7. Terminal disease or other condition which in the opinion of the family doctor makes them inappropriate to take part
8. Diagnosed with 'difficult asthma' as defined by BTS.
9. Documented diagnosis of Chronic Obstructive Pulmonary Disease (COPD)
10. Household member already enrolled on the study

4.5 REGISTRATION / RANDOMISATION PROCEDURES

Informed consent

All patients will receive a copy of the consent form in their invitation letter pack, and will be asked to complete it when contacting the research team. All patients will receive a face-to-face baseline consultation with the practice nurse where additional informed consent (see consent form) will be recorded before clinical measures, such as confirmation of asthma diagnosis, are undertaken (see primary outcomes data document).

Randomisation

Patients will be randomised at the practice where if in the intervention group they will be given access to the My Breathing Matters programme, or if in the control group will be given Asthma UK information leaflet. (see section 9.2 for further details of randomisation). Participants in the intervention group will be instructed to sign up to the My Breathing Matters programme upon which they will be notified by email of their involvement in the study. Only one patient per household will be randomised.

4.6 CONSENT

Consent to enter the trial must be sought from each participant only after a full explanation has been given, an information leaflet offered and time allowed for consideration. Signed participant consent will be obtained. The right of the participant to refuse to participate without giving reasons will be respected. After the participant has entered the trial the clinician remains free to give alternative treatment to that specified in the protocol at any stage if he/she feels it is in the participant's best interest, but the reasons for doing so should be recorded. In these cases the participants remain within the trial for the purposes of follow-up and data analysis. All participants are free to withdraw at any time from the protocol intervention without giving reasons and without prejudicing further treatment.

Participants will give informed consent (including explicit consent to access relevant data from the DI and their medical records) before they first log onto MBM. They will be free to withdraw at any time simply by contacting the research team or practice staff

For peer review only - http://bmjopen.bmj.com/site/about/guidelines.xhtml

4.7 WITHDRAWAL CRITERIA

Patients will be withdrawn from the trial if:

- They are no longer eligible (specialist management of Asthma or illness precluding participation)
- They choose not to continue

All patients who withdraw from the intervention will be asked if they are prepared to attend the final follow-up appointment with the research nurse and/or complete self-report follow-up measures. If they agree, they will be invited according to the follow-up procedure and will have the option to answer follow up questionnaires without using the website.

.

6. INTERVENTION

6.1 INTERVENTION ARM

The **online My Breathing Matters programme** will comprise **three** main components:

1. Intervention components

The MBM programme will use behavioural techniques to improve functional quality of life of primary care patients with asthma, by supporting illness self-management by pharmacological and non-pharmacological means (thereby reducing risk of asthma exacerbation).

The program has three main design objectives: i) to engage people who do not view themselves as having active asthma, ii) to persuade and educate users to implement appropriate pharmacological management and iii) to encourage users to employ non-pharmacological methods of improving QoL. Key features to address these objectives include: i) maintaining positive illness context throughout (i.e. promote health rather than manage illness) and offering a simple, unobtrusive interface to provide optional (and flexible) support only when needed, ii) focusing on persuading/educating users regarding the necessity, efficacy and safety of preventative asthma medication, and facilitating easy completion of an action plan with primary care support, and iii) educating users on benefits and offer psychological methods to improve quality of life (e.g. cognitive behavioural techniques for symptom management), tailored access and address patient concerns about relevant positive lifestyle changes, such as weight-loss if overweight, smoking cessation if current smoker, physical activity if inactive.

Patients in the intervention group will be offered optional support from Asthma UK helpline – as a source of asthma advice and support for those who would like personal contact outside of that offered by MBM or their healthcare team, to support self-monitoring and lifestyle modification. This support would be the same as that offered to anyone who calls (outside of MBM) so the Asthma UK nurse team would not require additional training.

For peer review only - http://bmjopen.bmj.com/site/about/guidelines.xhtml

Motivating and reassuring support messages will be sent by email to all patients in the intervention group every 2 to 4 weeks; these will encourage and reinforce patient adherence to lifestyle changes (where applicable).

6.2 USUAL CARE

All patients will be able to access the Asthma UK information leaflet following randomisation. Patients within the usual care condition will be informed that they should simply follow their usual instructions for asthma management. After the patients have completed the 12-month follow-up they will be given access to the intervention, in line with recommendations from PPI representatives.

7. ADVERSE EVENTS AND REPORTING

7.1 DEFINITIONS

Adverse Event (AE): any untoward medical occurrence in a participant or clinical study participant which does not necessarily have a causal relationship with study treatment or participation.

Serious Adverse Event (SAE) or Serious Adverse Reaction: any untoward medical occurrence or effect that at any dose:

- Results in death
- Is life-threatening – refers to an event in which the participant was at risk of death at the time of the event; it does not refer to an event which hypothetically might have caused death if it were more severe
- Requires hospitalisation, or prolongation of existing hospitalisation
- Results in persistent or significant disability or incapacity
- Is a congenital anomaly or birth defect
- Other important medical events***.

*'life-threatening' in the definition of 'serious' refers to an event in which the patient was at risk of death at the time of the event; it does not refer to an event which hypothetically might have caused death if it were more severe.
**Hospitalisation is defined as an inpatient admission, regardless of length of stay, even if the hospitalisation is a precautionary measure for continued observation. Hospitalisations for a pre-existing condition, including elective procedures that have not worsened, do not constitute an SAE.
***Other important medical events that may not result in death, be life-threatening, or require hospitalisation may be considered a serious adverse event/experience when, based upon appropriate medical judgment, they may jeopardise the participant and may require medical or surgical intervention to prevent one of the outcomes listed in this definition.

Note: It is the responsibility of the PI or designated to grade an event as 'not serious' (AE) or 'serious' (SAE).

For peer review only - http://bmjopen.bmj.com/site/about/guidelines.xhtml

7.2 CAUSALITY

The assignment of the causality to trial procedures of any serious event should be made by the investigator responsible for the care of the participant using the definitions in the table below.

If any doubt about the causality exists the local investigator should inform the trial coordinator who will notify the Chief Investigator. Other clinicians may be asked for advice in these cases.

In the case of discrepant views on causality between the investigator and others, all parties will discuss the case. In the event that no agreement is made, the Ethics Committee will be informed of both points of view.

Relationship	Description
**Unrelated**	There is no evidence of any causal relationship
**Unlikely**	There is little evidence to suggest there is a causal relationship (e.g. the event did not occur within a reasonable time after administration of the trial medication). There is another reasonable explanation for the event (e.g. the participant's clinical condition, other concomitant treatment).
**Possible**	There is some evidence to suggest a causal relationship (e.g. because the event occurs within a reasonable time after administration of the trial medication). However, the influence of other factors may have contributed to the event (e.g. the participant's clinical condition, other concomitant treatments).
**Probable**	There is evidence to suggest a causal relationship and the influence of other factors is unlikely.
**Definitely**	There is clear evidence to suggest a causal relationship and other possible contributing factors can be ruled out.

7.3 REPORTING PROCEDURES

All adverse events should be reported. Depending on the nature of the event the reporting procedures below should be followed. Any questions concerning adverse event reporting should be directed to the SCTU in the first instance.

7.3.1. Pre-existing Conditions

A pre-existing condition should not be reported as an AE unless the condition worsens by at least one CTCAE grade during the trial. The condition, however, must be reported in the pre-treatment section of the CRF, if symptomatic at the time of entry, or under concurrent medical conditions if asymptomatic.

7.3.2. Non serious AEs

All adverse events that may be related to the study will be recorded in the relevant case report form and Adverse Event form and sent to the SCTU within one month of the form being due. As adults on average see their GP approximately 5 times per year for a variety of routine and unscheduled appointments (e.g. for medication review, self-limiting minor illnesses and long-

For peer review only - http://bmjopen.bmj.com/site/about/guidelines.xhtml

term conditions unrelated to asthma), many medical encounters are of no relevance to the study. Events that will be recorded include any judged by the study nurse to be possibly related to the study. In particular, all medical encounters related to the following medical areas or symptoms will be recorded in the Adverse Events form:

Psychological morbidity: any events relating to anxiety, depression or mood disorders
Respiratory morbidity: any events relating to breathing or chest symptoms
Musculoskeletal, Abdominal and chest pain: any events relating to pain in these systems unless known to be associated with an unrelated pre-existing condition.

The study nurses are advised to record any event for which there is uncertainty as to whether it is study related or not, and to discuss with the local PI or CI.

7.3.3 Serious AEs

All SAEs (including those that are expected and related) will be reported within 24 hours of the local site becoming aware of the event. The SAE form asks for nature of event, date of onset, severity, corrective therapies given or action taken, outcome and relatedness (i.e. unrelated, unlikely, possible, probably, definitely). The responsible centre Principal Investigator will assign the relatedness and expectedness of the event. Additional information will be provided as soon as possible if the event has not resolved at the time of reporting.

A flowchart is given below to illustrate reporting procedures:

- GPs or nurses will be asked to notify us via an SAE form if a participant experiences any SAEs.
- The Sponsor and main Research Ethics Committee (REC) will be informed of all related SAEs occurring during the trial according to the following timelines, where day zero is defined as the date the SAE form is initially received:
- Events which are fatal or life-threatening will be reported no later than 7 calendar days after the sponsor is first aware of the reaction. Any additional relevant information must be reported within a further 8 calendar days.
- Events that are non-fatal or non life-threatening will be reported within 15 calendar days of the sponsor first becoming aware of the reaction.
- All Investigators will be informed of all related SAEs occurring throughout the trial. Local Investigators should report any SAEs as required by their Local Research Committee and/or Research and Development Office.

7.3.4 Follow Up and Post-study Serious Adverse Events

The reporting requirement for SAEs affecting participants applies for all events occurring up to the end of the last treatment. All unresolved adverse events should be followed by the local investigator until resolved, the participant is lost to follow-up, or the adverse event is otherwise explained. At the last scheduled visit, the investigator should instruct each participant to report any subsequent event(s) that the participant, or the participant's general practitioner, believes might reasonably be related to participation in this study. The investigator should notify the Centre for Applications of Health Psychology of any death or adverse event occurring at any time after a participant has discontinued or terminated study participation that may reasonably be related to this study.

For peer review only - http://bmjopen.bmj.com/site/about/guidelines.xhtml

8. ASSESSMENT AND FOLLOW-UP OF PARTICIPANTS

Measures will be administered for all participants at baseline, 3 and 12 months, unless otherwise stated (see Schedule of Observations and Procedures). Patient-reported outcome measures will be completed online. Non-respondents will receive two email reminders followed by a paper-based copy accompanied by a final telephone follow-up. All website usage (MBM measures) will be recorded automatically in Lifeguide.

An independent research nurse, blind to study allocation will complete the follow-up assessments. All patients (including withdrawn participants who have consented to follow-up appointments) will follow the procedure outlined below:

- Approximately three weeks before the follow-up appointment is due, the patient will be sent a letter by the research team encouraging them to arrange an appointment with the research nurse. Appointments may be scheduled to take place in the patients' home or usual GP practice.
- If the patient does not contact the research team to arrange an appointment within two weeks of the letter being sent the patient will be followed up by telephone.
- The research team or research nurse will send confirmation of the follow-up appointment to the patient by telephone, text, email or letter (dependent on patient preference).
- If the patient indicates that they would not be willing to complete a follow-up, no further contact will be made with the patient regarding the follow-up appointment.

8.1 DEFINITION OF END OF TRIAL

The end of the trial will be defined as date of entry of final data into database.

9. QUANTITATIVE EVALUATION

9.1 SAMPLE SIZE

A minimum of 40 participants from two centres will be recruited to each condition during the study.

9.2 RANDOMISATION

Participants will be randomised in a 1:1 ratio to receive either usual care (control) or MBM programme with optional support (intervention). The randomisation will be stratified block randomisation by the average value of AQLQ scores in the BREATHE Trial (Thomas et al).

Remote allocation will maintain allocation concealment from both the participant and the research nurse prior to allocation, however the outcome assessor will be blinded to participant allocation.

For peer review only - http://bmjopen.bmj.com/site/about/guidelines.xhtml

9.3 STATISTICAL PLAN INCLUDING INTERIM ANALYSIS

A detailed statistical plan will be developed prior to data lock. No interim analysis is planned. Data and all appropriate documentation will be stored for a minimum of 10 years after the completion of the trial, including the follow-up period, in a secure location at Southampton University.

Descriptive statistics of outcome variables will be calculated in order to inform a future randomized controlled trial, including examining levels of missing data, attrition rate, retention and completion. Statistical comparisons between and within intervention and control groups will be performed to estimate variability in primary and secondary outcomes within a linear regression framework. Further exploratory analysis of patient engagement, intervention usage and possible mediators of behaviour change will be performed.

10. QUALITATIVE EVALUATION

10.1 Qualitative Evaluation

The qualitative study will be undertaken both alongside participation and after participants finish using MBM. Qualitative interviews will seek to provide an in-depth understanding of the perspective of patients, to inform intervention and trial acceptability and to generate hypotheses about intervention mechanisms of action that can be tested in a larger trial.

Participants and sampling

Patients will be asked whether they give permission to take part in an interview at a later point in the study. Purposive sampling will be used to select patients from the intervention group to allow for a wide range of views and experiences of the MBM programme. It is anticipated that 20 patients will be interviewed during and following the study, and patients will be selected for interviews until saturation is reached.

Interviews and qualitative analysis

Factors that may facilitate or diminish the acceptability of the MBM programme, and adherence to implementation will be explored across patient and health care professional interviews. Interviews will be conducted from after the 3-month assessment. Open-ended questions will be used to elicit user perspectives and experiences of the intervention, allowing participants to freely describe their experiences and views in their own way and to focus on whatever is most salient to them.

Interviews will be audio-recorded and fully transcribed. The findings will be used to inform any modifications needed to the MBM programme or the trial procedures for a potential future full RCT.

The transcriptions will be anonymised (identifiable data removed) and participants' transcripts will be given participant numbers so that they can be easily discussed between team members whilst protecting participants' identities. To ensure that we remain open to and grounded in users' perspectives we will carry out inductive thematic analysis[22] of all textual data,

For peer review only - http://bmjopen.bmj.com/site/about/guidelines.xhtml
For peer review only

triangulated where appropriate with quantitative self-report measures and web usage data, and constant comparison and discussion among team members to reach inter-rater agreement on themes and interpretations.

11. REGULATORY ISSUES

11.1 CLINICAL TRIAL AUTHORISATION

This trial does NOT involve the testing of any Investigational Medicinal Products (IMPs) therefore approval from the Medicines and Healthcare products Regulatory Agency is not required.

11.2 ETHICS APPROVAL

The trial protocol will be submitted to a Research Ethics Committee (REC) recognised by the United Kingdom Ethics Committee Authority (UKECA) for review and approval. A favourable opinion must be obtained before commencement of any trial procedures (including recruitment of patients) occurs

All substantial amendments must be approved by the REC responsible for the trial, in additional to approval by NHS R&D. Minor amendments will not require prior approval by the REC.

If the trial is stopped prematurely, it will not be recommended without reference to the REC responsible for the trial.

The outcome of the trial will be reported to the responsible REC within 90 days of completion of the last patient's final trial procedures. In the event of the trial being prematurely terminated a report will be submitted to the REC within 15 days. A summary of the Trial Report will be submitted to the responsible REC within one year of completion of the last participant's final study procedures.

The investigator must ensure that participant's anonymity will be maintained and that their identities are protected from unauthorised parties. On CRFs participants will not be identified by their names, but by an identification code.

11. 3 CONFIDENTIALITY

Initial practice database searchers will be conducted on practice computer systems and subsequent study invitations will be the responsibility of the practice, to maintain the confidentiality of potential participants. Participants' identification data will be required for the registration process, which will be completed with the support provider at the baseline screening appointment.

For peer review only - http://bmjopen.bmj.com/site/about/guidelines.xhtml

Participants will be informed that the research team will have access to their study data, and that personal information such as their name, telephone address and email address will be stored and used by the research team to stay in touch with them throughout the study.

The primary data outcomes measured by the support provider (such as asthma quality of life and current medication) will be stored on an independent, secure server. All other data (secondary self-report measures and intervention data) will be stored on dedicated secure spaces behind a firewall on password protected computer located in secure university buildings at the University of Southampton. The data is backed up daily. Confidentiality of all data entered into the DI will be maintained by following best practice in NHS IT protection and data security systems (e.g. regarding use of https, storing data behind the university firewall etc). Access to the website will be via username and password. The use of strong passwords will be enforced. The communication between the person entering data through a web browser and the server will be through a secure internet connection (HTTPS). The research team will adhere to the Data Protection Act 1998.

Digital records will be stored on a university computer in a password protected file. All data will be linked with the participant's study ID which does not include any personal or identifiable information, such as name or D.O.B. Study IDs will be linked with patient's names, email addresses telephone numbers in a separate, securely stored file.

At the end of the study, data will be anonymised and stored on a password protected computer located in secure university buildings and appropriately backed up. Confidentiality of all data entered into the DI will be maintained by following best practice in NHS IT protection and data security systems.

11.4 INDEMNITY

The sponsor of the trial is University of Southampton. University of Southampton insurance will apply. The lead authors of the protocol are the Chief Investigator (Prof Lucy Yardley), and Prof Mike Thomas, who are both university employees and as such will be covered by the University insurance.

11.5 SPONSOR

University of Southampton is acting as the sponsor for this trial. The My Breathing Matters coordinating team has been delegated duties by the Sponsor relating to: submissions to regulatory authorities and GCP.

11.6 FUNDING

NIHR PGfAR are funding this trial (RP-PG-1211-20001).

11.7 DEVIATIONS AND SERIOUS BREACHES

Any trial protocol deviations/violations and breaches of Good Clinical Practice occurring at sites should be reported to the MBM trial coordinator and the local R&D Office immediately.

For peer review only - http://bmjopen.bmj.com/site/about/guidelines.xhtml

The MBM trial coordinator will then advise of and/or undertake any corrective and preventative actions as required.

All serious protocol deviations/violations and serious breaches of Good Clinical Practice and /or the trial protocol will immediately be reported to the regulatory authorities and other organisations, as required in the Medicines for Human Use (Clinical Trials) Regulations 2004, as amended.

11.8 AUDITS AND INSPECTIONS

The trial may be participant to inspection and audit by University of Southampton, under their remit as sponsor, the trial coordinating centre as the Sponsor's delegate and other regulatory bodies to ensure adherence to ICH GCP, Research Governance Framework for Health and Social Care, applicable contracts/agreements and national regulations.

12. TRIAL MANAGEMENT

The Trial Management Group (TMG) is responsible for overseeing progress of the trial. The day-to-day management of the trial will be co-ordinated through the trial coordinating centre and oversight will be maintained by the Trial Steering Committee, with an experienced independent chair and representation of patient representatives, Asthma UK and Blood Pressure UK.

The Steering Committee will meet twice a year throughout the programme to provide strategic guidance and independent monitoring of progress and professional conduct. We will encourage in person attendance at all these meetings where possible, but will also provide for attendance by teleconference when necessary, and will circulate papers and minutes before and after meetings for communication with those who cannot attend for any reason.

13. PUBLICATION POLICY

All publications and presentations relating to the trial will be authorised by the Trial Management Group and will follow an agreed publication policy. Dissemination of our work will be via multiple pathways:
a) to the scientific community through presentation at national & international conferences and regular publication in highly cited and open access peer reviewed journals.
b) to clinical and academic colleagues via professional societies: links with the following societies will be exploited to raise the profile of this work: Royal College of GPs and Physicians, Society of Behavioural Medicine, British Sociological Society Medical Sub-group.
c) to patients via patient groups: We will work with Asthma UK to disseminate our results.
d) to participants: All participants will be sent an accessible summary of the findings from the study that they took part in within six months of study completion.
e) to relevant NHS organisations and healthcare providers (e.g. Clinical Commissioning Groups, NHS Choices, UCL partners).

For peer review only - http://bmjopen.bmj.com/site/about/guidelines.xhtml

f) to the public via local and national media: we will use regular press releases linked to dissemination events to ensure a high level awareness of our work in the media.
g) to all stakeholders via a dedicated website and through interactive workshops (with health professionals, patient groups, IT providers, commissioners, policy-makers, researchers).

14. REFERENCES

1. Department of Health. An outcomes strategy for people with chronic obstructive pulmonary disease (COPD) and asthma in England. 2011.
2. Demoly P, Gueron B, Annunziata K, Adamek L, Walters RD. Update on asthma control in five European countries: results of a 2008 survey. *European Respiratory Review* 2010;19:150-157.
3. Gibson PG, Powell H, Coughlan J et al. Self-management education and regular practitioner review for adults with asthma. *Cochrane Database of Systematic Reviews* 2002;3.
4. British Thoracic Society / Scottish Intercollegiate Guideline Network. British Guideline on the Management of Asthma. 2012. 22-8-0012.
5. Adams R, Smith B, Ruffin R. Factors associated with hospital admissions and repeat emergency dept visits for adults with asthma . *Thorax* 2000;55:566-573.
6. Asthma UK. National Asthma Panel 2010. 2010. London, Asthma UK.
7. Thomas M, Price D. Impact of comorbidities on asthma. *Expert Reviews in Clinical Immunology* 2008;4:731-742.
8. Ogedegbe G. Barriers to optimal hypertension control. *Journal of Clinical Hypertension* 2008;10:644-666.
9. Guthrie B, Inkster M, Fahey T. Tackling therapeutic inertia: role of treatment data in quality indicators. *Br Med J* 2007;335:542-544.
10. Ogedegbe G, Schoenthaler A. A systematic review of the effects of home blood pressure monitoring on medication adherence. *Journal of Clinical Hypertension* 2006;8:174-180.
11. Bosworth HB, Olsen MK, Grubber JM et al. Two Self-management Interventions to Improve Hypertension Control: A Randomized Trial. *Annals of Internal Medicine* 2009;151:687-695.
12. Agarwal R, Bills JE, Hecht TJW, Light RP. Role of home blood pressure monitoring in overcoming therapeutic inertia and improving hypertension control: a systematic review and meta-analysis. *Hypertension* 2011;57:29-38.
13. Abudagga A, Resnick HE, Alwan M. Impact of blood pressure monitoring on hypertension outcomes: a literature review. *Telemedicine and e-Health* 2010;16:830-837.
14. McLean G, Murray E, Band R et al. Interactive digital interventions to promote self-management in adults with asthma: systematic review and meta-analysis. *BMC Pulmonary Medicine* 2016:16:83.
15. Rasmussen LMPK, Nolte H, Backer V. Internet-based monitoring of asthma: A long-term, randomized clinical study of 300 asthmatic subjects. *Journal of Allergy and Clinical Immunology* 2005;115:1137-1142.
16. Morrison D, Wyke S, Saunderson K et al. Findings from a pilot randomised trial of an Asthma Internet Self-management Intervention *BMJ Open* 2016:6:e009254.
17. Morrison D, Mair F, Chaudhuri R et al. Details of development of the resource for adults with asthma in the RAISIN (randomized trial of an asthma internet self-management intervention) study. *BMC Medical Informatics & Decision Making* 2015; 15:57.
18. van der Meer V, Bakker MJ, van den Hout WB et al. Internet-based self-management plus education compared with usual care in asthma: A randomized trial. *Annals of Internal Medicine* 2009;151:110-120.
19. van der Meer V, van den Hout WB, Bakker MJ et al. Cost-Effectiveness of Internet-Based Self-Management Compared with Usual Care in Asthma. *PLoS ONE* 2011;6:e27108.

For peer review only - http://bmjopen.bmj.com/site/about/guidelines.xhtml

20. Yardley L, Ainsworth B, Arden Close E et al. The person-based approach to enhanceing the acceptability and feasibility of interventions, *Pilot & Feasibility Studies, 2015 : 1.*
21. Al-Janabi H, Flynn T, Coast J. (2012) Development of a self-report measure of capability wellbeing for adults: the ICECAP-A*. Quality of Life Research* 21: 167-176
22. Joffe H, Yardley L. Content and thematic analysis. In: Marks D, Yardley L, eds. *Research Methods for Clinical and Health Psychology*. London: Sage; 2004;56-68.

For peer review only

For peer review only - http://bmjopen.bmj.com/site/about/guidelines.xhtml

15. Trial Timeline

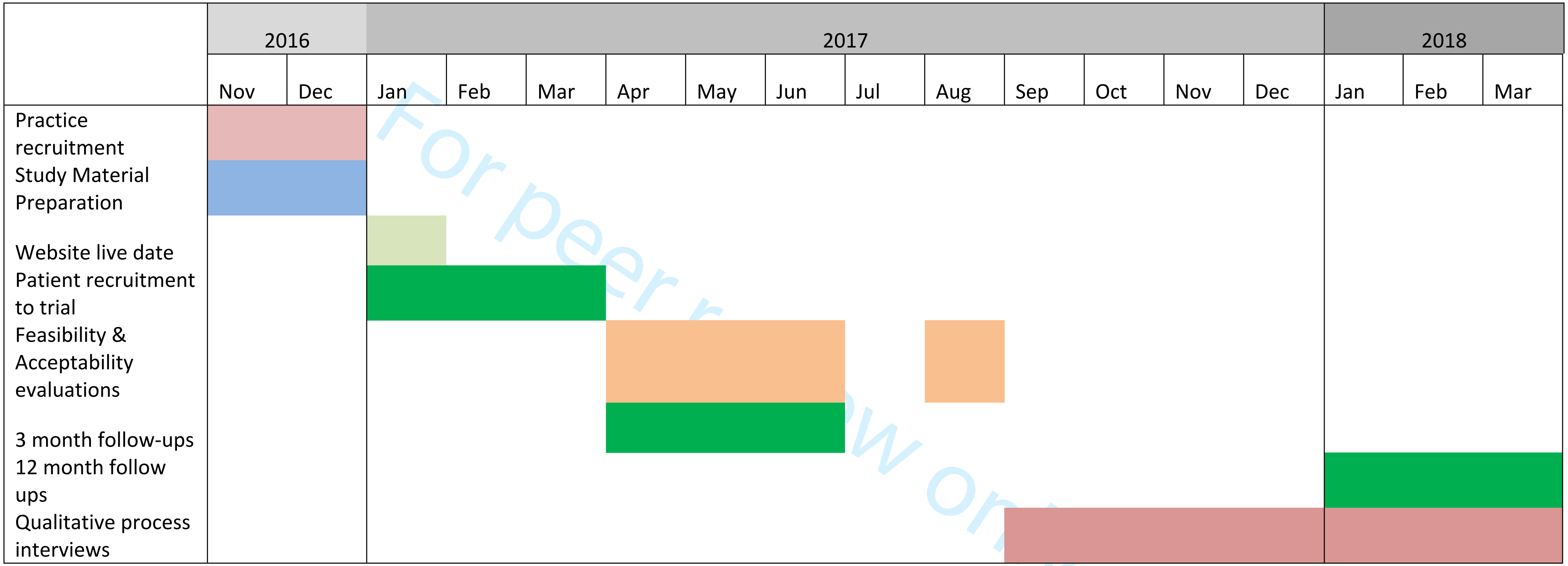

For peer review only

For peer review only - http://bmjopen.bmj.com/site/about/guidelines.xhtml

CONSORT 2010 checklist of information to include when reporting a pilot or feasibility trial*

Section/Topic	Item No	Checklist item	Reported on page No
**Title and abstract**			
	1a	Identification as a pilot or feasibility randomised trial in the title	1
	1b	Structured summary of pilot trial design, methods, results, and conclusions (for specific guidance see CONSORT abstract extension for pilot trials)	2
**Introduction**			
Background and objectives	2a	Scientific background and explanation of rationale for future definitive trial, and reasons for randomised pilot trial	6
	2b	Specific objectives or research questions for pilot trial	6
**Methods**			
Trial design	3a	Description of pilot trial design (such as parallel, factorial) including allocation ratio	7
	3b	Important changes to methods after pilot trial commencement (such as eligibility criteria), with reasons	-
Participants	4a	Eligibility criteria for participants	7
	4b	Settings and locations where the data were collected	7
	4c	How participants were identified and consented	7
Interventions	5	The interventions for each group with sufficient details to allow replication, including how and when they were actually administered	8
Outcomes	6a	Completely defined prespecified assessments or measurements to address each pilot trial objective specified in 2b, including how and when they were assessed	10
	6b	Any changes to pilot trial assessments or measurements after the pilot trial commenced, with reasons	-
	6c	If applicable, prespecified criteria used to judge whether, or how, to proceed with future definitive trial	-
Sample size	7a	Rationale for numbers in the pilot trial	8
	7b	When applicable, explanation of any interim analyses and stopping guidelines	-
Randomisation:			
Sequence generation	8a	Method used to generate the random allocation sequence	8
	8b	Type of randomisation(s); details of any restriction (such as blocking and block size)	8
Allocation concealment mechanism	9	Mechanism used to implement the random allocation sequence (such as sequentially numbered containers), describing any steps taken to conceal the sequence until interventions were assigned	8

Implementation	10	Who generated the random allocation sequence, who enrolled participants, and who assigned participants to interventions	8
Blinding	11a	If done, who was blinded after assignment to interventions (for example, participants, care providers, those assessing outcomes) and how	8
	11b	If relevant, description of the similarity of interventions	8
Statistical methods	12	Methods used to address each pilot trial objective whether qualitative or quantitative	13
**Results**			
Participant flow (a diagram is strongly recommended)	13a	For each group, the numbers of participants who were approached and/or assessed for eligibility, randomly assigned, received intended treatment, and were assessed for each objective	14
	13b	For each group, losses and exclusions after randomisation, together with reasons	14
Recruitment	14a	Dates defining the periods of recruitment and follow-up	14
	14b	Why the pilot trial ended or was stopped	-
Baseline data	15	A table showing baseline demographic and clinical characteristics for each group	Table 1
Numbers analysed	16	For each objective, number of participants (denominator) included in each analysis. If relevant, these numbers should be by randomised group	Table 2
Outcomes and estimation	17	For each objective, results including expressions of uncertainty (such as 95% confidence interval) for any estimates. If relevant, these results should be by randomised group	18
Ancillary analyses	18	Results of any other analyses performed that could be used to inform the future definitive trial	-
Harms	19	All important harms or unintended effects in each group (for specific guidance see CONSORT for harms)	19
	19a	If relevant, other important unintended consequences	-
**Discussion**			
Limitations	20	Pilot trial limitations, addressing sources of potential bias and remaining uncertainty about feasibility	24
Generalisability	21	Generalisability (applicability) of pilot trial methods and findings to future definitive trial and other studies	24
Interpretation	22	Interpretation consistent with pilot trial objectives and findings, balancing potential benefits and harms, and considering other relevant evidence	24
	22a	Implications for progression from pilot to future definitive trial, including any proposed amendments	23
**Other information**			
Registration	23	Registration number for pilot trial and name of trial registry	2
Protocol	24	Where the pilot trial protocol can be accessed, if available	4
Funding	25	Sources of funding and other support (such as supply of drugs), role of funders	4
	26	Ethical approval or approval by research review committee, confirmed with reference number	4

For peer review only

Citation: Eldridge SM, Chan CL, Campbell MJ, Bond CM, Hopewell S, Thabane L, et al. CONSORT 2010 statement: extension to randomised pilot and feasibility trials. BMJ. 2016;355.

*We strongly recommend reading this statement in conjunction with the CONSORT 2010, extension to randomised pilot and feasibility trials, Explanation and Elaboration for important clarifications on all the items. If relevant, we also recommend reading CONSORT extensions for cluster randomised trials, non-inferiority and equivalence trials, non-pharmacological treatments, herbal interventions, and pragmatic trials. Additional extensions are forthcoming: for those and for up to date references relevant to this checklist, see www.consort-statement.org.

For peer review only

BMJ Open

A feasibility trial of a digital self-management intervention 'My Breathing Matters' to improve asthma-related quality of life for UK primary care patients with asthma

Journal:	*BMJ Open*
Manuscript ID	bmjopen-2019-032465.R1
Article Type:	Original research
Date Submitted by the Author:	28-Aug-2019
Complete List of Authors:	Ainsworth, Ben; University of Bath, Psychology; University Hospital Southampton NHS Foundation Trust, NIHR Respiratory Biomedical Research Unit<br>Greenwell, Kate; University of Southampton, Psychology<br>Stuart, Beth; University of Southampton, Primary Care and Population Sciences, Faculty of Medicine<br>Raftery, James; University of Southampton, Primary Care and Population Sciences, Faculty of Medicine<br>Mair, Frances; University of Glasgow, Institute of Health and Wellbeing<br>Bruton, Anne; University of Southampton, School of Health Sciences, Faculty of Environmental and Life Sciences<br>Yardley, Lucy; University of Southampton, Psychology; University of Bristol, School of Psychological Sciences<br>Thomas, Mike; University of Southampton, Primary Care and Population Sciences, Faculty of Medicine
**Primary Subject Heading**:	Respiratory medicine
Secondary Subject Heading:	Patient-centred medicine
Keywords:	Asthma < THORACIC MEDICINE, PRIMARY CARE, Digital Medicine, Self-management, Breathing Retraining, Quality of Life

SCHOLARONE™
Manuscripts

BMJ Open

A feasibility trial of a digital self-management intervention 'My Breathing Matters' to improve asthma-related quality of life for UK primary care patients with asthma.

Ben Ainsworth[1,2,*]

Kate Greenwell[3]

Beth Stuart[4]

James Raftery[4]

Frances S Mair[5]

Anne Bruton[6]

Lucy Yardley[3,7]

Mike Thomas[4]

*: corresponding author: b.ainsworth@bath.ac.uk

1: Department of Psychology, Faculty of Humanities and Social Sciences, University of Bath, UK.

2: Respiratory Biomedical Research Unit, University Hospitals Southampton NHS Foundation Trust, Southampton, Hampshire, UK.

3: Centre for Clinical and Community Applications of Health Psychology, School of Psychology, Faculty of Environmental and Life Sciences, University of Southampton, UK.

4. Primary Care and Population Sciences, Faculty of Medicine, University of Southampton, UK.

5. Institute of Health and Wellbeing, University of Glasgow, UK

6. School of Health Sciences, Faculty of Environmental and Life Sciences, University of Southampton, UK

7. School of Psychological Science, University of Bristol, UK.

Keywords: asthma, self-management, quality of life, primary care, breathing retraining, digital

For peer review only

For peer review only - http://bmjopen.bmj.com/site/about/guidelines.xhtml

BMJ Open

Abstract

Objective: To assess the feasibility of a randomised controlled trial (RCT) and acceptability of an asthma self-management digital intervention to improve asthma-specific quality of life in comparison with usual care.

Design and setting: A two arm feasibility RCT conducted across 7 general practices in Wessex, UK.

Participants: Primary care patients with asthma aged 18 years and over, with impaired asthma-specific quality of life and access to the internet.

Interventions: 'My Breathing Matters' (MBM) is a digital asthma self-management intervention designed using theory, evidence and person-based approaches to provide tailored support for both pharmacological and non-pharmacological management of asthma symptoms.

Outcomes: The primary outcome was the feasibility of the trial design, including recruitment, adherence and retention at follow-up (3 and 12 month). Secondary outcomes were the feasibility and effect sizes of specific trial measures including asthma-specific quality of life and asthma control.

Results: Primary outcomes: 88 patients were recruited (target 80). At 3 month follow-up, 2 patients withdrew and 6 did not complete outcome measures. At 12 month, 2 withdrew and 4 did not complete outcome measures. 36/44 patients in the intervention group engaged with MBM (median of 4 logins, range 0-25, IQR 8). Consistent trends were observed to improvements in asthma-related patient reported outcome measures.

Conclusions: This study demonstrated the feasibility and acceptability of a definitive randomised controlled trial that is required to determine the clinical and cost-effectiveness of a digital asthma self-management intervention.

Trial registration number: ISRCTN15698435.

For peer review only - http://bmjopen.bmj.com/site/about/guidelines.xhtml

BMJ Open

Strengths and Limitations of the Study (Summary: up to 5 bullet points)

1. This pragmatic randomised controlled feasibility trial examined ‘My Breathing Matters’ (MBM), a digital asthma self-management intervention that supported both pharmacological and non-pharmacological management of asthma symptoms.
2. MBM was developed using theory, evidence and person-based approaches, and compared to standardised usual care (a booklet) with successful blinding and randomisation.
3. Not all patients engaged with the intervention, and although numeric improvements in patient reported asthma outcomes were larger in the active arm, improvements were observed in both arms.

For peer review only

For peer review only - http://bmjopen.bmj.com/site/about/guidelines.xhtml

Introduction

Asthma prevalence in the UK is amongst the highest in the world at nearly 6% of the UK adult population, comprising 5.4 million people, with most managed in primary care. Although hospital admission and mortality rates for asthma improved from 1970 to 2000, these improvements have since stalled[1]. Surveys of asthma symptoms and health status impairment show that sub-optimal control is common and that the majority of people with asthma in the UK frequently experience potentially avoidable symptoms and quality of life impairment[2].

Proactive self-management of asthma has been convincingly shown to improve clinical outcomes and have been advocated in guidelines for 25 year[3]. Guidelines are not always well implemented[4] and consequently some people with asthma do not receive evidence-based interventions which are known to impact positively on outcomes. Recent large-scale systematic reviews demonstrated that supported asthma self-management can reduce healthcare utilisation and increase asthma control, without increasing healthcare costs[5,6]. For example, people with asthma without a management plan are four times more likely to have an asthma attack needing emergency care in hospital, yet only 44% of people with asthma in the UK report having a self-management plan[7]. Self-management recommendations for asthma have also encompassed non-pharmacological strategies to improve control. These include lifestyle interventions, such as smoking cessation, allergen avoidance, weight reduction in those with obesity, and breathing retraining interventions[8].

Digital interventions (DIs) are increasingly recognised as a possible approach to achieve the aims of supporting chronic diseases such as asthma. DIs can be convenient, easily accessed and may provide cost-effective tools by automating routine aspects of patient education, monitoring and support[9]. There is accumulating evidence that DIs are feasible and may be effective in the context of asthma. The SMASHING trial compared usual care with web-based educational resources, self-monitoring and automated feedback on medication titration, plus some group and email nurse support for patients with asthma. After 12 months the intervention group had

For peer review only - http://bmjopen.bmj.com/site/about/guidelines.xhtml

better quality of life and lung function and more symptom-free days, at no extra cost[10]. The RAISIN pilot trial indicated that self-management interventions that included non-pharmacological (behavioural and psychological) components could be effective at improving quality of life and asthma control, with improvements to 'reach' and response rate, by catering to patients with mild asthma but impaired quality of life[11]. A recent systematic review and meta-analysis indicated that self-management DIs may be able to improve asthma control and reduce asthma-related quality of life impairment [12] however there is limited evidence of benefit for other outcomes and larger confirmatory trials are required.

In the current randomised controlled feasibility trial, we developed and evaluated a digital self-management intervention, that incorporated pharmacological and non-pharmacological self-management support for adults in primary care with impaired asthma-specific quality of life ('My Breathing Matters'; MBM), using evidence, theory and person-based approaches[13] and in line with Medical Research Council (MRC) guidance for developing and evaluating complex interventions[14].

Aim

The aim of the MBM study was to assess the feasibility of a trial to evaluate a digital intervention in primary care to improve quality of life and other clinical outcomes (such as asthma control, health resource use, lung function) of people with asthma, in comparison to usual care (with provision of standard patient information materials produced by the charity Asthma UK).

Research Objectives

1. To assess feasibility of trial procedures including recruitment strategy, eligibility criteria, consent, withdrawal, randomisation and blinding.
2. To assess feasibility of the MBM digital intervention including usage and engagement.
3. To assess feasibility of data analysis, including data collection, data quality and management of trial data across trial endpoint measures to inform sample size calculations for a larger phase 3 randomised controlled trial.

For peer review only - http://bmjopen.bmj.com/site/about/guidelines.xhtml

BMJ Open

Method

Design

We conducted a pragmatic feasibility RCT of the MBM digital intervention in primary care.

Setting

Eligible participants were identified from seven general practices from the Wessex, UK primary care research network to facilitate recruitment of people with varied socio-economic status. To ensure we evaluated the intervention across a spread of socio-economic deprivation, practices were purposively selected to be both rural (N = 4) and urban (N = 3), with mean practice deprivation index of 20.60% (SD 10.5); practice socio-economic deprivation deciles = 2, 4, 4, 5, 8, 10, 10, in which lower deciles indicate more deprivation[15]).

Participants

Patients were included in the trial if they were aged 18 years or more, had physician-diagnosed asthma in their medical record, had received one or more anti-asthma medication prescription in the previous 12 months, had impaired asthma-related health status (Asthma Quality of Life Questionnaire score of less than 5.5 as assessing using a self-completed postal questionnaire), provided informed consent, were able to understand English and had access to the internet.

They were excluded from the trial if 1) their GP considered it inappropriate for them to take part (such as having an additional terminal condition), 2) they were attending a secondary care asthma clinic, or they were receiving either maintenance oral corticosteroids or injected biological treatments to control their asthma, 3) they were diagnosed with COPD, 4) they had a household member already enrolled on the study, or 5) they were judged by the research nurse to have 'unstable asthma' according to clinical assessment and spirometry data at the baseline assessment (in which case they were referred back to their GP), or were diagnosed with 'difficult asthma' defined by British Thoracic Society (BTS).

Recruitment

Electronic searches of the computerised primary care medical record were conducted, and

For peer review only - http://bmjopen.bmj.com/site/about/guidelines.xhtml

records screened by GP to remove ineligible participants. Invitation letters, study information sheets, consent forms screening questionnaires (Mini Asthma Quality of Life Scale; AQLQ[16]) and freepost return envelopes were posted to participants who returned them if they were interested in taking part. Patients who met screening criteria (AQLQ score of less than 5.5) were contacted by research team staff and attended a baseline appointment at their practice with a trained research nurse. Recruitment began in March 2017 and was completed in August 2017.

Sample size

The target for this trial was to recruit 80 patients overall (40 per arm), in order to assess primary feasibility outcomes and to assess intervention engagement and acceptability.

Randomisation and Blinding

After completing outcome measures at their baseline appointment participants were randomised (block randomisation stratified by an average primary care AQLQ score [4.3] taken from a previous trial using the same inclusion criteria[8]). Information packs were given to participants after randomisation with instructions on signing up to MBM (if randomised to intervention group) or just usual care materials (if randomised to control). Research nurses conducting baseline appointments were blinded throughout the study until the final questionnaire which was only delivered to the intervention group.

Interventions

Intervention Group: Usual Care with MBM and Asthma UK booklet.

Patients in the intervention group continued to receive usual care but were also given a code that allowed free unlimited access to MBM. MBM is a digital asthma self-management intervention that supports asthma self-management using both pharmacological and non-pharmacological approaches, developed using the LifeGuide Software[17] and described below according to the TIDieR checklist[18]. A demonstration version of the intervention is available here: http://www.mybreathingmatters.co.uk. After signing up and completing quality-of-life

For peer review only - http://bmjopen.bmj.com/site/about/guidelines.xhtml

For peer review only

related self-monitoring questions, patients were offered tailored advice that directed them towards specific pharmacological or non-pharmacological sections of the online intervention. The pharmacological section provided information on different medication classes and inhalers, the use of personalised asthma action plans (PAAPs), encouraged medication adherence, and gave information to facilitate and inform an effective asthma review with their GP. Pharmacological content was initially based on 'Living Well with Asthma', an asthma self-management intervention that previously demonstrated feasibility for self-management[11,19] and was developed in collaboration with people with asthma and with input from Asthma UK (a national asthma charity). This section was designed to answer common concerns about medication, incorporating a strategy described as 'the 4-week medication challenge' that encouraged participants to realise the benefits of adherence to regular medication, by self-monitoring their symptoms during 4 weeks of continuous inhaler use. The non-pharmacological support included sections on a number of strategies to improve asthma control, such as Breathing Retraining, stress reduction, and additional healthy lifestyle resources (physical activity, weight reduction, hand hygiene and smoking cessation). Optional nurse support was available by Asthma UK who provide a dedicated nurse helpline that was advertised through the intervention.

The intervention was developed using the person-based approach[20] which places patients at the heart of the development process. Evidence from primary mixed methods research (such as [19]) and qualitative and quantitative reviews was used to develop guiding principles. A prototype intervention was piloted using 'think aloud' interview studies in which patients with asthma used prototype versions of the MBM website and provided feedback on intervention acceptability and feasibility as they used it. In 46 interviews with 30 patients (purposively selected across a range of age and gender), the intervention was iteratively modified and updated to address patient feedback until participants indicated no further modification was required, confirming the intervention was as acceptable and engaging as possible (for more details on this process see [21]).

For peer review only - http://bmjopen.bmj.com/site/about/guidelines.xhtml

Due to the digital nature of the intervention, participants could engage with components of the intervention as much or as little as they wished. Tailored advice was offered according to participants' preference to find out more about pharmacological or non-pharmacological self-management techniques (patients selected a check box option of "I'd like to find out more about how my asthma could be helped by i) making the most of my asthma medicine, or ii) 'non-medicine' ways to help my breathing."), with automated reminders whenever patients had not accessed the intervention for several weeks, or when content was made available that they had not previously seen. The intervention was not modified during the study.

Intervention usage was monitored through digital usage metrics (reported below).Non-engagement with the website was not addressed, in line with the pragmatic nature of the feasibility study. Participants were sent one email and received one phone call in which they were offered technical support if they had not logged onto the intervention at all for one month following their baseline appointment.

Control Group: Usual Care with Asthma UK Booklet

To provide 'good quality' usual care to participants allocated to this arm, as well as usual care from their practice, participants were given an Asthma UK booklet 'Live Well with Asthma' at their baseline appointment. The booklet was created by a multidisciplinary team and expert patients, and aimed to provide essential information and advice to enable effective self-management to occur. It is available to anyone via the Asthma UK website[22]. The booklet was provided in hard copy and provided information about asthma symptoms and triggers, medication adherence and usage techniques, PAAPs and support from families. The booklet also advertised the Asthma UK support line. Booklet usage was not monitored.

Outcome Measures

In line with objectives, trial outcomes are reported below as 1) feasibility outcome measures, 2) intervention usage outcome measures, and 3) trial endpoint measures to inform a larger trial.

For peer review only - http://bmjopen.bmj.com/site/about/guidelines.xhtml

BMJ Open

Feasibility outcome measures: Primary outcomes for the trial were descriptive, examining trial design and intervention feasibility and acceptability. These outcomes included patient recruitment, patient withdrawals and follow-up retention.

Internet usage and engagement measures: Usage of the intervention included access to specific intervention components and frequency of engagement with individual components. These data were collected using the LifeGuide software.

Endpoint Measures: It is envisaged that the likely primary outcome measure or measures in a full trial would include validated asthma-specific patient reported outcome measures evaluating symptom control and quality of life, with additional secondary outcomes measuring health resource use, psychological measures and a health economic economic analysis. Data to generate hypotheses (and perform sample size calculations with which to test them) were collected in the following trial endpoints at baseline, 3 month and 12 month:

- Asthma-specific quality of life. Measured using the Mini Asthma Quality of Life Questionnaire (AQLQ[16]), a 15-item 7-point scale in which higher scores represent higher quality of life.
- Asthma control. Measured using the Asthma Control Questionnaire (ACQ[23]) , a 7-item 7-point scale in which higher scores indicate worse asthma control.
- Health-related quality of life. Measured using the EQ-5D-5L[24] in which participants select their functioning level across five dimensions (immobility, self-care, usual activities, pain and discomfort, and anxiety and depression) on a five-point scale, with higher scores indicating greater problems with functioning.
- Health-related capability. Measured using the Icepop Capability measure for adults (ICECAP-A[25]), in which participants select their capability across five dimensions (stability, enjoyment, achievement, attachment and autonomy) on a four-point scale, with higher scores indicating better capability.

For peer review only - http://bmjopen.bmj.com/site/about/guidelines.xhtml

- Anxiety and depression. Measured using the Hospital Anxiety and Depression Scale (HADS[26]), a 12-item questionnaire in which higher scores on depression and anxiety subscales indicate higher anxiety across two subscales (anxiety and depression).
- Enablement, measured using a modified version of the patient enablement instrument (PEI[27]) that has been validated in previous RCTs[28]. The modified PEI is a 7-point scale consisting of 6 items, in which higher scores indicate more enablement.
- Patient satisfaction was measured by asking patients whether they saw any benefits or disadvantages to using MBM, and whether they would recommend it to friends and family based on the NHS Friends and Family Test (FFT[29]).
- Patient burden was measured using a specifically developed questionnaire exploring time and costs via self-report based on Burden of Treatment Theory[30]. The questionnaire consisted of 4 questions with descriptive responses that explored whether new programmes were signed up to (such as gym membership, yoga/meditation), and the financial burden of doing so (see Appendix 1).

Physiological measures of lung function were taken at baseline and 12 month appointments: forced expiratory volume (FEV_1), ratio of forced expiratory volume to forced vital capacity (FEV_1/FVC) and peak expiratory flow rate (PEFR).

At 12-month follow-up, we also monitored health resource use GP consultations, A&E visits, hospital admissions, asthma medication use and use of antibiotics for chest infections using GP practice patient notes.

Healthcare utilisation data were collected via retrospective notes review conducted by practice staff. Staff were provided with a template for reviewing data, and an instruction manual to ensure correct data were provided. Initial notes reviews were completed within two months from completion of primary data collection. Nine patients (10% of the total patients in each practice) were also reviewed by research nurses to assess data quality.

Patient and Public Involvement

For peer review only - http://bmjopen.bmj.com/site/about/guidelines.xhtml

For peer review only

BMJ Open

Asthma UK was involved in the initial project proposal and supported the project throughout. Patient and public representatives (recruited with help from Asthma UK) participated in intervention development (providing feedback on prototype versions of the intervention, attending study management meetings, helping to develop trial materials and procedures, and discussing responses to participant feedback). Asthma UK are involved in dissemination of this research and ongoing projects related to the research.

Data Analysis

Primary analysis of the study was a description of key feasibility outcomes including patient eligibility, recruitment rates, withdrawals, 3 and 12-month follow-up response rates and digital intervention usage, as reported in the trial protocol (see Supplementary File 1).

Descriptive statistics were used to identify any floor or ceiling effects. For continuous measures, means, standard deviations (SD) and 95% CIs were reported at baseline, 3-month and 12-month for each group, as well as for the sample as whole.

Exploratory analysis explored group differences in continuous primary endpoint measures (AQLQ, ACQ, HADS, PEI) using linear regression models that controlled for baseline values. Participants were analysed in the group to which they had been randomised and comprised complete cases only.

Proportions of patients achieving a minimal clinically important difference (MCID) was described for asthma quality of life (the AQLQ MCID is 0.5[16]).

Sensitivity analysis explored missing data at 3 and 12 months.

Healthcare utilisation outcomes were explored using a negative binomial model of group count data.

Health economic analysis was descriptive, reporting estimates of cost and outcomes measures and baseline and follow-up. The completeness and suitability of EQ-5D-5L and ICECAP were

For peer review only - http://bmjopen.bmj.com/site/about/guidelines.xhtml

BMJ Open

compared as was the appropriateness of the resource use, and time and cost tools developed for the study.

Intervention engagement was descriptive.

Results

Recruitment and retention

Six practices were initially recruited and after monitoring recruitment rates a seventh practice added. In this additional practice only half the list (randomly selected) were offered participation in the study to avoid over recruiting. Across the 7 practices, 68478 patients were assessed for eligibility with 3199 meeting initial eligibility criteria (asthma diagnosis, >1 asthma medicine prescription in last 12 months, screened by practice). 266 patients completed postal screening measures before the recruitment period finished, of whom 125 were eligible to take part (impaired asthma-related quality of life, AQLQ score less than 5.5). Ninety patients responded to further contact. Two patients did not attend their baseline appointment leaving a final sample of 88 patients (intervention N = 44, usual care N = 44) who were recruited into the study (13.5 per practice) and were randomised over a 5 month period. Figure 1 presents the study CONSORT diagram.

During the study, 2 patients withdrew before 3-month follow-up and 2 before 12-month follow-up. All were in the intervention group. Patients withdrew for several reasons including lack of time (N = 1), illness (N = 1), death of family member (N = 1), and lack of perceived benefit (N = 1). 3 of 4 participants who withdrew had used the intervention; one had not. One participant was withdrawn from the study prior to 12-month follow-up they were no longer eligible (i.e. they were referred to secondary care).

Follow-up rates at 3 months were 91% (80/88; intervention: 36/44, control 44/44). Six (7%) patients did not complete 3 month follow-up measures but did not withdraw (all in intervention group).

For peer review only - http://bmjopen.bmj.com/site/about/guidelines.xhtml

BMJ Open

At 12 months, 91% of participants provided primary outcome data by attending a follow-up appointment or returning a postal questionnaire (80/88; intervention: 37/45; control 43/43). 76% attended a baseline appointment and provided secondary clinical data (67/88). Four (5%) patients did not complete 12 month follow-up measures but did not withdraw (3 in intervention group; 1 in usual care). None of these intervention participants had used the intervention. None of these patients responded to efforts to contact them by the study team.

For peer review only

For peer review only - http://bmjopen.bmj.com/site/about/guidelines.xhtml

BMJ Open

Patient characteristics

Demographics and baseline characteristics of participants are presented in Table 1 and were reasonably well-balanced between arms across all measures.

Table 1: Baseline demographic characteristics of study population per group.

M (SD)		Overall sample (N = 88)	Intervention group (N = 44)	Control Group (N = 44)	Lost to follow-up (N = 8)
**Age**		56.6 (15.2)	57.0 (14.2)	56.3 (16.2)	53.5 (12.11)
**Female N (%)**		53.0 (60.2)	27.0 (61.4)	26.0 (59.1)	6 (75)
**BMI**		29.5 (6.1)	28.9 (5.9)	30.1 (6.3)	32.7 (4.3)
**Length of diagnosis**		24.0 (17.5)	25.2 (17.2)	22.8 (17.8)	30 (18.9)
**FEV_1**		2.5 (0.8)	2.6 (0.8)	2.5 (0.8)	2.40 (0.47)
**% Predicted FEV_1**		92.3 (16.0)	94.8 (16.0)	89.8 (15.8)	92.0 (12.9)
**FEV_1 / FVC**		76.6 (8.5)	77.1 (8.0)	76.1 (9.0)	74.9 (4.1)
**Peak Flow**		421.2 (104.7)	421.3 (108.3)	421.1 (102.3)	420.6 (83.8)
**Ethnicity**	**White N (%)**	84 (95.5)	42 (95.5)	42 (95.5)	7 (87.5)
	**Other N (%)**	4 (4.5)	2 (4.5)	2 (4.5)	1 (12.5)
**Smoking Status**	**Current N (%)**	9 (10.2)	7 (15.9)	2 (4.5)	2 (25.0)
	**Former N (%)**	29 (33.0)	13 (29.5)	16 (36.3)	3 (37.5)
	**Never N (%)**	50 (56.8)	24 (54.5)	26 (59.1)	3 (37.5)
**Age left education**		18.5 (5.3)	19.4 (7.0)*	17.7 (2.7)	20.4 (8.2)
	**16 or under N (%)**	40 (46.5)	18 (42.9)	22 (50.0)	4 (50.0)
	**17-18 N (%)**	22 (25.6)	9 (21.4)	13 (29.5)	1 (12.5)
	**Above 18 (%)**	24 (27.9)	15 (35.7)	9 (20.5)	3 (37.5)
**Index of Multiple Deprivation Mean Rank (Median Decile)**		17192 (5.5)	17231 (6.5)	17212 (5)	4505.5 (1.5)
**AQLQ**		4.81 (1.01)	4.85 (0.94)	4.78 (1.09)	4.26 (0.55)
**ACQ**		1.45 (0.80)	1.35 (0.66)	1.56 (0.91)	1.52 (0.73)
**HADS-A**		6.60 (4.47)	6.57 (3.87)	6.64 (5.04)	8.63 (3.9)
**HADS-D**		3.89 (3.57)	3.39 (3.07)	4.39 (3.99)	4.75 (4.4)
**EQ5D-5L**		0.83 (0.19)	0.86 (0.15)	0.81 (0.22)	4 (50.0)
**EQ5D-VAS**		71.5 (18.2)	70.0 (19.3)	73.0 (17.2)	1 (12.5)
**ICECAP-A**		0.87 (0.18)	0.89 (0.12)	0.88 (0.16)	3 (37.5)
**PEI**		2.52 (1.23)	2.44 (1.09)	2.60 (1.37)	2.73 (1.0)
**MARS-A**		4.70 (1.05)	4.80 (0.90)	4.60 (1.20)	4.3 (0.8)

Note: (*) Percentages are reported from 42 participants as two participants in the intervention group did not complete this data.

For peer review only - http://bmjopen.bmj.com/site/about/guidelines.xhtml

BMJ Open

Table 1 compares those lost to follow-up to those who remained in the study at 3 months in a sensitivity analysis. Those lost to follow up were slightly more likely to be female, have a higher BMI, a longer time since diagnosis, a lower AQLQ score, a higher HADS-A and HADS-D score and to be from a more deprived postcode.

Intervention usage and engagement

At 12 month follow-up, 36 (82%) patients in the intervention arm had engaged with the intervention (at least 1 log in). Patients logged in between 0 and 25 times to the intervention (Median=4; IQR=8.25). Several patients also engaged with additional lifestyle modification interventions including improving hand hygiene (N = 2), weight loss (N = 3), improving physical activity (N = 3) and getting support from friends and family (N = 5).

After the study, participants in the intervention group were asked 'Do you think there were any benefits to using My Breathing Matters?'. 12 of 36 (33%) reported 'quite a bit/a large amount of benefit', 19/36 (53%) reported 'some benefit', and 5/36 (14%) reported 'very little benefit'. 22 participants completed a free text box describing the advantages - benefits varied but included information provision (such as 'weight loss', 'dietary/exercise regimes'), medication adherence (such as asthma action plans, improved medication adherence), provision of non-pharmacological treatments (such as breathing exercises and relaxation) and accessibility (such as 'access to information quickly'). This is reported in more detail in a separately published process analysis.

Participants were also asked 'Do you think there were any disadvantages to using My Breathing Matters?'. Twenty five of 36 (69%) reported no disadvantages at all, 3 (8%) reported very few disadvantages, 8 (22%) reported some disadvantages, and 0 reported quite a bit or a large amount of disadvantages. 13 participants completed a free text box describing disadvantages, which included technical difficulties (such as not always accessible across different devices, difficulty logging in) and information specificity (such as not enough information, too many

For peer review only - http://bmjopen.bmj.com/site/about/guidelines.xhtml

reminders, too few reminders). A final question asked how likely participants were to recommend MBM to friends or family. Sixteen participants (44%) were extremely likely to recommend it, 12 participants (33%) were likely, 7 (19%) were neither likely nor unlikely, and 1 was extremely unlikely (3%).

Trial Endpoint Measures

The full data of the trial endpoints is set out in Table 2.

Both the intervention group and control group improved from baseline to 3-month and 12-month follow-up, with numerically larger improvements in the asthma-related patient reported outcomes measuring quality of life and symptom control (AQLQ and AQC) at both time points; one or both these measure are anticipated to be the primary outcome of a subsequent fully powered study.

At the 3-month evaluation, patients in the intervention group who completed 3 month follow-up measures (N = 36) had mean improvement in asthma-related quality of life (AQLQ score) of 0.53 (95% CI: 0.31, 0.75), and in the control group of 0.52 (95%CI: 0.30, 0.74), with the between-group difference (controlling for baseline differences) the AQLQ being 0.06 higher (95% CI -0.22, 0.35) in the intervention group, indicating better quality of life. By 12 months, these figures were 0.35 (0.10, 0.60) and 0.21 (-0.09, 0.51) respectively, and the between-group difference had risen to 0.18 (95% CI -0.21, 0.56) higher in the intervention group. In the ACQ analysis, at the 3-month analysis, the between-groups ACQ score was 0.14 lower (95% CI -0.41, 0.13) in the intervention group, indicating better control, and at 12 months was 0.14 lower (95% CI -0.40, 0.11). These findings indicate consistent trends to improvement in both asthma quality of life and asthma control in the intervention group compared to the control. Full follow-up data are presented in Table 2.

For peer review only - http://bmjopen.bmj.com/site/about/guidelines.xhtml

BMJ Open

Table 2: 3 and 12-month follow up data (corrected for baseline differences)

Measure	Intervention group (N = 36)			Control Group (N = 44)			Difference between the intervention and control group controlling for baseline (95% CI)
	M (SD)	% >MCID* Improvement	% items complete	M (SD)	% >MCID* Improvement	% items complete	
**3-month**							
AQLQ	5.51 (0.85)	47.2	82	5.30 (1.07)	47.7	100	0.06 (-0.22, 0.35)
ACQ	0.98 (0.65)		82	1.28 (0.87)		100	-0.14 (-0.41, 0.13)
HADS-A	6.75 (3.85)		82	7.07 (5.48)		100	-0.04 (-0.18, 0.11)
HADS-D	3.75 (2.82)		82	4.66 (4.99)		100	-0.02 (-0.16, 0.13)
PEI	2.71 (1.09)		82	2.90 (1.14)		100	-0.12 (-0.59, 0.35)
MARS-A	4.23 (0.70)		80	4.05 (0.74)		100	0.04 (-0.25, 0.3-)
EQ-5D-5L	0.82 (0.19)		82	0.83 (0.20)		100	-
ICECAP-A	0.87 (0.12)		82	0.84 (0.19)		100	-
**12-month**							
AQLQ	5.29 (0.98)	38.9	82	5.00 (1.25)	39.5	98	0.18 (-0.21, 0.56)
ACQ	1.00 (0.59)		82	1.26 (0.69)		98	-0.14 (-0.40, 0.11)
HADS-A	7.78 (3.94)		84	6.63 (4.91)		98	0.99 (0.16, 2.15)
HADS-D	3.81 (3.54)		84	4.19 (4.17)		98	0.22 (-0.97, 1.41)
PEI	2.46 (1.03)		84	2.61 (1.28)		98	-0.09 (-0.54, 0.37)
MARS-A	4.37 (0.81)		82	4.29 (0.85)		98	-0.09 (-0.43, 0.25)
EQ-5D-5L	0.83 (0.21)		82	0.80 (0.23)		98	-
ICECAP-A	0.86 (0.13)		82	0.84 (0.20)		98	-
FEV_1 (litres)	2.75 (0.75)		57	2.43 (0.74)		80	0.03 (-0.05, 0.10)
FEV_1 / FVC	78.8 (6.58)		57	76.3 (9.29)		80	2.20 (-0.13, 4.27)
% Predicted FEV_1	100.1 (14.8)		57	92.4 (13.8)		80	1.77 (-1.72, 5.25)
Peak Flow	450 (105)		57	417 (102)		80	15.29 (-6.27, 36.86,)
BMI	28.7 (6.17)		64	31.1 (6.51)		86	-0.11 (-0.89, 0.68)

There was no difference in number of patients who showed MCID improvement at 3 months (AQLQ, >0.5) across groups (47.2% in the intervention group compared to 47.7% in the control group). The same was true at 12 months (38.9% compared to 39.5%).

For peer review only - http://bmjopen.bmj.com/site/about/guidelines.xhtml

For peer review only

BMJ Open

Adverse Events

Adverse events were reported by GPs and nurses who contacted the study team to report both adverse and serious adverse events. Nine adverse events were reported (intervention N = 6, usual care N = 3). These were assessed by research team clinicians and all were considered unlikely to be related to the study. Three were related to participant asthma (asthma exacerbation not leading to hospital admission, upper respiratory tract infection, sinusitis).

Three serious adverse events were reported (intervention N = 2, usual care N = 1). These were considered unlikely to be related to the study and the condition (atrial fibrillation, open distal radius fracture, cardioversion).

For peer review only

Healthcare Utilisation Outcomes

Data were collected from retrospective notes reviews (conducted by practice nurses) from 83 participants, reported in Table 3. Data was collected from 84 practices for 7 participants, with 4 participants from 1 practice incomplete. The data quality check, and subsequent examination by research team clinicians (MT) found that reviews completed by the practice nurses varied substantially in quality with varied levels of detail, and the quality of data achieved in this way was insufficient for a health economic analysis.

Table 3: Data on asthma related medication use (during the study period)

Healthcare utilisation (N, IQR)<br>Mean (SD)	Intervention group		Control group	
	12m before study period	12m after study period	12m before study period	12m after study period
SABA Prescriptions	3 (2,6)<br>3.92 (3.48)	3 (1,6)<br>4.00 (3.72)	3 (2,5)<br>4.0 (3.89)	4 (2,6)<br>4.39 (3.81)
ICS Prescriptions	5 (2,11)<br>6.72 (4.92)	4 (3,10)<br>6.15 (4.21)	6 (4,10)<br>7.41 (5.45)	6 (4,10)<br>7.34 (5.37)
Oral steroids prescriptions	0 (0,0)<br>0.31 (0.80)	0 (0,0)<br>0.36 (0.94)	0 (0,0)<br>0.43 (1.07)	0 (0,0)<br>0.23 (0.71)
Antibiotic Prescriptions	0 (0,0)<br>0.33 (0.87)	0 (0,0)<br>0.28 (0.60)	0 (0,0)<br>0.52 (1.45)	0 (0,1)<br>0.16 (0.48)

Note: (*) Incidence rate ratio vs. intervention group, controlling for 12 months before study period.

For peer review only - http://bmjopen.bmj.com/site/about/guidelines.xhtml

Comparisons between group count data were reported using a negative binomial model but given the issues with the reliability of the data, should be interpreted cautiously. The prescription rate was approximately 8% higher in for both SABA (IRR 1.08, 95% CI 0.82, 1.43) and ICS (IRR 1.08, 95% CI 0.86, 1.35) in the control group compared to the intervention group. Both groups had a low number of prescriptions for oral steroids, oral steroids and antibiotics, with only 15 prescriptions in total for either of these medications, making between group comparisons unreliable.

Due to unreliability of data, frequency of GP consultations, A&E admissions and hospitalisations have not been reported.

Health Economic Outcomes

Both EQ-5D-5L and ICECAP-A had the same completion rates as other secondary measures completed at follow up (see Table 2).

Patients reported several programmes across both groups including gym, walking, yoga, sewing, language courses, physio and signing (see Appendix 2). There were no substantial differences in terms of numbers or costs although the sample size was small.

Discussion

In line with our main research objectives, findings from our randomised controlled feasibility trial demonstrate that a full-size confirmatory trial to confirm effectiveness of MBM, a digital self-management intervention for adults in primary care with asthma is likely to be feasible and acceptable. Our trial procedures, intervention usage and data management were all feasible. There were also trends to improved asthma control and quality of life in our underpowered sample, so supporting the need for a definitive fully-powered study. Our recruitment

For peer review only - http://bmjopen.bmj.com/site/about/guidelines.xhtml

BMJ Open

procedures recruited a specific patient sample (those impaired asthma-specific quality of life) to target from a range of urban and rural practices.

Our sample varied in age with a relatively high mean (56 years) indicating that our digital intervention can provide benefit to older adults. Both male and female adults were well-represented in our trial. A notable proportion of our sample was obese (41%), in line with previous findings[11]. Given that obesity is a risk factor for asthma, a larger trial could further improve effectiveness by providing more specific behavioural content for obese adults with asthma (such as tailored content to increase motivation to use weight-loss related lifestyle components in obese patients). Our sample was also predominantly white. Underrepresentation of minority ethnic groups in medical research in the UK is an ongoing issue[12] and should be addressed in recruitment procedures in the full trial.

The feasibility of a full trial is supported by the effective completion of trial procedures. All patients who completed baseline measures were randomised. Completion of measures was good at both follow-up points (3-month via post and 12-month at participants' practice). Where participants were not able to attend a follow-up appointment at practices, they were satisfactorily followed up via post or telephone for main trial measures. 8 patients were lost to follow-up (4 withdrew and 4 no longer responded to attempts to contact them). Notably, all 8 patients lost-to-follow were in the intervention group. It is possible that patients in the control group were more likely to maintain contact as they were only able to access the intervention upon completion of 12-month follow up measures. Although loss to follow-up is low, it is important to consider whether that loss is differential. Those lost to follow-up were more likely to be more socio-economically deprived, female, have a higher BMI, a longer time since diagnosis and a higher HADS-A and HADS-D score. It is possible that these patients would benefit from using MBM more than most, and therefore we have proposed several ways to further increase trial efficacy. Automatic email intervention registration at baseline (patients cannot attend baseline appointment without enrolling on the intervention) would increase

For peer review only - http://bmjopen.bmj.com/site/about/guidelines.xhtml

BMJ Open

initial engagement and engagement with trial procedures throughout duration of study. Online questionnaire completion during screening process would i) screen patients who are unable to interact with online trial/intervention and therefore unable to benefit from the intervention (feasibility trial estimate = 2%), and ii) streamline baseline/follow-up procedures.

Both health economic outcomes had high completion rates but did not suggest substantive change, similar to EQ-5D measures in previous non-pharmacological self-management trials (such as [8]). It is possible that an alternative measure such as the Short Form 12-item Survey (SF-12[31]) in which participants consider the previous two weeks (whereas in the EQ-5D they consider the immediate present) may be better suited to measure small yet valuable changes in well-being over a full trial. Our detailed mixed-methods process analysis explored issues of trial acceptability in more detail, and will be reported in a subsequent paper. A full trial of this non-pharmacological intervention should accurately capture 'non-medical' costs (such as gym membership) that are likely to impact disease-specific quality of life, as well as medical costs that would be affected by changes in healthcare utilisation.

Healthcare utilisation data were collected by practice nurses whose main role was to provide usual clinical care at the practices, using a manual to guide data collection, rather than by trained research nurses, and our quality check demonstrated that the data collection process used was unreliable some centres. We conclude that in a full subsequent study, these data should be collected from the medical record by a trained member of the study team (such as a trained research nurse), as has been successfully used in previous studies[8].

Engagement with the intervention was slightly increased compared to a previous similar digital asthma self-management intervention[19] at initial sign up (82% vs 76%) as well as maintaining a higher number of ongoing engagement throughout the follow up period (median 3 additional log ins vs. 1), although our study used a broad primary care population while the RAISIN protocol primarily recruited from areas of high deprivation. This finding demonstrates that the use of the person-based approach to develop the intervention resulted in an

For peer review only - http://bmjopen.bmj.com/site/about/guidelines.xhtml

BMJ Open

intervention that was acceptable and engaging to patients, even using a pragmatic methodology in which patients self-registered at home instead of being registered by a GP during their baseline appointment. Participants accessed both pharmacological and non-pharmacological self-management content. We further explored the acceptability of the intervention to people with asthma in a mixed-methods process analysis which will be published separately.

Estimates of effect size demonstrated that participants who received the intervention and completed follow-up measures showed improved and clinically relevant quality of life and asthma control. The order of magnitude of the mean between-group improvements in the patient reported measures of control (ACQ) and asthma-related QOL (AQLQ), although not statistically significant with the sample size of this feasibility study, was comparable to that reported in controlled studies of pharmacological[32] and non-pharmacological[8] interventions in asthma, and so justify a confirmatory study with a fully-powered sample.

There was no suggestion of an effect on physiological measures of lung function. These results are in line with previous studies of behavioural self-management interventions in primary care adults with asthma (such as BREATHE, RAISIN), and demonstrate the importance of interventions targeting outcomes that incorporate elements of functional wellbeing (disease specific quality of life, subjective symptoms), rather than solely focusing on objective, physiological measures that are not correlated with quality of life.

The effectiveness of our intervention could be further increased according to findings from our process evaluation. This analysis, which will be reported separately, broadly agrees with previous research[19] in finding that many patients consider their asthma to be 'well-controlled' despite having important levels of symptoms and quality of life impairment on validated questionnaire. This implies that many people had become accustomed to their on-going symptoms and had altered their life to try to reduce their impact, using denial as a coping mechanism. As a consequence, the means of appropriately targeting and framing self-management interventions should be carefully considered in future work, focussing on

For peer review only - http://bmjopen.bmj.com/site/about/guidelines.xhtml

maintaining good health rather than improving poor health. Some of our findings (such as the association between quality of life improvement and ongoing intervention engagement) demonstrate that framing content as positive and not focusing on illness – for example 'How to keep your breathing healthy' rather than 'How to reduce asthma symptoms' may lead to an acceptable, engaging intervention that benefits this patient group.

There were some limitations to this small feasibility study. Although our researchers and statisticians were blind to group allocation, patients would have known that they were allocated to the intervention rather than the usual care control. This is common in complex behavioural interventions. Furthermore, although we endeavoured to recruit participants across a broad demographic range, the reach of our intervention could be improved. While the reach of digital interventions improves as digital literacy increases nationally, care must be taken to ensure that 'digital transformation' of NHS services does not entrench healthcare inequality, by facilitating a 'digital divide' that fails to provide adequate health and social care to those who do not have the digital skills to benefit.

Conclusion

Our findings demonstrate the feasibility of a new digital self-management intervention for asthma (MBM). Using the person-based approach to intervention development means that MBM is both acceptable and engaging for adults with asthma in primary care. MBM reflects the varied experiences of people with asthma, by including both non-pharmacological and pharmacological components. Our data support the feasibility of moving towards a fully-powered RCT, with only minor modifications to some trial procedures required.

For peer review only - http://bmjopen.bmj.com/site/about/guidelines.xhtml

BMJ Open

Acknowledgements: The authors would like to acknowledge the contributions of all participants and patient and public representatives in the development of the My Breathing Matters intervention development and feasibility evaluation, and Dr Deborah Morrison who lead the development of 'Living Well With Asthma' resource that guided development of My Breathing Matters.

Protocol: The trial protocol has been along with this publication as a supplementary file (Supplementary File 1).

Ethics: The study was approved by NHS South Central – Berkshire Research Ethics Committee, 16/01/2017, ref: 16/SC/0614.

Competing Interests: Neither MT nor any member of his close family has any shares in pharmaceutical companies. In the last 3 years he has received speaker's honoraria for speaking at sponsored meetings or satellite symposia at conferences from the following companies marketing respiratory and allergy products: GSK, Novartis. He has received honoraria for attending advisory panels with; Boehringer Inglehiem, GSK, Novartis. He is a recent a member of the BTS SIGN Asthma guideline steering group and the NICE Asthma Diagnosis and Monitoring guideline development group. BA, KG, JR, BS, LY, FM and AB have no competing interests.

Author Contributions: LY, MT and AB conceived the idea for the study. LY, MT, JR, FM, BS & AB secured funding for the study. BA, LY, AB, BS MT developed the intervention and designed the trial with input from Asthma UK and FM. BA and KG managed the trial on a day-to-day basis with support from LY and MT. BS and JR planned and carried out the statistical analysis. BA drafted the manuscript with assistance and final approval from all authors. BA is the guardian of the data.

Funding Statement: This article presents independent research funded by the National Institute for Health Research (NIHR) under its Programme Grants for Applied Research (PGfAR) Programme (Grant Reference Number RP-PG-1211-20001). LY is an NIHR Senior Investigator and during the study BA was supported by an NIHR School of Primary Care (SPCR) Fellowship.

For peer review only - http://bmjopen.bmj.com/site/about/guidelines.xhtml

BMJ Open

Prof Anne Bruton was funded by the National Institute for Health Research (NIHR) Senior Research Fellowship (SRF-2012-05-120). The views expressed are those of the authors and not necessarily those of the NIHR or the Department of Health and Social Care.

Data Sharing: Deidentified participant data is available from the corresponding author (BA) upon reasonable request. Please contact via email (b.ainsworth@bath.ac.uk). Data availability will be granted providing it is in line with consent granted by participants, for as long as possible.

Word Count: 5366.

For peer review only

For peer review only - http://bmjopen.bmj.com/site/about/guidelines.xhtml

BMJ Open

References

1. Global Asthma Network. The Global Asthma Report 2014. Vol. 5, Auckland, New Zealand. 2014. 14–16

2. Demoly P, Gueron B, Annunziata K, Adamek L, Walters RD. Update on asthma control in five European countries: Results of a 2008 survey. Eur Respir Rev. 2010;19(116):150–7. doi: 10.1183/09059180.00002110.

3. Guidelines for management of asthma in adults: I--Chronic persistent asthma. Statement by the British Thoracic Society, Research Unit of the Royal College of Physicians of London, King's Fund Centre, National Asthma Campaign. BMJ [Internet]. 1990 Sep 29 [cited 2019 Jun 17];301(6753):651–3. Doi: 10.1136/bmj.301.6753.651

4. Ainley A, Gildeh N, Seeley A, Lewis K, Maisel T, Jayasinghe R, et al. Does having specialist asthma nurses improve adherence to national asthma guidelines? A comparison between two UK district general hospitals. In: 111 Clinical Problems - Asthma [Internet]. European Respiratory Society; 2015 [cited 2019 Jun 17]. p. PA644. Doi: 10.1183/13993003.congress-2015.PA644.

5. Gibson PG, Powell H, Coughlan J Wilson AJ, Abramson M, Haywood P, Bauman A, Hensley MJ, Walters EH. Self-management education and regular practitioner review for adults with asthma. Cochrane Database Syst Rev. 2002;1:CD001117. doi: 10.1002/14651858.CD001117.

6. Pinnock H, Parke HL, Panagioti M, Daines L, Pearce G, Epiphaniou E, et al. Systematic meta-review of supported self-management for asthma: a healthcare perspective [Internet]. Vol. 15, BMC Medicine. BMC Medicine; 2017. doi: 10.1186/s12916-017-0823-7

7. Asthma UK. Annual Asthma Survey 2018 [Internet]. 2018 [cited 2019 Jun 17].

8. Bruton A, Lee A, Yardley L, Raftery J, Arden-Close E, Kirby S, et al. Physiotherapy breathing retraining for asthma: a randomised controlled trial. Lancet Respir Med [Internet]. 2017;2600(17):1–10. doi: 10.1016/S2213-2600(17)30474-5.

9. Department of Health and Social Care. The future of healthcare: our vision for digital, data and technology in health and care [Internet]. UK Government Policy Paper. 2018 Oct [cited 2019 Jun 17].

10. van Gaalen JL, Beerthuizen T, van der Meer V, van Reisen P, Redelijkheid GW, Snoeck-Stroband JB, et al. Long-term outcomes of internet-based self-management support in adults with asthma: randomized controlled trial. J Med Internet Res [Internet]. 2013 Sep 12 [cited 2019 Jun 17];15(9):e188. doi: 10.2196/jmir.2640

11. Morrison D, Wyke S, Saunderson K, McConnachie A, Agur K, Chaudhuri R, et al. Findings from a pilot Randomised trial of an Asthma Internet Self-management Intervention (RAISIN). BMJ Open [Internet]. 2016 May 12 [cited 2019 May 28];6(5):e009254. doi: 10.1136/bmjopen-2015-009254.

12. McLean G, Band R, Saunderson K, Hanlon P, Murray E, Little P, et al. Digital interventions to promote self-management in adults with hypertension systematic review and meta-analysis. BMC Pulm Med [Internet]. 2016;34(4):600–12. doi: 10.1097/HJH.0000000000000859.

13. Band R, Bradbury K, Morton K, May C, Michie S, Mair FS, et al. Intervention planning for a digital intervention for self-management of hypertension: a theory-, evidence- and person-based approach. Implement Sci [Internet]. 2017;12(1):25. doi: 10.1186/s13012-017-0553-4.

For peer review only - http://bmjopen.bmj.com/site/about/guidelines.xhtml

14. Moore GF, Audrey S, Barker M, Bond L, Bonell C, Hardeman W, et al. Process evaluation of complex interventions: Medical Research Council guidance. BMJ [Internet]. 2015 Mar 19 [cited 2019 Jun 17];350:h1258. Doi: 10.1136/BMJ.H1258.

15. Smith T, Noble M, Noble S, Wright G, McLennan D, Plunkett E. The English indices of deprivation 2015. London: Department for Communities and Local Government. 2015 Sep.

16. Juniper EF, Guyatt GH, Cox FM, Ferrie PJ, King DR. Development and validation of the Mini Asthma Quality of Life Questionnaire. Eur Respir J [Internet]. 1999 Jul 1 [cited 2019 Jun 17];14(1):32–8. Doi: 10.1034/j.1399-3003.1999.14a08.x

17. Yardley L, Osmond A, Hare J, Wills G, Weal M, De Roure D, et al. Introduction to the LifeGuide: software facilitating the development of interactive behaviour change internet interventions. In: Proceedings of the WebSci'09: Society On-Line. Athens, Greece [Internet]. 2009 [cited 2019 Jun 17].

18. Hoffmann TC, Glasziou PP, Boutron I, Milne R, Perera R, Moher D, et al. Better reporting of interventions: template for intervention description and replication (TIDieR) checklist and guide. Bmj [Internet]. 2014;348(mar07 3):g1687–g1687. doi: 10.1136/bmj.g1687.

19. Morrison D, Mair FS, Chaudhuri R, McGee-Lennon M, Thomas M, Thomson NC, et al. Details of development of the resource for adults with asthma in the RAISIN (randomized trial of an asthma internet self-management intervention) study. BMC Med Inform Decis Mak [Internet]. 2015;15(1):57. doi: 10.1186/s12911-015-0177-z.

20. Yardley L, Morrison L, Bradbury K, Muller I. The Person-Based Approach to Intervention Development : Application to Digital Health-Related Behavior Change Interventions Corresponding Author : J Med Internet Res. 2015;17(1):e30. doi: 10.2196/jmir.4055.

21. Yardley L, Ainsworth B, Arden-Close E, Muller I. The person-based approach to enhancing the acceptability and feasibility of interventions. Pilot Feasibility Stud [Internet]. 2015 Dec 26 [cited 2019 Apr 26];1(1):37. doi: 10.1186/s40814-015-0033-z.

22. Asthma UK. Live Well With Asthma Booklet [Internet]. 2016. Available from: https://www.asthma.org.uk/197182a8/globalassets/health-advice/resources/adults/live-well-with-asthma-booklet.pdf

23. Juniper EF, O'Byrne PM, Guyatt GH, Ferrie PJ, King DR. Development and validation of a questionnaire to measure asthma control. Eur Respir J [Internet]. 1999;14:902–7. Doi: 10.1034/j.1399-3003.1999.14d29.x.

24. EuroQol - a new facility for the measurement of health-related quality of life. Health Policy (New York) [Internet]. 1990 Dec 1 [cited 2019 Mar 8];16(3):199–208. doi: 10.1016/0168-8510(90)90421-9.

25. Al-Janabi H, N Flynn T, Coast J. Development of a self-report measure of capability wellbeing for adults: the ICECAP-A. Qual Life Res [Internet]. 2012 Feb 20 [cited 2019 Mar 8];21(1):167–76. doi: 10.1007/s11136-011-9927-2.

26. Zigmond AS, Snaith RP. The Hospital Anxiety And Depression Scale. Acta Psychiatr Scand [Internet]. 1983;67(6):361–70. doi: 10.1186/1477-7525-1-29.

27. Howie JG, Heaney DJ, Maxwell M, Walker JJ. A comparison of a Patient Enablement Instrument (PEI) against two established satisfaction scales as an outcome measure of primary care consultations. Fam Pract [Internet]. 1998 Apr 1 [cited 2019 Jun 17];15(2):165–71. doi: 10.1093/fampra/15.2.165.

28. Little P, Lewith G, Webley F, Evans M, Beattie A, Middleton K, et al. Randomised controlled

For peer review only - http://bmjopen.bmj.com/site/about/guidelines.xhtml

trial of Alexander technique lessons, exercise, and massage (ATEAM) for chronic and recurrent back pain. BMJ [Internet]. 2008 Aug 19 [cited 2019 Jun 17];337:a884. doi: 10.1136/BMJ.A884.

29. Sizmur S, Graham C, Walsh J. Influence of patients' age and sex and the mode of administration on results from the NHS Friends and Family Test of patient experience. J Health Serv Res Policy [Internet]. 2015 Jan 27 [cited 2019 Jun 17];20(1):5–10. doi: 10.1177/1355819614536887

30. May CR, Eton DT, Boehmer K, Gallacher K, Hunt K, MacDonald S, et al. Rethinking the patient: using Burden of Treatment Theory to understand the changing dynamics of illness. BMC Health Serv Res [Internet]. 2014 Dec 26 [cited 2019 Jun 17];14(1):281. doi: 10.1186/1472-6963-14-281.

31. Ware J, Kosinski M, Keller SD. A 12-Item Short-Form Health Survey: construction of scales and preliminary tests of reliability and validity. Med Care [Internet]. 1996 Mar [cited 2019 Jun 17];34(3):220–33. doi: 10.2307/3766749

32. Bateman ED, Esser D, Chirila C, Fernandez M, Fowler A, Moroni-Zentgraf P, et al. Magnitude of effect of asthma treatments on Asthma Quality of Life Questionnaire and Asthma Control Questionnaire scores: Systematic review and network meta-analysis. J Allergy Clin Immunol [Internet]. 2015 Oct 1 [cited 2019 Jun 17];136(4):914–22. doi: 10.1016/J.JACI.2015.03.023.

For peer review only

For peer review only - http://bmjopen.bmj.com/site/about/guidelines.xhtml

BMJ Open

Figure Captions

Figure 1. Study Consort Diagram

For peer review only

For peer review only - http://bmjopen.bmj.com/site/about/guidelines.xhtml

Figure 1. Study Consort Diagram

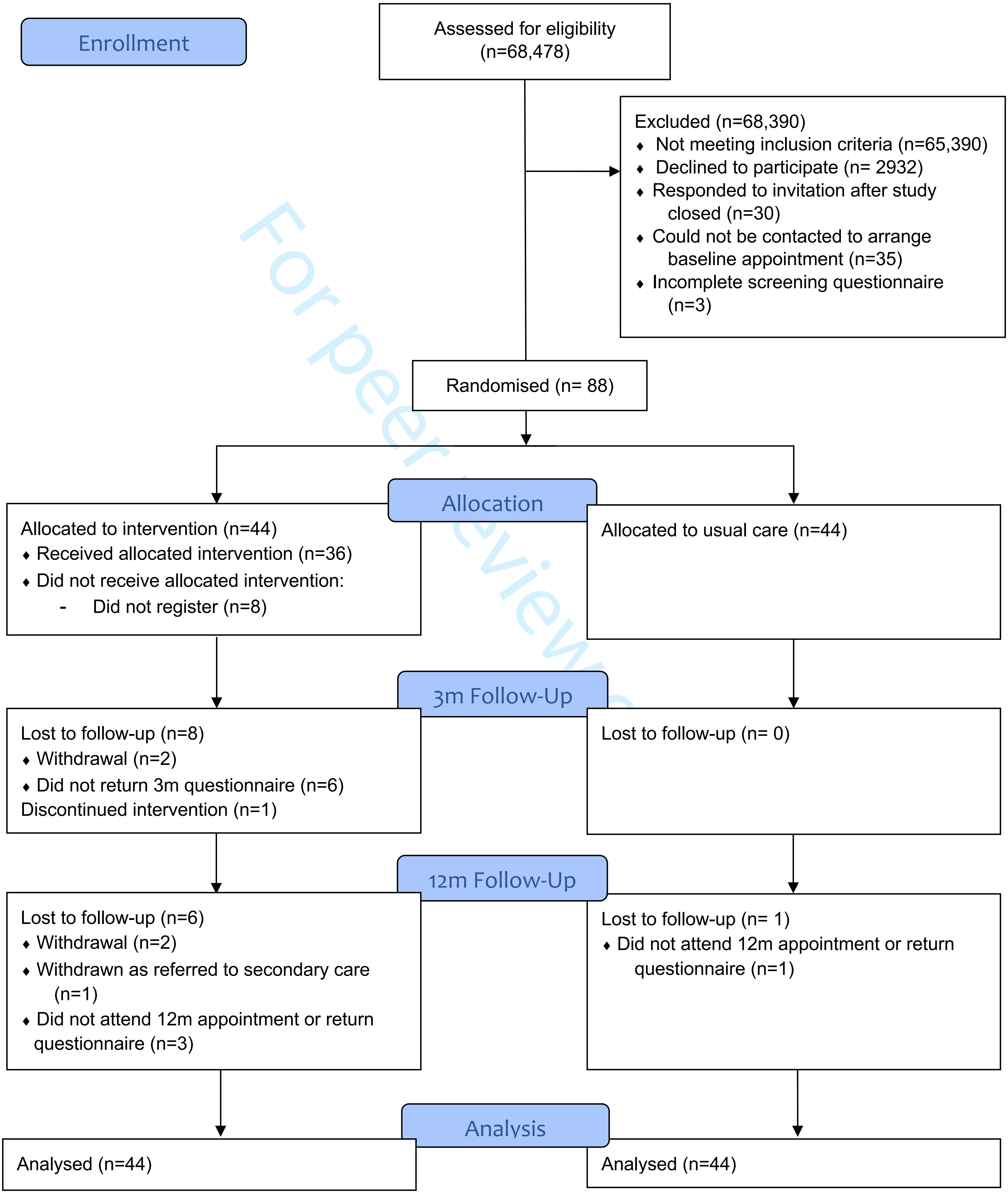

For peer review only - http://bmjopen.bmj.com/site/about/guidelines.xhtml

Patient Costs and Opportunities Questionnaire

Please complete the below questionnaire by ticking ONE box to answer each question.

1. In the last 12 months, have you signed up to any new programmes (e.g. gym membership, taken up a new activity like yoga/meditation) that might help your health?
   - ☐ No new programmes at all
   - ☐ Some new programmes
   - ☐ Lots of new programmes

Please note any programmes you took up:

___

___

2. Have any new programmes that you have signed up to (e.g. gym membership, taken up a new activity) cost you any money that you might have spent elsewhere?
   - ☐ Yes — Total Amount: ____ Weekly amount: ____ (if you don't know, please estimate)
   - ☐ No

Please only answer the below questions if you registered with the My Breathing Matters website in the last 12 months.

3. Did you think there were any benefits of using My Breathing Matters?
   - ☐ No benefit at all
   - ☐ Very little benefit
   - ☐ Some benefit
   - ☐ Quite a bit of benefit
   - ☐ A large amount of benefit

If any benefits, please note them down below:

___

___

4. Did you think there were any disadvantages of using My Breathing Matters?
   - ☐ No disadvantages at all
   - ☐ Very little disadvantages
   - ☐ Some disadvantages
   - ☐ Quite a bit of disadvantages
   - ☐ A large amount of disadvantages

If any disadvantages, please note them down below:

___

___

5. How likely are you to recommend My Breathing Matters to friends and family if they needed similar care and treatment?
   - ☐ Extremely likely
   - ☐ Likely
   - ☐ Neither likely or unlikely
   - ☐ Unlikely
   - ☐ Extremely unlikely
   - ☐ Don't know

For peer review only - http://bmjopen.bmj.com/site/about/guidelines.xhtml

Appendix 2. Time and costs Questionnaire summary

	Demographic characteristic	
	Intervention	Usual Care
In the last 12 months, have you signed up to any new programmes (e.g. gym membership, taken up a new activity like yoga/meditation) that might help your health?	Lots of new programmes (2%)<br>Some new programmes (27%)<br>No new programmes (50%)<br>Missing (21%)	Lots of new programmes (0%)<br>Some new programmes (36%)<br>No new programmes (69%)<br>Missing (4%)
Have any new programmes that you have signed up to (e.g. gym membership, taken up a new activity) cost you any money that you might have spent elsewhere?	Yes (14%)<br>No (64%)<br>Missing (23%)	Yes (21%)<br>No (68%)<br>Missing (11%)
Total amount spent on other programmes (M, SD)	363.75 (320.30)	336.25 (379.81)

For peer review only

My Breathing Matters

UNIVERSITY OF Southampton

My Breathing Matters – Trial Protocol

'My Breathing Matters' - feasibility study of a digital self-management programme designed to improve the quality of life people with asthma.

Version 2, dated 07/12/2016

SPONSOR: University of Southampton

COORDINATING CENTRE: Centre for Applications of Health Psychology (supported by Southampton Clinical Trials Unit)

EudraCT reference no:	N/A
ISRCTN reference no:	TBC
**NHS Ethics reference no:**	**214457**

Protocol authorised by:

**Name:**	Prof. Lucy Yardley	**Role:**	Chief Investigator
**Signature:**	L Yardley	**Date:**	**07/12/2016**

For peer review only

MAIN TRIAL CONTACT

Chief Investigator: Professor Lucy Yardley

Centre for Applications of Health Psychology
Academic unit of psychology
University of Southampton
Southampton Tel: +44 (0)2380 594581
SO17 1BJ Email: L.Yardley@soton.ac.uk

TRIAL COORDINATION CENTRE

For general trial and clinical queries e.g. participant queries, trial supplies, data collection, please contact in the first instance:

Ben Ainsworth

Address: Centre for Applications of Health Psychology
Academic unit of psychology Tel:
University of Southampton Fax: 02380 591950
Southampton Email: ben.ainsworth@soton.ac.uk
SO17 1BJ Web:

SPONSOR

The University of Southampton is the research sponsor for this trial. For further information regarding sponsorship conditions, please contact:

Address: Diana Galpin
Head of IP, Contracts & Policy
University of Southampton
Southampton
SO17 1BJ

Tel: 02380 595058
Email: rgoinfo@soton.ac.uk

FUNDER
This trial is funded by NIHR PGfAR

Protocol Information
This protocol describes the My Breathing Matters trial and provides information about procedures for entering participants. The protocol should not be used as a guide for the treatment of other participants; every care was taken in its drafting, but corrections or amendments may be necessary. These will be circulated to investigators in the trial, but sites entering participants for the first time are advised to contact the Centre for Applications of Health Psychology to confirm they have the most recent version.

Compliance
This trial will adhere to the principles outlined in the International Conference on Harmonisation Good Clinical Practice (ICH GCP) guidelines. It will be conducted in compliance with the protocol, the Data Protection Act and all other regulatory requirements, as appropriate.

For peer review only

For peer review only - http://bmjopen.bmj.com/site/about/guidelines.xhtml

TABLE OF CONTENTS

LIST OF ABBREVIATIONS 4
KEYWORDS 4
TRIAL SYNOPSIS 5
SCHEDULE OF OBSERVATIONS AND PROCEDURES 8
1. INTRODUCTION 9
1.1 BACKGROUND 9
1.2 RATIONALE AND RISK BENEFITS FOR CURRENT TRIAL 9
2. TRIAL OBJECTIVES 10
3. TRIAL DESIGN 10
3.1 TRIAL OUTCOME MEASURES 11
4. CENTRE/PARTICIPANT SELECTION AND ENROLLMENT OF PARTICIPANTS 12
4.1 CENTRE SELECTION 12
4.2 SCREENING AND PRE-REGISTRATION 13
4.3 INCLUSION CRITERIA 13
4.4 EXCLUSION CRITERIA 14
4.5 REGISTRATION / RANDOMISATION PROCEDURES 14
4.6 CONSENT 14
4.7 WITHDRAWAL CRITERIA 15
6. INTERVENTION 15
6.1 INTERVENTION ARM 15
6.2 USUAL CARE 16
7. ADVERSE EVENTS AND REPORTING 16
7.1 DEFINITIONS 16
7.2 CAUSALITY 17
7.3 REPORTING PROCEDURES 17
7.3.2. Non serious AEs 17
7.3.3 Serious AEs 18
8. ASSESSMENT AND FOLLOW-UP OF PARTICIPANTS 19
8.1 DEFINITION OF END OF TRIAL 19
9. QUANTITATIVE EVALUATION 19
9.1 SAMPLE SIZE 19
9.2 RANDOMISATION 19
9.3 STATISTICAL PLAN INCLUDING INTERIM ANALYSIS 20
10. QUALITATIVE EVALUATION 20
11. REGULATORY ISSUES 21
11.1 CLINICAL TRIAL AUTHORISATION 21
11.2 ETHICS APPROVAL 21
11. 3 CONFIDENTIALITY 21

For peer review only - http://bmjopen.bmj.com/site/about/guidelines.xhtml

11.4 INDEMNITY **22**

11.5 SPONSOR **22**

11.6 FUNDING **22**

11.7 DEVIATIONS AND SERIOUS BREACHES **22**

11.8 AUDITS AND INSPECTIONS **23**

12. TRIAL MANAGEMENT **23**

13. PUBLICATION POLICY **23**

14. REFERENCES **24**

LIST OF ABBREVIATIONS

AE	Adverse Event
BMQ	Beliefs about Medication Questionnaire
CRF	Case Report Form
CTCAE	Common Terminology Criteria for Adverse Events
DI	Digital Intervention
DMEC	Data Monitoring and Ethics Committee
EQ-5D	EuroQol (health-related quality of life)
HCP	Healthcare Practitioner
ICH GCP	International Conference on Harmonisation of Good Clinical Practice
IMP	Investigational Medicinal Product
MARS	Medication Adherence Report
MBM	My Breathing Matters
NIHR	National Institute for Health Research
PCRN	Primary Care Research Network
PGfAR	Programme Grants for Applied Research
QIPP	Quality, Innovation, Productivity and Prevention
R&D	Research & Development
REC	Research Ethics Committee
SAE	Serious Adverse Event
SSI	Site Specific Information
TMG	Trial Management Group
TSC	Trial Steering Committee

KEYWORDS

Asthma; Self-management; Digital intervention

For peer review only - http://bmjopen.bmj.com/site/about/guidelines.xhtml

TRIAL SYNOPSIS

Title:	My Breathing Matters trial protocol
Sponsor:	Southampton University
Sponsor Ref Number:	TBC
Funder:	NIHR PGfAR
Trial Phase:	Complex intervention phase 1
Indication:	Asthma
Primary Objective:	1. To assess the feasibility and acceptability of a trial of My Breathing Matters (MBM) (a digital intervention designed to improve quality-of-life outcomes for people with asthma).
Secondary Objectives:	1. To assess feasibility of trial procedures including: clinical research governance, recruitment strategy, trial documents (e.g. PIS), eligibility criteria, consent/withdrawal, randomisation & blinding.<br>2. To assess feasibility and acceptability of MBM intervention including: usage & engagement, adherence and completion, fidelity of providers, participant retention.<br>3. To assess feasibility of data collection and analysis procedures, and look at data quality, management of trial data and estimates of effect size across trial outcome measures (see primary/secondary endpoints) to inform sample size calculations for a larger phase 3 RCT.
Tertiary Objectives	1. To examine intervention users' usage, engagement and possible mediators of behaviour change to inform future intervention design for well-powered usage and process analyses.
Trial Design:	Individually randomised controlled trial (assessor blinded)
Sample size : (split by treatment group)	80 participants (40 per arm)
Inclusion Criteria:	1. Age 18+ years.<br>2. Physician diagnosed asthma in medical record<br>3. ≥1 anti-asthma medication prescription in the previous year (determined from the physician prescribing records)<br>4. Impaired asthma-related health status (Asthma Quality of Life Questionnaire score of <5.5)<br>5. Informed consent<br>6. Able to access the internet and understand written english.
Exclusion Criteria:	1. Asthma judged at the baseline assessment to be dangerously unstable and in need of urgent medical review (if unstable asthma is found, the patient will be referred back to usual primary care clinician for review)<br>2. Terminal disease or other condition which in the opinion of the family doctor makes them inappropriate to take part<br>3. Diagnosed with 'difficult asthma' as defined by BTS.<br>4. Documented diagnosis of Chronic Obstructive Pulmonary Disease (COPD)

For peer review only - http://bmjopen.bmj.com/site/about/guidelines.xhtml

	5. Household member already enrolled on the study
Intervention	My Breathing Matters
Control Group:	Usual care
Primary Endpoints:	Feasibility and acceptability of intervention and trial procedures, including:<br>1. Uptake<br>2. Adherence<br>3. Completion rates
Secondary Endpoints:	Feasibility of measuring (and estimates of effect size to perform sample size calculations) in the following trial measures:<br>1. Asthma-specific Quality of life (AQLQ; short version)<br>2. Asthma control (ACQ)<br>3. Lung function (FEV_1, FEV_1/FVC, PEFR)<br>4. Quality of life (EQ5D, ICECAP-A)<br>5. Anxiety and depression (HADS)<br>6. Patient enablement (PEI)<br>7. Patient burden – time and costs.<br>8. Health resource use (professional contacts, referrals, prescriptions)<br>9. Adherence to recommendations
Tertiary Endpoints	Measurement of patient intervention group only:<br>1. Lifestyle change choice<br>2. Intervention usage and progress<br>3. Engagement with program and reasons for such engagement.
Follow up duration	12 months
Total Number of Sites :	Pilot: 4-6 sites

For peer review only

For peer review only - http://bmjopen.bmj.com/site/about/guidelines.xhtml

Participant flow diagram

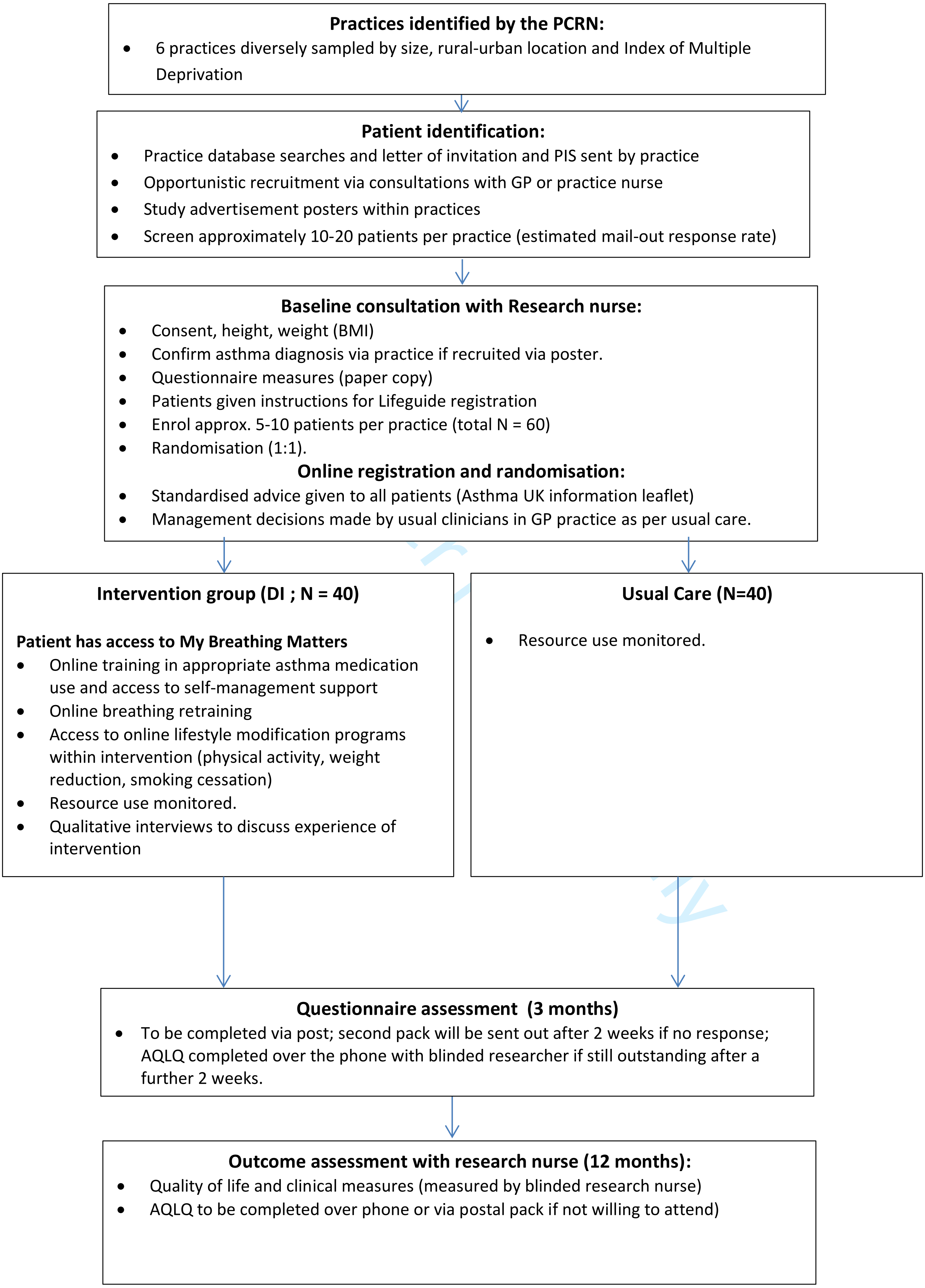

For peer review only - http://bmjopen.bmj.com/site/about/guidelines.xhtml

SCHEDULE OF OBSERVATIONS AND PROCEDURES

	Time point			
Measure	Baseline/ Screening	3-month follow up	Followup	Within the MBM programme
**Month**	0	3	12	(0-12)
Patient socio-demographic measures	X			
Clinical measures				
Weight (kg)	X	X	X	
Height (cm)	X			
Medication changes (prescriptions issued)			X (NR)	
Consultations			X (NR)	
Patient self-report measures				
Patient Enablement Instrument	X	X	X	
Asthma-specific Quality of life	X	X	X	
Asthma control	X	X	X	
Anxiety & depression	X	X	X	
Quality of life	x	X	X	
Medication adherence	X	X	X	
Lung function	X		X	
Medication use	X		X	
Patient objectively recorded measures				
Website usage				X*
Usage of asthma action plan (PAAP)				X*
Usage of asthma review pages				X*
Booked asthma review				X*
Usage of '4-week medication challenge'				X*
Usage of medication information				X*
Usage of breathing retraining challenge				X*
Usage of friends & family section				X*
Usage of stress reduction				X*
Choice of lifestyle changes				X*
Reported progress on lifestyle change (e.g. weight change)				X*
Economic measures				
Patient quality of life	X	X	X	
Patient costs	X	X	X	
Patient time and resource use			X (NR)	
Qualitative analysis				
Patient experience and views of the DI				X

Key:

NR = Notes review

*My Breathing Matters arm only – measured via Lifeguide website

1 This will be measured directly before and after the My Breathing Matters training completion

For peer review only

For peer review only - http://bmjopen.bmj.com/site/about/guidelines.xhtml

1. INTRODUCTION

1.1 BACKGROUND

The UK has one of the highest prevalence's of asthma in the world; nearly 6% of the UK population have asthma, comprising 5.4 million people, most of whom are managed in primary care[1]. Hospital admission and mortality rates for asthma showed improvements in the last decades of the last century, but these improvements have stalled since the millennium. Premature mortality from asthma was 1.5 times as high in the UK as in the rest of the EU in 2008, with around 1000 to 1200 deaths a year recorded since 2000. It is estimated that 90% of deaths are associated with preventable factors. Asthma is associated with high numbers of admissions and Emergency Department attendances, and it is estimated that 70% of these could have been prevented by appropriate early intervention and self-management[1]. Surveys of asthma symptoms and health status impairment continue to show that sub-optimal control is common and that at any given time the majority of asthmatics in the UK suffer potentially avoidable symptoms and quality of life impairment[2].

Although the UK leads the world in providing guidelines for asthma management, these have been poorly implemented and people with asthma do not receive evidence-based interventions, particularly individual action plans, which are known to impact positively on outcomes[3]. Patient education and proactive self-management have been convincingly shown to improve clinical outcomes in asthma[3] and have been advocated in guidelines for 20 years[4]. People with asthma without a management plan are four times more likely to have an asthma attack needing emergency care in hospital[5]. However, a representative (Ipsos-MORI) survey carried out by Asthma UK in 2010[6] suggested that only a quarter of people with asthma in the UK have a self-management plan. Self-management in asthma can also encompass non-pharmacological interventions to improve control and empower the patient, such as breathing exercises or lifestyle changes such as smoking cessation and weight reduction (since smoking and obesity are associated with worse prognosis in asthma[7]).

1.2 RATIONALE AND RISK BENEFITS FOR CURRENT TRIAL

Increasingly widespread access to the internet and mobile phones [8,9] means that healthcare Digital Interventions (DIs) are accessible to the majority of patients, and can be used to provide information and support at any time the patient needs it [10]. DIs can empower patients by providing better access to personalised information, and support for active involvement in treatment and self-management [10]. A large meta-analysis found a small but significant positive effect of DIs on health-related behaviours [11], whilst a Cochrane review found evidence that computer-based health interventions for those with chronic health conditions significantly improved knowledge, health behaviours and clinical outcomes [12]. DIs have the potential to make significant savings by automating routine aspects of patient education, monitoring and support, freeing up health professional resources for when patients most need them[13]. These savings can play an essential part in meeting the NHS QIPP agenda to achieve increased efficiency gains despite the growing demand created by an expanding and ageing population.

For peer review only - http://bmjopen.bmj.com/site/about/guidelines.xhtml

There is accumulating evidence that DIs can deliver better and more efficient healthcare in the context of asthma. A recent systematic review by our group found that self-management DIs could improve asthma control and reduce asthma-related quality of life impairment but called for larger, more robust trials[14]. We identified several existing self-management DIs for asthma, but half of these were for children, and many were not in English. While there are numerous commercial DIs for asthma, only one has been evaluated[15]. The RAISIN pilot trial demonstrated that self-management interventions could be effective at improving quality of life and asthma control, with improvements to 'reach' and response rate by catering to patients with mild asthma but impaired quality of life through non-pharmacological means [16,17]. A Danish trial compared GP and specialist care with web-based self-monitoring with automated feedback and a stepped care medication plan (with GP advice when required)[15]. After six months those allocated to the web-based self-monitoring had greater improvement in symptoms, quality of life and lung function. The SMASHING trial in the Netherlands[18] compared usual care with web-based educational resources, self-monitoring and automated feedback on medication titration, plus some group and email nurse support. At one year the intervention group had better quality of life and lung function and more symptom free days, at no extra cost[19].

2. TRIAL OBJECTIVES

The primary aim of the My Breathing Matters trial is to assess the feasibility, acceptability, effectiveness and cost-effectiveness of a DI in primary care for the self-management of asthma, in comparison to usual care (with provision of standard patient information materials produced by the charity Asthma UK).

Main research question:

1. To assess the feasibility and acceptability of a trial of My Breathing Matters, an intervention designed to assess improvements in clinical outcomes (e.g. quality of life, health resource use, lung function) of people with asthma.

Secondary research questions

1. To assess feasibility of trial procedures including: clinical research governance, recruitment strategy, trial documents (e.g. PIS), eligibility criteria, consent/withdrawal, randomisation and blinding.
2. To assess feasibility and acceptability of MBM intervention including: usage and engagement, adherence and completion, fidelity of providers.
3. To assess feasibility of data analysis, including data collection, data quality, management of trial data and estimates of effect size across trial outcome measures (see primary/secondary endpoints) to inform sample size calculations for a larger phase 3 RCT.

Tertiary research questions

1. To examine intervention usage, progress and engagement to inform well-powered usage analysis in larger trial

3. TRIAL DESIGN

For peer review only - http://bmjopen.bmj.com/site/about/guidelines.xhtml

My Breathing Matters is a digital intervention for the self-management of asthma, consisting of pharmacological support, (advice about asthma reviews and personal asthma action plans, information about medication and side effects) and non-pharmacological components (stress reduction, online versions of breathing retraining courses shown to be acceptable and feasible[20]) and optional user-selected lifestyle modifications. 80 participants will be randomised either to the My Breathing Matters programme or to a control group receiving usual care with provision of an Asthma UK information leaflet. This trial will be coordinated from UK facilitated by the PCRN's in these areas with researchers and research nurses employed at each centre.

The MBM study will comprise:

1) **Feasibility trial**: 80 participants (40 per arm) will be recruited from practices to confirm the acceptability and feasibility of the intervention, full trial protocol and study procedures.
2) **Qualitative analysis** will also be embedded in to the My Breathing Matters study.

3.1 TRIAL OUTCOME MEASURES

The primary outcome of the trial will be the feasibility and acceptability of a trial of My Breathing Matters, an intervention designed to improve clinical outcomes (e.g. symptom control, quality of life) of people with asthma.

Secondary outcomes will be to assess the feasibility of measuring (and estimates of effect size to generate hypotheses and perform sample size calculations with which to test them) in the following trial measures:

1. Asthma-specific Quality of life (AQLQ; short version)
2. Asthma control (ACQ)
3. Lung function (FEV_1, FEV_1/FVC, PEFR)
4. Quality of life (EQ5D)
5. Anxiety and depression (HADS)
6. Patient enablement (PEI)
7. Perceived support
8. Costs of equipment and drugs
9. Health resource use (professional contacts, referrals, prescriptions)
10. Adherence to recommendations
11. Engagement with program and reasons for such engagement

3-month questionnaire packs will be mailed out with a freepost return envelope. Baseline and 12-month follow up questionnaires will be taken using paper questionnaire packs and clinical measures will be taken by a research nurse.

For 3 month and 12-month measures, a second pack will be sent out after 2 weeks if no response; followed by questionnaire measures (AQLQ) completed over the phone if still outstanding after a further 2 weeks.

<u>Further detail regarding clinical measures:</u>

- Lung function will be measured using spirometry by an appropriately trained research nurse. Measures of lung function will be: FEV_1 (forced expiratory volume in 1 second), FEV_1/FVC (ratio of FEV_1 to forced vital capacity), PEFR (peak expiratory flow rate).

For peer review only - http://bmjopen.bmj.com/site/about/guidelines.xhtml

- Medication prescriptions, any changes and support provision (consultations) will be identified from patient notes reviews after completion of the 12-month follow-up. Medication use will be converted into defined daily doses of medication.

Further detail regarding patient reported measures:

- Asthma-specific quality of life (AQLQ) at 12-month follow-up
- Asthma symptoms will be measured using the Asthma Control Questionnaire (ACQ)
- Anxiety and depression will be measured using the Hospital Anxiety and Depression inventory (HADS)
- Patient enablement (PEI): will be measured using the Patient Enablement Instrument [31].
- Patient medication adherence: all participants will complete the MARS as a measure of medication adherence [2].

In the event that the results from any of the questionnaires are of concern (e.g. demonstrate significant anxiety or depression), the research team will inform the participant of their scores and the normal range of scores, and suggest that they may wish to discuss this result with their GP.

Economic measures:

- Health resource use (professional contacts, referrals, prescriptions)
- Patient time/burden needed to take part in intervention
- Health-related quality of life: the EuroQol (EQ-5D 5L) provides a measure of quality of life for economic analysis[5] and the ICEACAP-A measures non-health-specific quality of life in adults[21].
- Patient costs (e.g. additional costs incurred if patient engages in extra activities beyond intervention, such as gym membership)

Tertiary outcomes (intervention group only) will include:

- Lifestyle change choice, usage and progress
- Adherence to recommendations
- Engagement with program and reasons for such engagement

All measured over the 12 months of the study.
Assessed at baseline, after 3 months and after 12 months of online training.

Socio-demographic data

- Participants' age, gender, internet experience, disease status (time diagnosed), smoking habits, education level, and social deprivation indices based on postcode will be recorded at baseline

4. CENTRE/PARTICIPANT SELECTION AND ENROLLMENT OF PARTICIPANTS

4.1 CENTRE SELECTION

For peer review only

For peer review only - http://bmjopen.bmj.com/site/about/guidelines.xhtml

Eligible participants will be identified from around 6 general practices located by primary care research networks (PCRN); Practices will be selected in order to confirm feasibility for a wider trial. Principal Investigators will be identified at the regional level, with lead GPs nominated at each site.

The following documents must be in place and copies sent to the MBM Clinical Trial Coordinator (CTC) (see contact details on page 2):

- The approval letter from the relevant R&D Department, following submission of the Site Specific Information (SSI) form (where required)
- A signed Study Agreement (PI and sponsor signature)
- Completed Signature List and Roles and Responsibilities document
- Completed contacts list of all site personnel working on the Study

Upon receipt of all the above documents, the MBM CTC will send a confirmation letter to the lead GP. This letter must be filed in each centre's Site File. Along with this confirmation letter, the practice should receive their trial supplies and a study pack holding all the documents required to recruit a patient into the MBM Trial.

4.2 SCREENING AND PRE-REGISTRATION

Screening

- Electronic database searches will be conducted, and records screened by general practitioners to remove participants who meet exclusion criteria. The latest version of the participant information sheets along with the consent and screening questionnaire (including a freepost response envelope) will be posted with a letter inviting the patient to take part in the study;
- Eligible patients may also be identified and referred opportunistically during routine consultations with the practice nurse or GP.
- Study advertisements will be displayed in participating practices;
    - Patients responding to the study advertisements will phone the research team who will conduct a minimal screening of participants by telephone using a standard set of questions (included as a supporting document) to establish whether they meet the basic inclusion criteria, e.g. diagnosis of asthma, access to internet. The research team will then send copies of patient study materials (consent form and questionnaire) to confirm eligibility.

Patients who do not respond within one month of the practice invite will be followed up by telephone. This procedure will be explicitly outlined in the invite letter sent from the practice. In addition, all invited participants will be provided with a response form to return should they wish to decline to take part in the trial. This will ask for basic demographic information and their reasons for declining, including an option not to give a reason if they prefer not to (see patient opt-out).

4.3 INCLUSION CRITERIA

1. Physician diagnosed asthma in medical record (confirmed via practice)
2. ≥1 anti-asthma medication prescription in the previous year (determined from the physician prescribing records)

For peer review only - http://bmjopen.bmj.com/site/about/guidelines.xhtml

3. Impaired asthma-related health status (Asthma Quality of Life Questionnaire score of <5.5)
4. Informed consent
5. Able to understand English and access internet.

4.4 EXCLUSION CRITERIA

6. Asthma judged at the baseline assessment to be dangerously unstable and in need of urgent medical review (if unstable asthma is found, the patient will be referred back to usual primary care clinician for review)
7. Terminal disease or other condition which in the opinion of the family doctor makes them inappropriate to take part
8. Diagnosed with 'difficult asthma' as defined by BTS.
9. Documented diagnosis of Chronic Obstructive Pulmonary Disease (COPD)
10. Household member already enrolled on the study

4.5 REGISTRATION / RANDOMISATION PROCEDURES

Informed consent

All patients will receive a copy of the consent form in their invitation letter pack, and will be asked to complete it when contacting the research team. All patients will receive a face-to-face baseline consultation with the practice nurse where additional informed consent (see consent form) will be recorded before clinical measures, such as confirmation of asthma diagnosis, are undertaken (see primary outcomes data document).

Randomisation

Patients will be randomised at the practice where if in the intervention group they will be given access to the My Breathing Matters programme, or if in the control group will be given Asthma UK information leaflet. (see section 9.2 for further details of randomisation). Participants in the intervention group will be instructed to sign up to the My Breathing Matters programme upon which they will be notified by email of their involvement in the study. Only one patient per household will be randomised.

4.6 CONSENT

Consent to enter the trial must be sought from each participant only after a full explanation has been given, an information leaflet offered and time allowed for consideration. Signed participant consent will be obtained. The right of the participant to refuse to participate without giving reasons will be respected. After the participant has entered the trial the clinician remains free to give alternative treatment to that specified in the protocol at any stage if he/she feels it is in the participant's best interest, but the reasons for doing so should be recorded. In these cases the participants remain within the trial for the purposes of follow-up and data analysis. All participants are free to withdraw at any time from the protocol intervention without giving reasons and without prejudicing further treatment.

Participants will give informed consent (including explicit consent to access relevant data from the DI and their medical records) before they first log onto MBM. They will be free to withdraw at any time simply by contacting the research team or practice staff

For peer review only - http://bmjopen.bmj.com/site/about/guidelines.xhtml

4.7 WITHDRAWAL CRITERIA

Patients will be withdrawn from the trial if:

- They are no longer eligible (specialist management of Asthma or illness precluding participation)
- They choose not to continue

All patients who withdraw from the intervention will be asked if they are prepared to attend the final follow-up appointment with the research nurse and/or complete self-report follow-up measures. If they agree, they will be invited according to the follow-up procedure and will have the option to answer follow up questionnaires without using the website.

.

6. INTERVENTION

6.1 INTERVENTION ARM

The **online My Breathing Matters programme** will comprise **three** main components:

1. *Intervention components*

The MBM programme will use behavioural techniques to improve functional quality of life of primary care patients with asthma, by supporting illness self-management by pharmacological and non-pharmacological means (thereby reducing risk of asthma exacerbation).

The program has three main design objectives: i) to engage people who do not view themselves as having active asthma, ii) to persuade and educate users to implement appropriate pharmacological management and iii) to encourage users to employ non-pharmacological methods of improving QoL. Key features to address these objectives include: i) maintaining positive illness context throughout (i.e. promote health rather than manage illness) and offering a simple, unobtrusive interface to provide optional (and flexible) support only when needed, ii) focusing on persuading/educating users regarding the necessity, efficacy and safety of preventative asthma medication, and facilitating easy completion of an action plan with primary care support, and iii) educating users on benefits and offer psychological methods to improve quality of life (e.g. cognitive behavioural techniques for symptom management), tailored access and address patient concerns about relevant positive lifestyle changes, such as weight-loss if overweight, smoking cessation if current smoker, physical activity if inactive.

Patients in the intervention group will be offered optional support from Asthma UK helpline – as a source of asthma advice and support for those who would like personal contact outside of that offered by MBM or their healthcare team, to support self-monitoring and lifestyle modification. This support would be the same as that offered to anyone who calls (outside of MBM) so the Asthma UK nurse team would not require additional training.

For peer review only - http://bmjopen.bmj.com/site/about/guidelines.xhtml

Motivating and reassuring support messages will be sent by email to all patients in the intervention group every 2 to 4 weeks; these will encourage and reinforce patient adherence to lifestyle changes (where applicable).

6.2 Usual Care

All patients will be able to access the Asthma UK information leaflet following randomisation. Patients within the usual care condition will be informed that they should simply follow their usual instructions for asthma management. After the patients have completed the 12-month follow-up they will be given access to the intervention, in line with recommendations from PPI representatives.

7. ADVERSE EVENTS AND REPORTING

7.1 DEFINITIONS

Adverse Event (AE): any untoward medical occurrence in a participant or clinical study participant which does not necessarily have a causal relationship with study treatment or participation.

Serious Adverse Event (SAE) or Serious Adverse Reaction: any untoward medical occurrence or effect that at any dose:

- Results in death
- Is life-threatening – refers to an event in which the participant was at risk of death at the time of the event; it does not refer to an event which hypothetically might have caused death if it were more severe
- Requires hospitalisation, or prolongation of existing hospitalisation
- Results in persistent or significant disability or incapacity
- Is a congenital anomaly or birth defect
- Other important medical events***.

*'life-threatening' in the definition of 'serious' refers to an event in which the patient was at risk of death at the time of the event; it does not refer to an event which hypothetically might have caused death if it were more severe.

**Hospitalisation is defined as an inpatient admission, regardless of length of stay, even if the hospitalisation is a precautionary measure for continued observation. Hospitalisations for a pre-existing condition, including elective procedures that have not worsened, do not constitute an SAE.

***Other important medical events that may not result in death, be life-threatening, or require hospitalisation may be considered a serious adverse event/experience when, based upon appropriate medical judgment, they may jeopardise the participant and may require medical or surgical intervention to prevent one of the outcomes listed in this definition.

Note: It is the responsibility of the PI or designated to grade an event as 'not serious' (AE) or 'serious' (SAE).

For peer review only - http://bmjopen.bmj.com/site/about/guidelines.xhtml

7.2 CAUSALITY

The assignment of the causality to trial procedures of any serious event should be made by the investigator responsible for the care of the participant using the definitions in the table below.

If any doubt about the causality exists the local investigator should inform the trial coordinator who will notify the Chief Investigator. Other clinicians may be asked for advice in these cases.

In the case of discrepant views on causality between the investigator and others, all parties will discuss the case. In the event that no agreement is made, the Ethics Committee will be informed of both points of view.

Relationship	Description
**Unrelated**	There is no evidence of any causal relationship
**Unlikely**	There is little evidence to suggest there is a causal relationship (e.g. the event did not occur within a reasonable time after administration of the trial medication). There is another reasonable explanation for the event (e.g. the participant's clinical condition, other concomitant treatment).
**Possible**	There is some evidence to suggest a causal relationship (e.g. because the event occurs within a reasonable time after administration of the trial medication). However, the influence of other factors may have contributed to the event (e.g. the participant's clinical condition, other concomitant treatments).
**Probable**	There is evidence to suggest a causal relationship and the influence of other factors is unlikely.
**Definitely**	There is clear evidence to suggest a causal relationship and other possible contributing factors can be ruled out.

For peer review only

7.3 REPORTING PROCEDURES

All adverse events should be reported. Depending on the nature of the event the reporting procedures below should be followed. Any questions concerning adverse event reporting should be directed to the SCTU in the first instance.

7.3.1. Pre-existing Conditions

A pre-existing condition should not be reported as an AE unless the condition worsens by at least one CTCAE grade during the trial. The condition, however, must be reported in the pre-treatment section of the CRF, if symptomatic at the time of entry, or under concurrent medical conditions if asymptomatic.

7.3.2. Non serious AEs

All adverse events that may be related to the study will be recorded in the relevant case report form and Adverse Event form and sent to the SCTU within one month of the form being due. As adults on average see their GP approximately 5 times per year for a variety of routine and unscheduled appointments (e.g. for medication review, self-limiting minor illnesses and long-

For peer review only - http://bmjopen.bmj.com/site/about/guidelines.xhtml

term conditions unrelated to asthma), many medical encounters are of no relevance to the study. Events that will be recorded include any judged by the study nurse to be possibly related to the study. In particular, all medical encounters related to the following medical areas or symptoms will be recorded in the Adverse Events form:

Psychological morbidity: any events relating to anxiety, depression or mood disorders
Respiratory morbidity: any events relating to breathing or chest symptoms
Musculoskeletal, Abdominal and chest pain: any events relating to pain in these systems unless known to be associated with an unrelated pre-existing condition.

The study nurses are advised to record any event for which there is uncertainty as to whether it is study related or not, and to discuss with the local PI or CI.

7.3.3 Serious AEs

All SAEs (including those that are expected and related) will be reported within 24 hours of the local site becoming aware of the event. The SAE form asks for nature of event, date of onset, severity, corrective therapies given or action taken, outcome and relatedness (i.e. unrelated, unlikely, possible, probably, definitely). The responsible centre Principal Investigator will assign the relatedness and expectedness of the event. Additional information will be provided as soon as possible if the event has not resolved at the time of reporting.

A flowchart is given below to illustrate reporting procedures:

- GPs or nurses will be asked to notify us via an SAE form if a participant experiences any SAEs.
- The Sponsor and main Research Ethics Committee (REC) will be informed of all related SAEs occurring during the trial according to the following timelines, where day zero is defined as the date the SAE form is initially received:
- Events which are fatal or life-threatening will be reported no later than 7 calendar days after the sponsor is first aware of the reaction. Any additional relevant information must be reported within a further 8 calendar days.
- Events that are non-fatal or non life-threatening will be reported within 15 calendar days of the sponsor first becoming aware of the reaction.
- All Investigators will be informed of all related SAEs occurring throughout the trial. Local Investigators should report any SAEs as required by their Local Research Committee and/or Research and Development Office.

7.3.4 Follow Up and Post-study Serious Adverse Events

The reporting requirement for SAEs affecting participants applies for all events occurring up to the end of the last treatment. All unresolved adverse events should be followed by the local investigator until resolved, the participant is lost to follow-up, or the adverse event is otherwise explained. At the last scheduled visit, the investigator should instruct each participant to report any subsequent event(s) that the participant, or the participant's general practitioner, believes might reasonably be related to participation in this study. The investigator should notify the Centre for Applications of Health Psychology of any death or adverse event occurring at any time after a participant has discontinued or terminated study participation that may reasonably be related to this study.

For peer review only - http://bmjopen.bmj.com/site/about/guidelines.xhtml

8. ASSESSMENT AND FOLLOW-UP OF PARTICIPANTS

Measures will be administered for all participants at baseline, 3 and 12 months, unless otherwise stated (see Schedule of Observations and Procedures). Patient-reported outcome measures will be completed online. Non-respondents will receive two email reminders followed by a paper-based copy accompanied by a final telephone follow-up. All website usage (MBM measures) will be recorded automatically in Lifeguide.

An independent research nurse, blind to study allocation will complete the follow-up assessments. All patients (including withdrawn participants who have consented to follow-up appointments) will follow the procedure outlined below:

- Approximately three weeks before the follow-up appointment is due, the patient will be sent a letter by the research team encouraging them to arrange an appointment with the research nurse. Appointments may be scheduled to take place in the patients' home or usual GP practice.
- If the patient does not contact the research team to arrange an appointment within two weeks of the letter being sent the patient will be followed up by telephone.
- The research team or research nurse will send confirmation of the follow-up appointment to the patient by telephone, text, email or letter (dependent on patient preference).
- If the patient indicates that they would not be willing to complete a follow-up, no further contact will be made with the patient regarding the follow-up appointment.

8.1 DEFINITION OF END OF TRIAL

The end of the trial will be defined as date of entry of final data into database.

9. QUANTITATIVE EVALUATION

9.1 SAMPLE SIZE

A minimum of 40 participants from two centres will be recruited to each condition during the study.

9.2 RANDOMISATION

Participants will be randomised in a 1:1 ratio to receive either usual care (control) or MBM programme with optional support (intervention). The randomisation will be stratified block randomisation by the average value of AQLQ scores in the BREATHE Trial (Thomas et al).

Remote allocation will maintain allocation concealment from both the participant and the research nurse prior to allocation, however the outcome assessor will be blinded to participant allocation.

For peer review only - http://bmjopen.bmj.com/site/about/guidelines.xhtml

9.3 STATISTICAL PLAN INCLUDING INTERIM ANALYSIS

A detailed statistical plan will be developed prior to data lock. No interim analysis is planned. Data and all appropriate documentation will be stored for a minimum of 10 years after the completion of the trial, including the follow-up period, in a secure location at Southampton University.

Descriptive statistics of outcome variables will be calculated in order to inform a future randomized controlled trial, including examining levels of missing data, attrition rate, retention and completion. Statistical comparisons between and within intervention and control groups will be performed to estimate variability in primary and secondary outcomes within a linear regression framework. Further exploratory analysis of patient engagement, intervention usage and possible mediators of behaviour change will be performed.

10. QUALITATIVE EVALUATION

10.1 Qualitative Evaluation

The qualitative study will be undertaken both alongside participation and after participants finish using MBM. Qualitative interviews will seek to provide an in-depth understanding of the perspective of patients, to inform intervention and trial acceptability and to generate hypotheses about intervention mechanisms of action that can be tested in a larger trial.

Participants and sampling

Patients will be asked whether they give permission to take part in an interview at a later point in the study. Purposive sampling will be used to select patients from the intervention group to allow for a wide range of views and experiences of the MBM programme. It is anticipated that 20 patients will be interviewed during and following the study, and patients will be selected for interviews until saturation is reached.

Interviews and qualitative analysis

Factors that may facilitate or diminish the acceptability of the MBM programme, and adherence to implementation will be explored across patient and health care professional interviews. Interviews will be conducted from after the 3-month assessment. Open-ended questions will be used to elicit user perspectives and experiences of the intervention, allowing participants to freely describe their experiences and views in their own way and to focus on whatever is most salient to them.

Interviews will be audio-recorded and fully transcribed. The findings will be used to inform any modifications needed to the MBM programme or the trial procedures for a potential future full RCT.

The transcriptions will be anonymised (identifiable data removed) and participants' transcripts will be given participant numbers so that they can be easily discussed between team members whilst protecting participants' identities. To ensure that we remain open to and grounded in users' perspectives we will carry out inductive thematic analysis[22] of all textual data,

For peer review only - http://bmjopen.bmj.com/site/about/guidelines.xhtml

triangulated where appropriate with quantitative self-report measures and web usage data, and constant comparison and discussion among team members to reach inter-rater agreement on themes and interpretations.

11. REGULATORY ISSUES

11.1 CLINICAL TRIAL AUTHORISATION

This trial does NOT involve the testing of any Investigational Medicinal Products (IMPs) therefore approval from the Medicines and Healthcare products Regulatory Agency is not required.

11.2 ETHICS APPROVAL

The trial protocol will be submitted to a Research Ethics Committee (REC) recognised by the United Kingdom Ethics Committee Authority (UKECA) for review and approval. A favourable opinion must be obtained before commencement of any trial procedures (including recruitment of patients) occurs

All substantial amendments must be approved by the REC responsible for the trial, in additional to approval by NHS R&D. Minor amendments will not require prior approval by the REC.

If the trial is stopped prematurely, it will not be recommended without reference to the REC responsible for the trial.

The outcome of the trial will be reported to the responsible REC within 90 days of completion of the last patient’s final trial procedures. In the event of the trial being prematurely terminated a report will be submitted to the REC within 15 days. A summary of the Trial Report will be submitted to the responsible REC within one year of completion of the last participant’s final study procedures.

The investigator must ensure that participant’s anonymity will be maintained and that their identities are protected from unauthorised parties. On CRFs participants will not be identified by their names, but by an identification code.

11. 3 CONFIDENTIALITY

Initial practice database searchers will be conducted on practice computer systems and subsequent study invitations will be the responsibility of the practice, to maintain the confidentiality of potential participants. Participants’ identification data will be required for the registration process, which will be completed with the support provider at the baseline screening appointment.

For peer review only - http://bmjopen.bmj.com/site/about/guidelines.xhtml

Participants will be informed that the research team will have access to their study data, and that personal information such as their name, telephone address and email address will be stored and used by the research team to stay in touch with them throughout the study.

The primary data outcomes measured by the support provider (such as asthma quality of life and current medication) will be stored on an independent, secure server. All other data (secondary self-report measures and intervention data) will be stored on dedicated secure spaces behind a firewall on password protected computer located in secure university buildings at the University of Southampton. The data is backed up daily. Confidentiality of all data entered into the DI will be maintained by following best practice in NHS IT protection and data security systems (e.g. regarding use of https, storing data behind the university firewall etc). Access to the website will be via username and password. The use of strong passwords will be enforced. The communication between the person entering data through a web browser and the server will be through a secure internet connection (HTTPS). The research team will adhere to the Data Protection Act 1998.

Digital records will be stored on a university computer in a password protected file. All data will be linked with the participant's study ID which does not include any personal or identifiable information, such as name or D.O.B. Study IDs will be linked with patient's names, email addresses telephone numbers in a separate, securely stored file.

At the end of the study, data will be anonymised and stored on a password protected computer located in secure university buildings and appropriately backed up. Confidentiality of all data entered into the DI will be maintained by following best practice in NHS IT protection and data security systems.

11.4 INDEMNITY

The sponsor of the trial is University of Southampton. University of Southampton insurance will apply. The lead authors of the protocol are the Chief Investigator (Prof Lucy Yardley), and Prof Mike Thomas, who are both university employees and as such will be covered by the University insurance.

11.5 SPONSOR

University of Southampton is acting as the sponsor for this trial. The My Breathing Matters coordinating team has been delegated duties by the Sponsor relating to: submissions to regulatory authorities and GCP.

11.6 FUNDING

NIHR PGfAR are funding this trial (RP-PG-1211-20001).

11.7 DEVIATIONS AND SERIOUS BREACHES

Any trial protocol deviations/violations and breaches of Good Clinical Practice occurring at sites should be reported to the MBM trial coordinator and the local R&D Office immediately.

For peer review only - http://bmjopen.bmj.com/site/about/guidelines.xhtml

The MBM trial coordinator will then advise of and/or undertake any corrective and preventative actions as required.

All serious protocol deviations/violations and serious breaches of Good Clinical Practice and /or the trial protocol will immediately be reported to the regulatory authorities and other organisations, as required in the Medicines for Human Use (Clinical Trials) Regulations 2004, as amended.

11.8 AUDITS AND INSPECTIONS

The trial may be participant to inspection and audit by University of Southampton, under their remit as sponsor, the trial coordinating centre as the Sponsor's delegate and other regulatory bodies to ensure adherence to ICH GCP, Research Governance Framework for Health and Social Care, applicable contracts/agreements and national regulations.

12. TRIAL MANAGEMENT

The Trial Management Group (TMG) is responsible for overseeing progress of the trial. The day-to-day management of the trial will be co-ordinated through the trial coordinating centre and oversight will be maintained by the Trial Steering Committee, with an experienced independent chair and representation of patient representatives, Asthma UK and Blood Pressure UK.

The Steering Committee will meet twice a year throughout the programme to provide strategic guidance and independent monitoring of progress and professional conduct. We will encourage in person attendance at all these meetings where possible, but will also provide for attendance by teleconference when necessary, and will circulate papers and minutes before and after meetings for communication with those who cannot attend for any reason.

13. PUBLICATION POLICY

All publications and presentations relating to the trial will be authorised by the Trial Management Group and will follow an agreed publication policy. Dissemination of our work will be via multiple pathways:
a) to the scientific community through presentation at national & international conferences and regular publication in highly cited and open access peer reviewed journals.
b) to clinical and academic colleagues via professional societies: links with the following societies will be exploited to raise the profile of this work: Royal College of GPs and Physicians, Society of Behavioural Medicine, British Sociological Society Medical Sub-group.
c) to patients via patient groups: We will work with Asthma UK to disseminate our results.
d) to participants: All participants will be sent an accessible summary of the findings from the study that they took part in within six months of study completion.
e) to relevant NHS organisations and healthcare providers (e.g. Clinical Commissioning Groups, NHS Choices, UCL partners).

For peer review only - http://bmjopen.bmj.com/site/about/guidelines.xhtml

f) to the public via local and national media: we will use regular press releases linked to dissemination events to ensure a high level awareness of our work in the media.
g) to all stakeholders via a dedicated website and through interactive workshops (with health professionals, patient groups, IT providers, commissioners, policy-makers, researchers).

14. REFERENCES

1. Department of Health. An outcomes strategy for people with chronic obstructive pulmonary disease (COPD) and asthma in England. 2011.
2. Demoly P, Gueron B, Annunziata K, Adamek L, Walters RD. Update on asthma control in five European countries: results of a 2008 survey. *European Respiratory Review* 2010;19:150-157.
3. Gibson PG, Powell H, Coughlan J et al. Self-management education and regular practitioner review for adults with asthma. *Cochrane Database of Systematic Reviews* 2002;3.
4. British Thoracic Society / Scottish Intercollegiate Guideline Network. British Guideline on the Management of Asthma. 2012. 22-8-0012.
5. Adams R, Smith B, Ruffin R. Factors associated with hospital admissions and repeat emergency dept visits for adults with asthma . *Thorax* 2000;55:566-573.
6. Asthma UK. National Asthma Panel 2010. 2010. London, Asthma UK.
7. Thomas M, Price D. Impact of comorbidities on asthma. *Expert Reviews in Clinical Immunology* 2008;4:731-742.
8. Ogedegbe G. Barriers to optimal hypertension control. *Journal of Clinical Hypertension* 2008;10:644-666.
9. Guthrie B, Inkster M, Fahey T. Tackling therapeutic inertia: role of treatment data in quality indicators. *Br Med J* 2007;335:542-544.
10. Ogedegbe G, Schoenthaler A. A systematic review of the effects of home blood pressure monitoring on medication adherence. *Journal of Clinical Hypertension* 2006;8:174-180.
11. Bosworth HB, Olsen MK, Grubber JM et al. Two Self-management Interventions to Improve Hypertension Control: A Randomized Trial. *Annals of Internal Medicine* 2009;151:687-695.
12. Agarwal R, Bills JE, Hecht TJW, Light RP. Role of home blood pressure monitoring in overcoming therapeutic inertia and improving hypertension control: a systematic review and meta-analysis. *Hypertension* 2011;57:29-38.
13. Abudagga A, Resnick HE, Alwan M. Impact of blood pressure monitoring on hypertension outcomes: a literature review. *Telemedicine and e-Health* 2010;16:830-837.
14. McLean G, Murray E, Band R et al. Interactive digital interventions to promote self-management in adults with asthma: systematic review and meta-analysis. *BMC Pulmonary Medicine* 2016:16:83.
15. Rasmussen LMPK, Nolte H, Backer V. Internet-based monitoring of asthma: A long-term, randomized clinical study of 300 asthmatic subjects. *Journal of Allergy and Clinical Immunology* 2005;115:1137-1142.
16. Morrison D, Wyke S, Saunderson K et al. Findings from a pilot randomised trial of an Asthma Internet Self-management Intervention *BMJ Open* 2016:6:e009254.
17. Morrison D, Mair F, Chaudhuri R et al. Details of development of the resource for adults with asthma in the RAISIN (randomized trial of an asthma internet self-management intervention) study. *BMC Medical Informatics & Decision Making* 2015; 15:57.
18. van der Meer V, Bakker MJ, van den Hout WB et al. Internet-based self-management plus education compared with usual care in asthma: A randomized trial. *Annals of Internal Medicine* 2009;151:110-120.
19. van der Meer V, van den Hout WB, Bakker MJ et al. Cost-Effectiveness of Internet-Based Self-Management Compared with Usual Care in Asthma. *PLoS ONE* 2011;6:e27108.

For peer review only - http://bmjopen.bmj.com/site/about/guidelines.xhtml

20. Yardley L, Ainsworth B, Arden Close E et al. The person-based approach to enhanceing the acceptability and feasibility of interventions, *Pilot & Feasibility Studies, 2015 : 1.*
21. Al-Janabi H, Flynn T, Coast J. (2012) Development of a self-report measure of capability wellbeing for adults: the ICECAP-A. *Quality of Life Research* 21: 167-176
22. Joffe H, Yardley L. Content and thematic analysis. In: Marks D, Yardley L, eds. *Research Methods for Clinical and Health Psychology*. London: Sage; 2004;56-68.

For peer review only

For peer review only - http://bmjopen.bmj.com/site/about/guidelines.xhtml

15. Trial Timeline

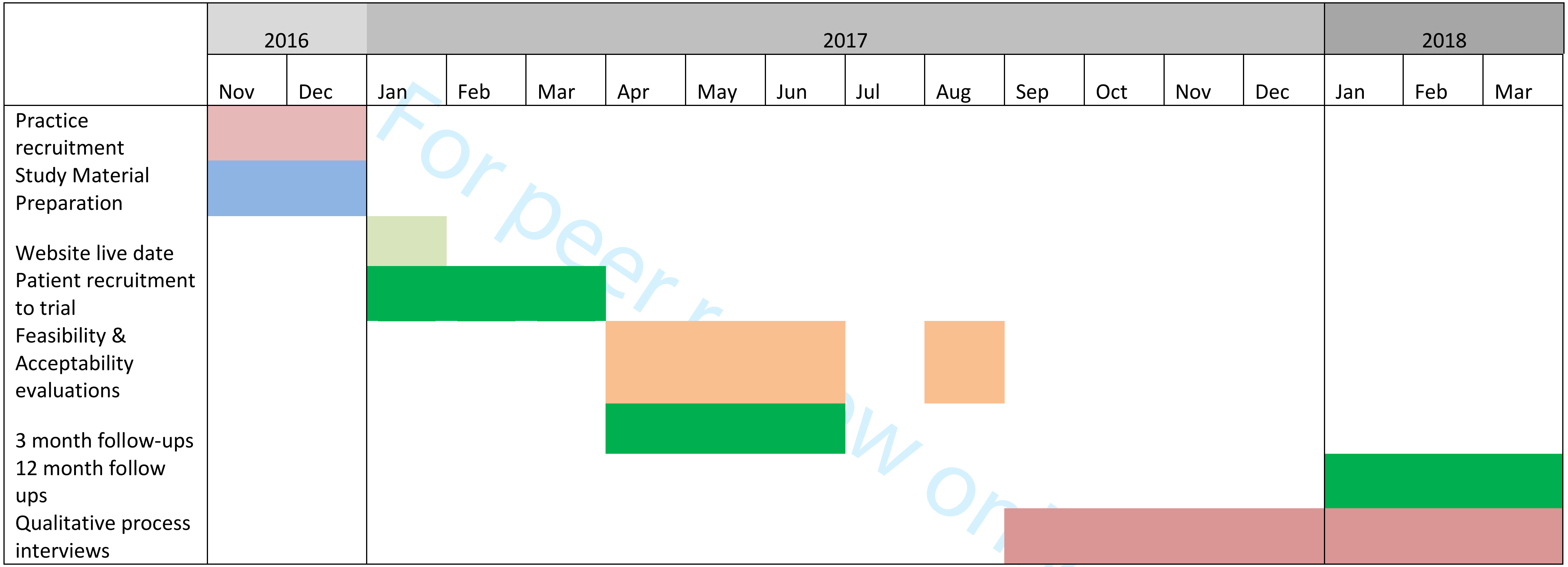

For peer review only - http://bmjopen.bmj.com/site/about/guidelines.xhtml

CONSORT 2010 checklist of information to include when reporting a pilot or feasibility trial*

Section/Topic	Item No	Checklist item	Reported on page No
**Title and abstract**			
	1a	Identification as a pilot or feasibility randomised trial in the title	1
	1b	Structured summary of pilot trial design, methods, results, and conclusions (for specific guidance see CONSORT abstract extension for pilot trials)	2
**Introduction**			
Background and objectives	2a	Scientific background and explanation of rationale for future definitive trial, and reasons for randomised pilot trial	6
	2b	Specific objectives or research questions for pilot trial	6
**Methods**			
Trial design	3a	Description of pilot trial design (such as parallel, factorial) including allocation ratio	7
	3b	Important changes to methods after pilot trial commencement (such as eligibility criteria), with reasons	-
Participants	4a	Eligibility criteria for participants	7
	4b	Settings and locations where the data were collected	7
	4c	How participants were identified and consented	7
Interventions	5	The interventions for each group with sufficient details to allow replication, including how and when they were actually administered	8
Outcomes	6a	Completely defined prespecified assessments or measurements to address each pilot trial objective specified in 2b, including how and when they were assessed	10
	6b	Any changes to pilot trial assessments or measurements after the pilot trial commenced, with reasons	-
	6c	If applicable, prespecified criteria used to judge whether, or how, to proceed with future definitive trial	-
Sample size	7a	Rationale for numbers in the pilot trial	8
	7b	When applicable, explanation of any interim analyses and stopping guidelines	-
Randomisation:			
Sequence generation	8a	Method used to generate the random allocation sequence	8
	8b	Type of randomisation(s); details of any restriction (such as blocking and block size)	8
Allocation concealment mechanism	9	Mechanism used to implement the random allocation sequence (such as sequentially numbered containers), describing any steps taken to conceal the sequence until interventions were assigned	8

Implementation	10	Who generated the random allocation sequence, who enrolled participants, and who assigned participants to interventions	8
Blinding	11a	If done, who was blinded after assignment to interventions (for example, participants, care providers, those assessing outcomes) and how	8
	11b	If relevant, description of the similarity of interventions	8
Statistical methods	12	Methods used to address each pilot trial objective whether qualitative or quantitative	13
**Results**			
Participant flow (a diagram is strongly recommended)	13a	For each group, the numbers of participants who were approached and/or assessed for eligibility, randomly assigned, received intended treatment, and were assessed for each objective	14
	13b	For each group, losses and exclusions after randomisation, together with reasons	14
Recruitment	14a	Dates defining the periods of recruitment and follow-up	14
	14b	Why the pilot trial ended or was stopped	-
Baseline data	15	A table showing baseline demographic and clinical characteristics for each group	Table 1
Numbers analysed	16	For each objective, number of participants (denominator) included in each analysis. If relevant, these numbers should be by randomised group	Table 2
Outcomes and estimation	17	For each objective, results including expressions of uncertainty (such as 95% confidence interval) for any estimates. If relevant, these results should be by randomised group	18
Ancillary analyses	18	Results of any other analyses performed that could be used to inform the future definitive trial	-
Harms	19	All important harms or unintended effects in each group (for specific guidance see CONSORT for harms)	19
	19a	If relevant, other important unintended consequences	-
**Discussion**			
Limitations	20	Pilot trial limitations, addressing sources of potential bias and remaining uncertainty about feasibility	24
Generalisability	21	Generalisability (applicability) of pilot trial methods and findings to future definitive trial and other studies	24
Interpretation	22	Interpretation consistent with pilot trial objectives and findings, balancing potential benefits and harms, and considering other relevant evidence	24
	22a	Implications for progression from pilot to future definitive trial, including any proposed amendments	23
**Other information**			
Registration	23	Registration number for pilot trial and name of trial registry	2
Protocol	24	Where the pilot trial protocol can be accessed, if available	4
Funding	25	Sources of funding and other support (such as supply of drugs), role of funders	4
	26	Ethical approval or approval by research review committee, confirmed with reference number	4

Citation: Eldridge SM, Chan CL, Campbell MJ, Bond CM, Hopewell S, Thabane L, et al. CONSORT 2010 statement: extension to randomised pilot and feasibility trials. BMJ. 2016;355.

*We strongly recommend reading this statement in conjunction with the CONSORT 2010, extension to randomised pilot and feasibility trials, Explanation and Elaboration for important clarifications on all the items. If relevant, we also recommend reading CONSORT extensions for cluster randomised trials, non-inferiority and equivalence trials, non-pharmacological treatments, herbal interventions, and pragmatic trials. Additional extensions are forthcoming: for those and for up to date references relevant to this checklist, see www.consort-statement.org.

For peer review only
